# *TAS1R2*/*TAS1R3* Single-Nucleotide Polymorphisms Affect Sweet Taste Receptor Activation by Sweeteners: The SWEET Project

**DOI:** 10.3390/nu17060949

**Published:** 2025-03-08

**Authors:** Christine Belloir, Mathilde Jeannin, Adeline Karolkowski, Loïc Briand

**Affiliations:** Centre des Sciences du Goût et de l’Alimentation, The National Centre for Scientific Research (CNRS), National Institute of Agricultural Research (INRAE), Institut Agro, Université Bourgogne Europe, F-21000 Dijon, France; christine.belloir@inrae.fr (C.B.); jeanninmathilde.ys@gmail.com (M.J.); adeline.karolkowski@inrae.fr (A.K.)

**Keywords:** TAS1R2/TAS1R3, SNP, sweet taste receptor, sweetener, obesity, sugar intake

## Abstract

Background/Objectives: Studies have hypothesised that single-nucleotide polymorphisms (SNPs) in the *TAS1R2* and *TAS1R3* genes may alter sweet compound detection and eating habits, thereby increasing the risk of obesity. This in vitro study aims to measure the impact of human *TAS1R2*/*TAS1R3* polymorphisms, some of which are thought to be involved in obesity, on the response of the sweet taste receptor to various sweeteners. It also aims to identify new SNPs in an obese population associated with a decrease in or loss of *TAS1R2/TAS1R3* function. Methods: First, the effects of 12 human *TAS1R2*-SNPs and 16 human *TAS1R3*-SNPs, previously identified in the literature, on the response of the sweet taste receptor stimulated by 12 sweeteners were investigated using functional cellular assays. Second, a total of 162 blood samples were collected from an obese population (BMI between 25 and 35 kg/m^2^) involved in the SWEET project. The TaqMan method for SNP genotyping was carried out using DNA extracted from blood samples to identify new SNPs and predict possible/probable TAS1R2/TAS1R3 loss of function. Results: Although certain human *TAS1R2*/*TAS1R3* SNPs showed reduced receptor response, they were not associated with particular phenotypes. Seven SNPs were predicted to severely impair the human TAS1R2/TAS1R3 response to sweeteners. Conclusions: Although some *TAS1R2*- and *TAS1R3*-SNPs have previously been associated with obesity, our cellular results do not confirm this association and reinforce the hypothesis, put forward by other researchers, that sweet taste perception and sugar consumption are governed by factors other than the *TAS1R2* and *TAS1R3* genes.

## 1. Introduction

Humans are able to detect and discriminate five basic taste qualities: sweet, umami, bitter, salty and sour [1]. Sweet taste has a central role in the detection of sugars, which are the main sources of readily available energy. Molecules other than sugars (glucose, fructose and sucrose) also exhibit a sweet taste, including natural (e.g., rebaudioside A (RebA), rebaudioside M (RebM) and mogroside V), semisynthetic (e.g., neohesperidin dihydrochalcone (NHDC) and perillartine) and synthetic (e.g., sucralose, neotame, acesulfame K (AceK), saccharin and cyclamate) sweeteners. Sweet and taste-modifying plant proteins, including thaumatin and brazzein, have also been identified [2]. Currently, several studies have shown that high sugar intake and sweet taste sensitivity may be linked to increased risks of several health disorders, including dental caries, obesity, type 2 diabetes, metabolic syndrome and cardiovascular disease [3,4,5].

The detection of sweet taste is mediated by a single heterodimeric class C G protein-coupled receptor (GPCR) composed of two subunits, TAS1R2/TAS1R3, in taste buds [6,7]. The subunit TAS1R3 is also involved in the detection of umami compounds and is composed of the heterodimeric umami taste receptor TAS1R1/TAS1R3 [6,8]. These three subunits share a common architecture consisting of a large extracellular domain (ECD) composed of a Venus flytrap domain (VFT) and a cysteine-rich domain (CRD) linked to the 7-helix transmembrane domain (7TM). The sweet taste receptor TAS1R2/TAS1R3 has been shown to have multiple binding sites for sweet taste compounds. TAS1R2-VFT is the main ligand-binding site for most sweet-tasting compounds, including natural sugars (sucrose, fructose and glucose) and numerous sweeteners (sucralose, aspartame, neotame, acesulfame K, saccharin and stevioside), whereas TAS1R2-7TM was shown to bind perillartine. The VFT domain of TAS1R3 interacts with sucrose, glucose and sucralose. In addition, TAS1R3-7TM contains a binding site for cyclamate and NHDC. Although the mechanism of receptor activation by sweet-tasting proteins is not completely understood, brazzein and thaumatin interact with the two CRDs of the TAS1R2/TAS1R3 receptor [9,10,11]. The presence of multiple binding sites explains the chemical diversity of sweet taste compounds [12,13,14,15,16] and the sweet-tasting synergism observed within sweetener blends [17].

Many researchers have demonstrated the involvement of specific amino acid residues in the binding of sweet compounds using cell-based assays combined with in silico molecular modelling (docking) and site-directed mutagenesis. For example, the substitutions of S144, E302 and D307 in TAS1R2-VFT abolish or significantly reduce human sweet taste receptor responses to aspartame and neotame [18,19]. The S40 amino acid residue of TAS1R2 has been shown to cooperate with D142 to facilitate spatial orientation for agonist binding and stabilise the closed conformation of activated TAS1R2/TAS1R3. In addition, residue I67 modulates the affinity of neotame in TAS1R2 [20]. Eleven critical residues (S40, Y103, D142, S144, S165, S168, Y215, D278, E302, D307 and R383) within and near the binding site of the TAS1R2-VFT pocket have been demonstrated to be important for ligand recognition and receptor activation by aspartame [21]. In addition, residues D142 and D307 have been shown to be crucial for the binding of aspartame, D-tryptophan, sucralose, saccharin, AceK and cyclamate to TAS1R2, as are Y103, P277, D278 and E302 for sucralose and amino-acid-derived sweeteners [22]. A total of six residues located in TAS1R3-7TM (Q636, H641, F778, L782, H721 and R723) are also required for cyclamate receptor responses [23]. Cell-based assays combined with site-directed mutagenesis have demonstrated that eight substitutions (Q637, H641, S640, H721, F730, W775, F778 and L782) abolish NHDC binding, some of which overlap with those involved in the cyclamate-binding site [24]. The alanine substitution of residues in TAS1R3-7TM revealed that seven key residues (A733, L798, R790, S640, H641, F778 and L782) are required for sensitivity to lactisole, a broad-acting sweet inhibitor [25]. TAS1R3-CRD is also involved in the interaction of some sweet-tasting proteins as their large size prevents them from accessing the orthosteric binding site of VFTs in TAS1R2 and TAS1R3 [11,26]. Specifically, the residues A537 and F540 located in the CRD are important for TAS1R3 responses to brazzein [9]. In addition, cell membranes expressing the sweet taste receptor with a D535Q mutant of TAS1R3-CRD show a deficit of specific activation with brazzein [10]. Human TAS1R3-CRD is required for the interaction between the human sweet taste receptor and thaumatin [27]. Finally, five critical residues (Q504K, A537T, R556P, S559P and R560K substitutions) dispersed in the CRD of human TAS1R3 are involved in the response to thaumatin [11].

The functionality of taste receptors can be affected by polymorphisms in *TAS1R* (umami and sweet tastes) and *TAS2R* (bitter taste) genes. Most of the genetic variations observed are single-nucleotide substitutions, known as single-nucleotide polymorphisms (SNPs). A few genetic studies on taste receptors have linked genetic variation in bitter, umami and sweet taste receptors to chronic disease risk [28,29,30,31,32,33,34,35,36,37,38]. In addition, the presence of taste receptors in many extraoral tissues raises the question of their role, particularly in regulating metabolism [39]. Taste receptor genetic modifications could, therefore, lead to differences in taste detection, resulting in differences in food choices, preferences, habits and food intake [40], which could affect metabolism and, therefore, health [41]. For example, two SNPs have been identified in the bitter taste receptor *TAS2R38* gene: PAV (proline–alanine–valine) and AVI (alanine–valine–isoleucine). Cellular studies have demonstrated that TAS2R38-AVI abolishes the receptor functionality stimulated by phenylthiocarbamide (PTC) and propylthiouracil (PROP), whereas TAS2R38-PAV has the highest functionality. Compared with AVI/PAV and AVI/AVI subjects, PAV/PAV subjects exhibited greater bitter taste intensity [42]. In addition, the substitution of residues F749S and R757C in TAS1R3-7TM severely alters the in vitro TAS1R1/TAS1R3 response to monosodium glutamate and in vivo taste recognition thresholds [43,44,45]. With regard to the sweet taste receptor, some allelic polymorphisms in the promoter of the *TAS1R3* gene are strongly correlated with a reduction in sucrose perception in humans [46,47]. However, these results are not linked to the functionality of the receptor as these substitutions are located in a noncoding region. A study on the polymorphisms of three human *TAS1R* genes from 88 individuals originating from eight geographically different regions revealed that the *TAS1R3* gene shows less variation than do the *TAS1R1* and *TAS1R2* genes. Moreover, four substitutions in TAS1R2-VFT close to the main binding site for sweet compounds could be responsible for interindividual differences in sensitivity to sweet molecules [48]. The I191V variant of *TAS1R2* has been associated with an increase in sugar intake in obese and overweight individuals [49]. Obese adolescents carrying the serine allele of the SNP rs9701796 in *TAS1R2* (S9C variant) have a higher waist-to-height ratio and a higher consumption of chocolate powder, whereas those carrying the valine allele of the SNP rs35874116 in *TAS1R2* (I191V variant) have lower dietary fibre intake [50]. The SNP rs12033832 variant in *TAS1R2* is associated with both sweet taste perception and sugar consumption in a body mass index (BMI) dependent manner. Indeed, individuals with an SNP rs12033832 variant in *TAS1R2* and a BMI ≥ 25 kg/m^2^ have lower sensitivity ratings and consume more sugars, whereas individuals with a BMI < 25 kg/m^2^ have lower thresholds and consume fewer sugars [51]. Other studies, which have not been carried out on an obese population, have shown that people with this mutation have a higher consumption of sweet foods and a greater perception of sweet taste [52,53,54]. Fewer studies have investigated the impact of the *TAS1R3* polymorphism on sweet taste detection than have investigated the *TAS1R2* polymorphism. However, a few studies have demonstrated in mice a link between a preference for some sugars and a loss of function of the sweet taste receptor. Indeed, in mice, polymorphisms in the taste receptor gene (*Tas1r3*) are associated with a preference for saccharin. An in vitro study of glucose/sucrose/sucralose affinity to the mTAS1R3-VTF domain also showed that the ligand affinity for this variant is lower than that of m-TAS1R3-WT (wild type) [12].

To fill the gaps in the verification of in vivo and in vitro correlations, the aim of this study was to examine the impact of human *TAS1R2*/*TAS1R3* polymorphisms (located in the coding sequences) identified in a literature review and associated with obesity and/or high sugar intake on the functionality of the sweet taste receptor stimulated by 12 different sweeteners selected for the SWEET project. First, the effects of mTAS1R3-WT and mTAS1R3-I60T on the functionality of the sweet taste receptor were compared as the I60T SNP was associated with a preference for saccharin in mice. Indeed, it is the only *TAS1R2*/*TAS1R3* SNP for which in vivo and in vitro studies have been carried out, demonstrating a correlation between a reduced receptor functionality and a preference for some sweet-tasting compounds. Second, the effects of 12 *TAS1R2*-SNPs and 16 *TAS1R3*-SNPs were investigated using an in vitro functional cell-based assays. Third, the TaqMan method for SNP genotyping was carried out using DNA extracted from blood samples collected from an obese population (from the SWEET project) to identify new SNPs associated with a loss of function of TAS1R2/TAS1R3.

## 2. Materials and Methods

### 2.1. The SWEET Project

The SWEET project is funded by the European Commission Horizon 2020 (https://sweetproject.eu/, accessed on 15 April 2024). It aims to develop and review evidence on the long-term benefits and potential risks involved in switching to sweeteners and sweet enhancers in the contexts of public health and safety, obesity and sustainability. This 5-year multidisciplinary project involves stakeholders from across the food chain (consumers, patients, health professionals, scientists, policy-makers and regulators). The current study was conducted as a part of a work package (WP2—Short-Term Impact on Food Behaviour, Physiology and Health) that aims to characterise new and emerging sweeteners, identify suitable candidates to form innovative blends and develop newly reformulated sugar-reduced food products, including individual sweetener and/or sweetener blends [17].

### 2.2. Sweeteners

**Figure 1:**
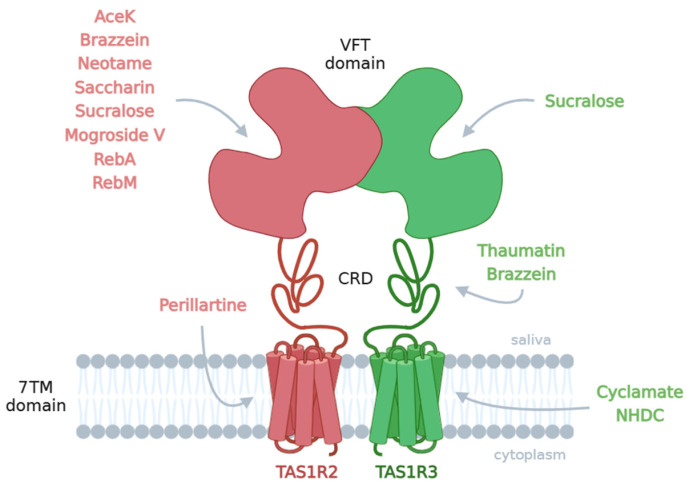
Schematic summary of the different TAS1R2/TAS1R3 binding sites of the 12 studied sweeteners [9,11,12,13,14,15,16,19,56,57]. AceK: acesulfame-K; RebA: rebaudioside A; RebM: rebaudioside M; NHDC: neohesperidin dihydrochalcone; VFT: Venus flytrap domain; CRD: cysteine-rich domain; 7TM: 7-helix transmembrane domain.

A total of 12 sweeteners were selected by the 27 European partners of the project and had to respond to various criteria, including diversity of their origins (natural, synthetic, plant-based, etc.), sustainability, heat resistance to be incorporated into different matrices (beverages, biscuits, yogurt, chocolate and breakfast cereals) and use in innovative blends to reduce the number of sweet-tasting compounds while exhibiting the same sweetness intensity [17]. Moreover, they were also selected for their ability to bind to at least one of the multiple binding sites described for the TAS1R2 or TAS1R3 subunits of the sweet taste receptor. A summary of the taste receptor binding sites of the tested sweeteners is shown in Figure 1. Unfortunately, common sugars such as sucrose were not tested as their sweet potency and affinity for the sweet taste receptor are very low. Indeed, the high concentrations of sugars required to measure the in vitro functional response of cells affect the physicochemical properties of the buffer by increasing the osmolarity of the environment, leading to osmotic changes between the extracellular buffer and the intracellular cell cytoplasm and resulting in nonspecific response to cells expressing the TAS1R2/TAS1R3 receptor.

Sucralose (E955, synthetic, 99.6%), AceK (E950, synthetic, ≥99%), saccharin (E954, synthetic, ≥99%), cyclamate (E952, synthetic, ≥99%), NHDC (E959, semisynthetic, ≥99%) and thaumatin (E957, natural, ≥99%) were purchased from Sigma-Aldrich (Merck Group; Saint-Quentin-Fallavier, France). RebA (Truvia^®^ Stevia Leaf Extract; E960, natural, ≥95%) and RebM (Truvia^®^ Stevia Leaf Extract; E960, natural, ≥80%) were kindly provided by Cargill (Cargill R&D Centre Europe BV; Vilvoorde, Belgium). Neotame (E961, synthetic, ≥99%) was purchased from NutraSweet (Augusta, GA, USA), mogroside V (natural, 87.1%) was purchased from ChromaDex (Longmont, CO, USA) and perillartine (semisynthetic, 95%) was purchased from Combi-Blocks (San Diego, CA, USA). The sweet-tasting protein brazzein was obtained by recombinant production using the yeast *Pichia pastoris* in our laboratory [55]. Mogroside V is still awaiting EFSA (European Food Safety Authority) approval, whereas brazzein is not approved by the EFSA.

### 2.3. In Vitro Cell-Based Assays Studying Human TAS1R2- and TAS1R3-SNPs

#### 2.3.1. Selection and Expression of *TAS1R2*- and *TAS1R3*-SNPs

The choice of SNPs used for the cellular assays was based on a literature review. We selected the most frequently cited SNP reference sequence that resulted in an amino acid residue change (nonsynonymous SNP) in the final protein sequence. The selected SNPs of *TAS1R2* and *TAS1R3* are presented in Table 1. The DNA and amino acid sequences used for hTAS1R2-WT and hTAS1R3-WT were found on UniProtKB (https://www.uniprot.org/uniprot/, accessed on 1 September 2019) with reference number Q8TE23 (NCBI reference sequence NM_152232. 5) with Cys at position 9 and Q7RTX0 with Cys at position 757, respectively, which both corresponded to the most frequent position allele (Supplementary Materials).

The coding sequences for TAS1R2 and TAS1R3 were commercially synthesised, and the human codon was optimised for expression in human cells. The coding sequences of human TAS1R2 and TAS1R3 were subcloned and inserted into the mammalian expression vectors pcDNA6/myc-HisA and pcDNA4/myc-HisA (Invitrogen; Thermo Fisher Scientific; Illkirch, France) between the *Eco*RI and *Not*I restriction sites, generating the plasmids pcDNA6-TAS1R2 and pcDNA4-TAS1R3, respectively. To increase protein expression in mammalian cells, an upstream start codon was used, which was a sequence called MAX, corresponding to the QBI SP163 element also described in pcDNA4-HisMax (Invitrogen; Thermo Fisher Scientific; Illkirch, France) [64]. To measure the expression level of the receptor by immunohistochemistry, we used the FLAG sequence (DYKDDDDK) in the C-terminal position, which encodes the FLAG tag epitope [64]. SNP variants of *TAS1R2* and *TAS1R3* were constructed based on pcDNA4-MAX-TAS1R2-FLAG and pcDNA6-MAX-TAS1R3-FLAG, respectively, using site-directed mutagenesis (Genewiz; Leipzig, Germany).

All the expression plasmids (5 µg) were diluted in 200 µL of water and transformed into *E. coli* Top10 F’-competent bacteria using the heat shock method. Vectors were amplified by bacterial culture in Luria Broth medium with ampicillin antibiotic selection. Plasmid DNA extraction was performed using the QIAfilter Plasmid Midi Kit (Qiagen; Courtabœuf, France) following the manufacturer’s instructions. The sequences of all plasmid constructs were verified by automated DNA sequencing (Genewiz; Leipzig, Germany). As previously described [64,65], cell immunocytochemistry experiments were also performed to confirm the level of expression of hTAS1R2-FLAG and hTAS1R3-FLAG in all constructs (WT and variants).

#### 2.3.2. Calcium Mobilisation Assay

The HEK293T cells were kindly provided by Jay P. Slack (Givaudan Flavors Corporation; Cincinnati, OH, USA) [66]. HEK293T cells stably transfected with Gα16gust44 [43,67] were seeded in poly-D-lysine-coated clear-bottom black 96-well plates (0.35 × 10^5^ cells/well) in high-glucose DMEM supplemented with 2 mM GlutaMAX, 10% dialysed foetal bovine serum with penicillin/streptomycin and G418 (400 μg/mL) at 37 °C and 6.3% CO_2_ in a humidified atmosphere as previously reported [16,17]. Twenty-four hours later, using Lipofectamine 2000 (0.4 μL/well; Invitrogen), the cells were transiently transfected with pcDNA6-TAS1R2 (60 ng/well), pcDNA4-TAS1R3 (60 ng/well) and pCMV-GCaMP5G (Addgene #31788; 50 ng/well), which encodes the calcium biosensor. As a negative control, HEK293T cells were mock-transfected with the empty expression vector. After a further 24 h of incubation, the HEK293T cells were washed with C1 buffer (130 mM NaCl, 5 mM KCl, 10 mM HEPES pH 7.4 and 2 mM CaCl_2_). The 96-well plates containing the cells were then stimulated with sweet-tasting compounds. The fluorescence intensity was measured for 90 s (excitation 488 nm, emission 510 nm) in an automated fluorometric FlexStation^®^3 Multi-Mode microplate reader (Molecular Devices; San Jose, CA, USA). Different concentration ranges were tested depending on the sweet compound injected. The Ca^2+^ changes were expressed as fractional changes in fluorescent light intensity: ΔF/F = (F − F_0_)/F_0_, where F was the fluorescent light intensity at each point, and F_0_ was the value of emitted fluorescent light prior to stimulus application. To calculate dose–response relationships, the changes in fluorescence upon stimulus application were averaged, mock-subtracted and baseline-corrected. The dose–response data obtained were adjusted using a 4-parameter logistic equation. The half-maximal effective concentrations (EC_50_ values) and maximal signal amplitudes were calculated using SigmaPlot software (version 15.0) (Systat Software; San Jose, CA, USA).

### 2.4. In Vitro Cell-Based Assays for Studying Mouse TAS1R3

HEK293T-Gα16gust44 cells were transiently cotransfected with pcDNA6-MAX-mTAS1R2-FLAG, pcDNA4-MAX-mTAS1R3-WT-FLAG or pcDNA4-MAX-mTAS1R3-I60T-FLAG, and pCMV-GCaMP5G as previously described in detail. After 24 h, the cells were washed with C1 buffer and stimulated with a logarithmically increasing range of sweetener concentrations. Calcium responses during automated application were monitored using a FlexStation^®^3 Multi-Mode microplate reader. The dose–response curves, EC_50_ values and maximal signal amplitudes were calculated as previously described.

### 2.5. Statistical Analysis

For in vitro calcium mobilisation assay performed on hTAS1R2-SNPs, hTAS1R3-SNPs and mTAS1R3-I60T, 6 to 7 concentrations were tested for each sweet compound, plus buffer alone. All concentration–receptor combinations were measured in duplicate, and each experiment was repeated at least 4 times (8 wells/concentration). Results were expressed for each concentration as ΔF/F mean ± SEM and represented as dose–response curves. Statistical data analysis (one-way analysis of variance (ANOVA) with Dunnett’s test) was performed using XLSTAT 2023 (Addinsoft, Paris, France) to compare the difference between the ΔF/F mean of variant and the ΔF/F mean of WT for each concentration. *p*-Values < 0.05 were considered statistically significant.

### 2.6. In Vivo SNP Genotyping Assays

#### 2.6.1. Subjects

The subjects were recruited from 5 centres (Table 2) involved in WP2 of the SWEET project, which consisted of a double-blind randomised crossover trial with 3 product formulations (a sucrose control and 2 individual sweeteners or binary blends) over 5 intervention product types (beverages, biscuits, yogurt, chocolate and breakfast cereal matrices). Males and females (18 to 60 years) with a BMI between 25 and 35 kg/m^2^ were eligible. Several analyses were carried out on the participants, particularly a blood test. The blood samples used in this study were collected on the first clinical investigation day (CID) of each participant. Each whole blood sample was stored in a 2.5 mL PAXgene^®^ Blood DNA tube (BD vacutainer 761115, BD Biosciences; Le Pont de Claix, France) treated with K_2_EDTA and BD Hemogard™ (BD Biosciences). This tube is specially designed for collecting, storing and transporting a blood sample to prepare DNA for molecular diagnostic tests. The addition of K_2_EDTA stabilises the DNA during sampling. The blood samples were then frozen within 1 h of collection at −70 or −80 °C and then sent, in packaging containing dry ice, to our laboratory without being associated with the results obtained during the WP2 analysis. All 5 centres obtained ethical approval from their local ethical committees: Comité de Protection des Personnes Nord-Ouest III (2021-42, approved 28 March 2022), Comité de Ética de la Investigación de la Universidad de Navarra (2021.205, approved 7 March 2022), Ethical Committee Region H Denmark (H-21078447, approved 27 September 2022), University of Liverpool Central University Research Ethics Committee D (10659, approved 14 April 2022) and University of Leeds School of Psychology (PSC-127, approved 19 November 2020). The trial was conducted in accordance with the Declaration of Helsinki (registration numbers NCT04483180 and NCT04633681). See [68,69] for more details.

A total of 162 blood samples distributed across the 5 centres were collected in our laboratory (Table 2) and then shipped in packaging containing dry ice to IntegraGen SA (Evry, France) for polymorphism analyses. For the polymorphism study, we obtained the authorisation for the “conservation and preparation for scientific purposes of elements of the human body for the needs of research programs” by the Bioethics Unit of the French Ministry of Research (DC-2020-3928, approved June 2021).

#### 2.6.2. Blood Sample Polymorphism Analysis

Library preparation, sequence capture, sequencing and data analysis were performed by IntegraGen SA.

The following methods were adapted from a previous study [70]. Sequence capture, enrichment and elution were carried out according to the manufacturer’s instructions and protocols (Twist Bioscience; San Francisco, CA, USA) without modification, except library preparation using the NEBNext^®^ Ultra II kit (New England Biolabs^®^; Evry, France). For library preparation, 150 ng of each genomic DNA sample was fragmented by sonication and purified to yield 150–200 bp fragments. Paired-end adapter oligonucleotides from the NEB kit (NEBNext^®^) were ligated on repaired, a-tailed fragments and then purified and enriched by 7 cycles of PCR (polymerase chain reaction). Five hundred nanograms of these purified libraries was then hybridised to the Twist oligo probe capture library for 16 h in a single-plex reaction. After hybridisation, washing and elution, the eluted fraction was amplified by PCR over 8 cycles, purified and quantified by QPCR (quantitative-PCR) to obtain sufficient DNA template for downstream applications. Each eluted-enriched DNA sample was then sequenced on an Illumina NovaSeq system (Evry, France) as a paired-end 100 reads. Image analysis and base calling were performed using Illumina Real Time Analysis software (version 3.4.4) with the default settings.

For TAS1R2/TAS1R3 variant analysis (sequence alignment and variant calling), base calling was performed using the Real Time Analysis software sequence pipeline (Illumina; version 2.7.7) with default settings. Raw human reads were aligned to the hg38 human genome using the Burrows–Wheeler Aligner (BWA) Tool 1. Duplicated reads were removed using Sambamba2. Somatic single-nucleotide variants (SNVs) and small insertions/deletions (indels) were generated via GATK3,4 Haplotype Caller GVCF software (Broad Institute; Cambridge, MA, USA; version 4.1.2.0). The VQSR method was applied, and low-quality variants were removed using hard filters (“QD < 2.0 || FS > 60.0 || MQ < 40.0 || MQRankSum < −12. 5 || ReadPosRankSum < −8. 0 || ExcessHet > 54.69 | GQ < 20.0” for SNPs and “QD < 2.0 || FS > 200.0 || ReadPosRankSum < −20.0 || ExcessHet > 54.69 || QUAL < 30.0 || GQ < 20.0” for indels).

Ensembl’s Variant Effect Predictor5 (Cambridge, UK; version 101) was used to annotate variants according to their functional consequences (type of mutation and prediction of the functional impact on the protein by PolyPhen 2.2.2.2. (http://genetics.bwh.harvard.edu/pph2/, accessed on 10 August 2023) [71]) and their frequency in public databases (dbSNP146, 1000 Genomes phase 3, gnomAD genomes r3.0, and COSMIC v91) and internal databases. The variants were classified by tiers as follows: Tiers 1, 2 and 3 corresponded to truncating variants (nonsense, splice and frameshift), missense variants predicted to be damaging (by PolyPhen 2.2.2.2.) and other missense variants, respectively.

## 3. Results

### 3.1. Response of Mouse TAS1R3-I60T Variant to Sweeteners

**Figure 2:**
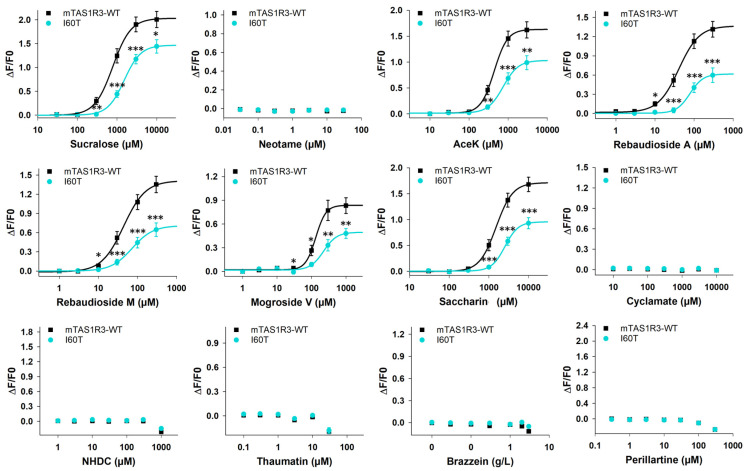
mTAS1R2-WT/mTAS1R3-WT (black line) and mTAS1R2-WT/m-TAS1R3-I60T (turquoise line) dose–response curves with 12 sweeteners. The data are presented as the mean ± SEM of 8 wells from 4 independent experiments. * *p* < 0.05, ** *p* < 0.01, *** *p* < 0.001, calculated using ANOVA followed by Dunnett’s test (with reference to mTAS1R2-WT/mTAS1R3-WT). The *p*-values are presented in Table S1. WT: wild type; AceK: acesulfame-K; NHDC: neohesperidin dihydrochalcone.

A cell-based assay was carried out to determine the dose–response relationships for mTAS1R3-WT and mTAS1R3-I60T coexpressed with mTAS1R2-WT after stimulation with the 12 studied sweeteners (Figure 2). The EC_50_ values and the maximal signal amplitudes of mTAS1R2-WT/mTAS1R3-WT and mTAS1R2-WT/mTAS1R3-I60T were determined (Table 3). As expected, the mouse sweet taste receptor was not activated by neotame, cyclamate, NHDC, thaumatin, brazzein or perillartine. For all other sweeteners, the I60T substitution in mTAS1R3 led to a significant decrease in the overall functional activity of the sweet taste receptor, which was characterised by a shift in the dose–response curve to the right, leading to an increase in the EC_50_ value accompanied by a decrease of more than 40% in the maximal amplitude of the signal.

### 3.2. Response of Human TAS1R2 and TAS1R3 Variants to Sweeteners

#### 3.2.1. TAS1R2 Variants

**Figure 3:**
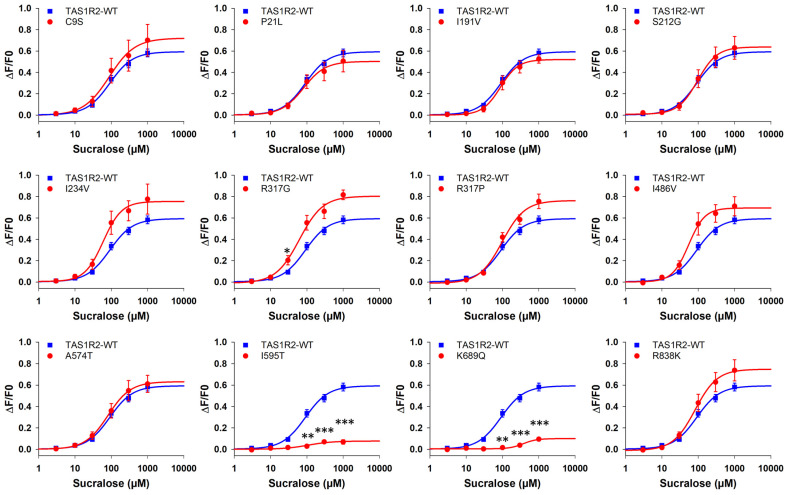
Human TAS1R2-WT/TAS1R3-WT (blue line) and TAS1R2-SNP/TAS1R3-WT (red line) dose–response curves with sucralose. HEK293T-Gα16gust44 cells were transiently transfected with pcDNA6-MAX-TAS1R2-WT-FLAG or pcDNA6-MAX-TAS1R2-SNP-FLAG and pcDNA4-MAX-TAS1R3-WT-FLAG. The data are presented as the mean ± SEM of 8 wells from 4 independent experiments. * *p* < 0.05, ** *p* < 0.01, *** *p* < 0.001, calculated using ANOVA followed by Dunnett’s test for multiple comparison analysis (with reference to TAS1R2-WT/TAS1R3-WT). The *p*-values are presented in Table S2. WT: wild type.

A cell-based assay was carried out to determine dose–response relationships for the WT and 12 variants of the TAS1R2 subunit, which were coexpressed with TAS1R3-WT and stimulated with 12 sweeteners (Figure 3 for sucralose as an example and Figure S1A–L for the 11 other sweeteners). The dose–response curves are also presented as a function of each SNP in Figure S2A–L. For each variant–sweetener combination, the EC_50_ value and the maximal signal amplitude were determined and compared with those of TAS1R2-WT/TAS1R3-WT (Table 4). Cell-based immunocytochemistry experiments confirmed expression of hTAS1R2-WT and hTAS1R2-SNPs (Figure S3).

In general, little variation was observed in the cellular response of the receptor variants compared with that of the WT. We found that all the variants, except the I595T and K689Q variants, had similar responses to TAS1R2-WT for most of the tested sweeteners. Indeed, we observed that the I595T and K689Q substitutions severely impaired the ability to respond to most of the tested sweeteners. Both variants exhibited a shift to the right of the dose–response curves and an important decrease in the maximal signal amplitudes for all the sweeteners, indicating a decrease in potency and efficacy, respectively. Compared with that of the TAS1R2-WT, the maximal signal amplitude was lower for the I191V variant when it was stimulated by saccharin. The variant R317P exhibited higher EC_50_ values and lower maximal signal amplitudes than TAS1R2-WT when it was stimulated by the two sweet-tasting proteins, thaumatin and brazzein. Interestingly, we observed for perillartine that all the substitutions of TAS1R2 (single subunit) resulted in a strong decrease in both potency and efficacy. Although the monomeric TAS1R2 subunit can be activated by perillartine alone [57,65], the response was always higher for the TAS1R2/TAS1R3 heterodimer than for the TAS1R2 subunit alone. Finally, a decrease in the EC_50_ values was observed for the R317G variant (EC_50_ = 62 ± 11 µM) stimulated by sucralose compared with TAS1R2-WT (EC_50_ = 90 ± 11 µM) and saccharin (EC_50_ = 224.5 ± 6.1 µM for the R317G variant and EC_50_ = 274.4 ± 4.1 µM for TAS1R2-WT).

#### 3.2.2. TAS1R3 Variants

**Figure 4:**
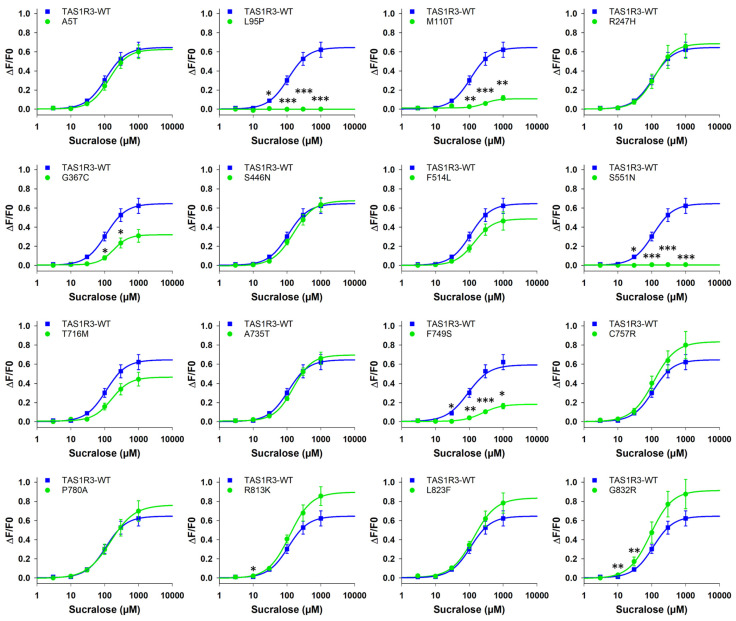
Human TAS1R2-WT/TAS1R3-WT (blue line) and TAS1R2-WT/TAS1R3-SNP (green line) dose–response curves with sucralose. HEK293T-Gα16gust44 cells were transiently transfected with pcDNA6-MAX-TAS1R2-WT-FLAG and pcDNA4-MAX-TAS1R3-WT-FLAG or pcDNA4-MAX-TAS1R3-SNP-FLAG. The data are presented as the mean ± SEM of 8 wells from 4 independent experiments. * *p* < 0.05, ** *p* < 0.01, *** *p* < 0.001, calculated using ANOVA followed by Dunnett’s test for multiple comparison analysis (with reference to TAS1R2-WT/TAS1R3-WT). The *p*-values are presented in Table S3. WT: wild type.

The same approach was carried out on 16 variants of the TAS1R3 subunit coexpressed with TAS1R2-WT and stimulated by 12 sweeteners (Figure 4 for sucralose as an example and Figure S4A–K for the 11 other sweeteners). The dose–response curves are also presented as a function of each SNP in Figure S5A–P. The EC_50_ value and the maximal signal amplitude for each variant–sweetener combination were measured and compared with those of TAS1R2/TAS1R3-WT (Table 5). Cell-based immunocytochemistry experiments confirmed the expression of hTAS1R3-WT and hTAS1R3-SNPs (Figure S6).

The A5T, R247H, S446N, F514L and T716M variants showed a similar response to TAS1R3-WT for all sweeteners (except RebA for the T716M variant and brazzein for the F514L variant). However, five TAS1R3 substitutions, including L95P, M110T, G367C, S551N and F749S, led to a decrease in the activity of the sweet taste receptor for most of the tested sweeteners. In particular, the L95P and S551N substitutions completely abolished or decreased the receptor response to low-molecular sweeteners. Interestingly, the other variants (except the L95P, M110T and S551N variants) exhibited similar responses to TAS1R3-WT when stimulated with perillartine. As perillartine binds to TAS1R2-7TM [57], these mutations should not affect the function of the receptor stimulated by this sweetener.

Interestingly, the T716M, A735T, C757R, P780A, R813K, L823F and G832R variants presented higher potencies and/or efficacies than did TAS1R2-WT for some sweeteners, particularly RebA, RebM and mogroside V. A decrease in the EC_50_ values was observed for the G832R variant stimulated with sucralose, neotame, saccharin and cyclamate and for the R813K variant stimulated with saccharin. The A735T, C7557R, R813K, L823F and G832R variants presented higher functionality than did TAS1R3-WT when stimulated with AceK. The P780A variant exhibited higher maximal signal amplitudes than did TAS1R3-WT when stimulated with NHDC. Interestingly, the variants A735T, P780A, R813K and G832R exhibited lower EC_50_ values for the thaumatin than did TAS1R3-WT. In addition, the EC_50_ value was lower for the R813K variant stimulated by brazzein, the other sweet-tasting protein, than was TAS1R3-WT. Finally, the R813K and G832R variants showed a higher response than did TAS1R3-WT for at least seven of the tested sweeteners. For example, G832R showed lower EC_50_ values and/or higher maximal signal amplitudes when the receptor was stimulated with sucralose, neotame, AceK, RebA, RebM, mogroside V, saccharin, cyclamate, thaumatin or brazzein.

### 3.3. Results of TAS1R2/TAS1R3 SNP Sequencing in an Obese Population

The results of the SNPs detected for the *TAS1R2* and *TAS1R3* genes from the blood analysis of participants with a BMI between 25 and 35 kg/m^2^ are presented in Table 6. A total of 26 and 25 alleles were detected for the *TAS1R2* and *TAS1R3* genes, respectively. Interestingly, 7 TAS1R2 variants (I595T, P21L, R838K, A574T, I486V, R317G and I191V) over 12 and 4 TAS1R3 variants (A735T, R247H, A5T and C757R) over 16 previously studied by cellular assays were also detected in the DNA sequencing analysis. The TAS1R2 and TAS1R3 variants studied in vitro but not identified in the tested population had frequencies of <1%, which may explain their absence in this cohort compared with the general population. Surprisingly, the following variants were not identified in the studied population even though they presented higher frequencies: 20.2 and 1.6% for C9S (*TAS1R2*) and G367C (*TAS1R3*), respectively. Overall, the studied population had a frequency of SNPs similar to that identified in gnomAD, particularly for the P21P, P704P, I790I, T294T, F77F, S773S, M249I, I486V, R317G, I191V and S9C TAS1R2 variants and for the A579A, G839G, K77K, R479R and C757R TAS1R3 variants. Although there were differences in frequency between our study and that of gnomAD, the frequencies of these identified SNPs remained quite low in the general population (<5%), except for the A5A TAS1R1 variant (6.5% in our study compared with 4.5% in the general population) and for the P416P TAS1R3 variant (6.8% in our study compared with 12.2% in the general population). Moreover, some SNPs had a frequency of 0.0031, which corresponded to the identification of a single allele in our cohort; this frequency may be overestimated due to the small number of participants compared with the general population. These differences in frequency between the tested and general populations suggest that our cohort was not representative, given the low number of participants. Finally, K75Q and S372S for TAS1R2 variants and A5T (altered nucleotide: ACA) for the TAS1R3 variant were detected in the studied population but not in the general population.

The DNA sequencing analysis identified 15 SNPs for *TAS1R2* and 10 SNPs for *TAS1R3* with a moderate impact on subunit functionality, of which only 4 and 3 SNPs, respectively, are possibly or probably responsible for less or no function of the subunit and/or the sweet taste receptor. Although the W668 substitution had a strong effect on the functionality of TAS1R3, no possible or probable loss of function was predicted.

## 4. Discussion

### 4.1. Is There a Clear Link Between Certain Phenotypes (Higher Sugar Intake and/or Higher Detection Threshold) and the Cellular Response of Associated SNPs?

The aim of this study was to verify whether certain SNP variants previously associated with particular phenotypes were responsible for a loss of receptor functionality when stimulated by different sweeteners. Cell-based assays were carried out on two mouse *TAS1R3* SNPs (including mTAS1R3-WT), 13 human *TAS1R2* SNPs (including TAS1R2-WT) and 17 human *TAS1R3* SNPs (including TAS1R3-WT) stimulated with 12 sweeteners (Table 3, Table 4 and Table 5, respectively).

As expected, the mouse sweet taste receptor was not activated by neotame, cyclamate, NHDC, thaumatin, brazzein or perillartine, which are not palatable to mice [72]. For all other sweeteners, the mTAS1R3-I60T variant showed a decrease in EC_50_ values and reduced maximal amplitudes compared with those of the WT. Our data were in agreement with the results of binding experiments showing a reduced affinity of TAS1R3-I60T for glucose, sucrose and sucralose compared with TAS1R3-WT [12]. Given that the TAS1R3-I60T sequence variant has been linked to a preference for saccharin [73], our results suggest that this polymorphism (in vivo data) is associated with a decrease in sweet taste receptor functionality (in vitro data).

With respect to hTAS1R2, 10 over the 12 variants studied showed no difference in response to TAS1R2-WT for most of the sweeteners. In addition, the I595T and K689Q variants exhibited a decrease in efficacy and potency for all the tested sweeteners, suggesting a structural impact on overall receptor function. Among the 12 TAS1R2 missense SNPs that were studied based on the literature review, 9 had a recorded frequency > 1% in the population (Table 1) and were located in the VFT domain. In previous studies, the I191V variant was strongly associated with sugar intake [49,61]. For example, sugar intake is lower in individuals carrying the I191V variant and presenting a BMI of 25 kg/m^2^ [49], and children (Val/Val) ingest less sugar and sugar-dense foods than do children who are Ile carriers [61]. Another study showed that Val/Val genotype carriers had a higher intake of total carbohydrates, fibre and servings of cereals/vegetables [62]. Our cellular results did not reveal an association between receptor functionality and eating behaviour as the substitution I191V did not impact receptor activation by different sweeteners (except for saccharin). Our data were in line with another study that showed that despite the association of these SNPs with sugar consumption, none were linked to changes in sweet taste perception [74]. In addition, a previous study showed that the I191V polymorphism was not related to the risk of obesity [75]. Therefore, to determine a potential correlation, it would be useful to check whether individuals with the other studied variants exhibited differences in the sweet detection threshold of these sweeteners and in their sugar intake.

Our functional data on *TAS1R3*-SNPs demonstrated that human *TAS1R3* polymorphisms led to a greater variation in receptor functionality than previously expected as the *TAS1R2*-SNP was described as the main binding site for sweet compounds. Our functional data demonstrated that a total of seven substitutions in human TAS1R3 improved the efficacy/potency of the sweet taste receptor. For example, the R813K and G832R variants showed the highest responses compared with TAS1R3-WT for most of the sweeteners. The 16 missense variants studied in vitro were distributed throughout the TAS1R3 subunit of the sweet taste receptor, and 5 were associated with a decrease in functionality (L95P, M110T, G367C, S551N and F749S). The G367C SNP was of interest because it presented a recorded frequency > 1% in the population. L95P and S551N variants, which are very weakly expressed in the overall population, strongly abolished the receptor functionality for 12 sweeteners. The M110T, G367C and F749S substitutions induced a decrease in the response to most of the sweeteners, suggesting a structural impact on overall receptor function. Interestingly, the decrease in the receptor functionality of the F749S variant was consistent with another study showing that this substitution severely impaired the TAS1R1/TAS1R3 in vitro response to monosodium glutamate [43]. Surprisingly, the C757R substitution located in the 7TM, which has also been associated with changes in umami detection [43,63], appeared to slightly improve the receptor functionality stimulated by AceK, RebA, RebM and mogroside V. It would be useful to check whether people with these variants exhibited differences in the sweet detection threshold of these sweeteners and in their sugar intake. Although we studied variants that had mainly been described for their sensitivity to umami compounds (in vivo data) [44,45], our results also raised the question of the effect of these *TAS1R3* SNPs, which modify the sweet taste receptor TAS1R2/TAS1R3 activity, on the functionality of the umami taste receptor (TAS1R1/TAS1R3) (in vitro data).

By homology, our results with mTAS1R2/TAS1R3 could be applied to our data observed for in vitro tests in human sweet taste receptors and extrapolated to the sensitivity of individuals carrying these mutations. Thus, a reduction in the amplitude of the signal in vitro and an increase in the EC_50_ value should be associated with a lower sensitivity in vivo for sweet-tasting molecules and explain the origin of a higher consumption of sugars. Although recent studies have investigated whether human taste receptor genes are associated with measured changes in taste sensitivity and preference [40], our data reinforce the hypothesis that sweet taste perception and sugar consumption are also governed by different receptor-mediated factors, including environmental, behavioural and other genetic factors [76]. Indeed, genome-wide meta-analyses of data from large-scale biobanks from different countries, or even smaller studies, revealed strong or suggestive associations between sweet taste preference, liking sweet, total sugar intake, and polymorphisms of genes other than *TAS1R2* and *TAS1R3* [74,77,78,79,80,81,82,83,84,85,86].

### 4.2. Possible/Probable SNPs of TAS1R2/TAS1R3 Associated with a Loss of Function of the Sweet Taste Receptor

The sequencing analysis (Table 6) revealed that certain SNPs, including K750Q (*TAS1R2*), S372F (*TAS1R2*) and A5T (altered nucleotides: ACA; *TAS1R3*), were identified only in the cohort and not in the general population, suggesting that they were more related to obese people. However, these results should be taken with caution as the frequency measured in our study for these SNPs corresponded to the allele of a single individual out of 162, and this genotyping assay should be carried out in a much larger population of obese people to confirm this prevalence. Nevertheless, a total of seven possible or probable SNPs with an impact on sweet taste receptor subunit functionality were identified (*TAS1R2*: K750Q, G523D, S372F and T314K; *TAS1R3*: W72G, R94C and D491H). These variants exhibited a very low frequency in the general population (<0.1%) but were overrepresented in the studied population (0.3%), which raised the question of the involvement of this mutation in obesity. It would be, therefore, relevant to repeat this genotyping study on a larger population to identify whether certain SNPs were more represented in an obese population. Moreover, none of these seven SNPs had been identified in the literature as having a link with obesity or a reduction in the functionality of the sweet taste receptor. It would be interesting to determine whether people carrying these alleles consumed more sugar and/or were less sensitive to sweet-tasting compounds using sensory analyses. It would also be possible to verify whether there are sensory and nutritional intake differences between mutation carriers of the *TAS1R2* gene and the *TAS1R3* gene to determine whether one of these genes is predominantly involved in obesity. Finally, it would be relevant to determine whether certain participants presented both mutations to verify whether the differences in sensitivity to sweet-tasting compounds and sugar intake were greater than those in carriers of only one of the two mutations. Moreover, these highlighted variants could also be studied using in vitro cell assays to confirm any decrease in or absence of response. Surprisingly, variants that showed little or no response when stimulated with sweeteners in previous trials or our cell-based assays, such as K689Q for *TAS1R2* and L95P, M110T, G367C, S551N and F749S for *TAS1R3*, were not identified in the studied population. This was probably explained by their very low frequency in the general population. It would be relevant to repeat this genotyping study on a larger population to determine whether these SNPs were identified and were more frequently present in an obese population. It would also be particularly interesting to verify whether these individuals had a much higher sweet taste detection threshold. However, TAS1R2-I595T, which showed a decrease in potency and efficacy when stimulated by different sweet compounds and even a lack of response in the presence of saccharin and NHDC in our study, was not associated with a moderate or high impact on the functionality of the sweet taste receptor. Furthermore, sequencing analysis revealed that the TAS1R2 variants I191V and S773S had no effect on the functionality of this taste receptor in contrast to in vivo studies that demonstrated differences related to sweet taste perception and sugar consumption for these variants [49,87]. For the other variants identified by sequencing analysis, although the cellular results revealed slight differences in efficacy and potency according to the different sweeteners with TAS1R2-WT and TAS1R3-WT, these results were consistent with the prediction data. Finally, our data suggested that factors other than the *TAS1R2* and *TAS1R3* SNPs were associated with obesity [74,77,78,79,80,81,82,83,84].

## 5. Conclusions

In conclusion, our data provide a better understanding of the effects of the most frequent SNPs in *TAS1R2*/*TAS1R3* on the expression and function of the whole sweet taste receptor in the presence of different sweeteners. They also offered, for the first time, an approach for measuring the influence of polymorphisms on the molecular pharmacology of the receptor. This in vitro cell-based assay could be relevant for future research to evaluate the impact of SNPs on the receptor functionality before identifying a potential correlation observed between polymorphisms and eating behaviours. Our results did not necessarily show a clear link between certain human phenotypes (higher sugar intake and/or higher sweet detection threshold) and the cellular response of associated SNPs, suggesting that *TAS1R2* and *TAS1R3* SNPs are probably not the only factors associated with obesity. Finally, blood sample polymorphism analysis revealed that seven novel SNPs were correlated with a potential loss of function of the corresponding sweet receptor subunit. It would be interesting to combine phenotypic (sensory and eating behaviour data), genetic and cellular analyses generated on a cohort of obese people representative of the population to confirm whether there was a correlation between these SNPs and obesity.

## Figures and Tables

**Table 1:** Single-nucleotide polymorphisms (SNPs) studied in the *TAS1R2* and *TAS1R3* gene regions.

Gene	SNP Reference Sequence	Allele ^a^	mRNA Position	Amino Acid Encoded	Position in Protein	Domain Within Sweet Taste Receptor ^b^	Variant Name Studied	Minor Allele Frequency ^c^	References
*TAS1R2*	rs9701796	G C	27	Ser (S) Cys (C)	9	Signal peptide	C9S	0.202	[48,49,50,51,58,59,60]
	rs72953144	G A	63	Pro (P) Leu (L)	21	VFT	P21L	0.022	[48]
	rs35874116	T C	572	Ile (I) Val (V)	191	VFT	I191V	0.267	[29,48,49,50,51,58,61,62]
	rs74604275	T C	634	Ser (S) Gly (G)	212	VFT	S212G	0.004	[48]
	rs139655863	T C	700	Ile (I) Val (V)	234	VFT	I234V	0.005	[48]
	rs34545913 rs34447754	C>G G>C	951	Arg (R) Pro (P) Gly (G)	317	VFT	R317P R317G	0.014 0.238	[48]
	rs28374389	T C	1457	Ile (I) Val (V)	486	CRD	I486V	0.126	[48,59,60]
	rs6662276	C A	1721	Ala (A) Thr (T)	574	TM1	A574T	0.091	[48,58,59,60]
	rs41273167	A G	1785	Ile (I) Thr (T)	595	ICL1	I595T	0.012	
	rs1212135598	C T	2065	Lys (K) Gln (Q)	689	TM4	K689Q	<0.001	[48]
	rs9988418	C T	2514	Arg (R) Lys (K)	838	C-terminal	R838K	0.058	[48,59]
*TAS1R3*	rs76755863	G A	13	Ala (A) Thr (T)	5	Signal peptide	A5T	0.019	[48,63]
	rs776847816	T C	284	Leu (L) Pro (P)	95	VFT	L95P	<0.001	[48]
	rs746577143	T C	329	Met (M) Thr (T)	110	VFT	M110T	<0.001	[45]
	rs111615792	G A	740	Arg (R) His (H)	247	VFT	R247H	0.067	[48,63]
	rs147600530	G T	1099	Gly (G) Cys (C)	367	VFT	G367C	0.016	[48]
	rs141949092	G A	1338	Ser (S) Asn (N)	446	VFT	S446N	<0.001	[58]
	rs200580453	C G	1542	Phe (F) Leu (L)	514	VFT	F514L	<0.001	[58]
	rs1425170639	C A	1652	Ser (S) Asn (N)	551	VFT	S551N	<0.001	[45]
	rs147441599	C A	2148	Thr (T) Met (M)	716	ECL2	T716M	<0.001	[58]
	rs112507608	G A	2203	Ala (A) Thr (T)	735	TM5	A735T	0.060	[48]
	rs79148073	T C	2246	Phe (F) Ser (S)	749	TM5	F749S	<0.001	[43]
	rs307377	T C	2269	Cys (C) Arg (R)	757	ICL3	C757R	0.048	[29,44,48,59,60,63]
	rs199779671	C G	2340	Pro (P) Ala (A)	780	TM6	P780A	<0.001	[58]
	rs34810828	G A	2439	Lys (K) Arg (R)	813	TM7	R813K	0.006	[44,45]
	rs12030797	C A	2469	Phe (F) Leu (L)	823	TM7	L823F	<0.001	[44,59,60]
	rs35913253	G A	2496	Arg (R) Gly (G)	832	C-terminal	G832R	<0.001	[44]

^a^ Alleles ranked by frequency. ^b^ Based on the annotation provided in UniProt Q8TE23. ^c^ Frequency taken from the 1000 Genome, TOPMED or HapMap dataset accessible via the dbSNP database (https://www.ncbi.nlm.nih.gov/snp/, accessed on 1 September 2019). VFT: Venus flytrap; CRD: cysteine-rich domain; TM: transmembrane domain; ECL: extracellular loop; ICL: intracellular loop.

**Table 2:** Blood samples were collected from the 5 centres involved in the study.

	Food Products			
Centres	Beverages	Biscuits	Cereal, Yogurt and Chocolate	Total/ Centre
CRNH	-	24	-	24
UNAV	23	-	27	50
UCPH	21	-	8	29
UNILIV	-	-	30	30
UNILEEDS	-	29	-	29
Total/product	44	53	65	162

CRNH (Rhône-Alpes Research Centre for Human Nutrition, Lyon, France); UNAV (University of Navarra, Pamplona, Spain); UCPH (University of Copenhagen, Denmark); UNILIV (University of Liverpool, UK); UNILEEDS (University of Leeds, UK).

**Table 3:** Experimental EC_50_ values and maximal signal amplitude values for sweet compounds obtained from cells cotransfected with mTAS1R2-WT and mTAS1R3-WT or mTAS1R3-I60T.

	mTAS1R3-WT		mTAS1R3-I60T			
Sweeteners	EC_50_ (µM)	Max ΔF/F_0_	EC_50_ (µM)	Max ΔF/F_0_	R1	R2
Sucralose	778 ± 22	2.03 ± 0.02	1520 ± 56	1.47 ± 0.03	2.0	0.7
Neotame	-	-	-	-	-	-
Acesulfame K	439 ± 10	1.63 ± 0.02	744 ± 19	1.03 ± 0.02	1.7	0.6
Rebaudioside A	41 ± 2	1.37 ± 0.02	78 ± 3	0.62 ± 0.01	1.9	0.5
Rebaudioside M	44 ± 4	1.41 ± 0.05	73 ± 2	0.71 ± 0.01	1.7	0.5
Mogroside V	133 ± 6	0.84 ± 0.02	225 ± 25	0.50 ± 0.03	1.7	0.5
Saccharin	1521 ± 17	1.71 ± 0.01	2539 ± 61	0.96 ± 0.02	1.7	0.6
Cyclamate	-	-	-	-	-	-
NHDC	-	-	-	-	-	-
Thaumatin	-	-	-	-	-	-
Brazzein *	-	-	-	-	-	-
Perillartine	-	-	-	-	-	-

* The EC_50_ value is expressed in g/L for brazzein. WT: wild type; NHDC: neohesperidin dihydrochalcone; “-”: no activation. R1 = Variant EC_50_/WT EC_50_; R1 > 1 represents a decrease in agonist potency at the receptor level. R2 = (Variant Max ΔF/F_0_)/(WT Max ΔF/F_0_); R2 < 1 represents a decrease in agonist efficacy at the receptor level.

**Table 4:** Experimental EC_50_ values and maximal signal amplitude values for selected sweeteners measured with TAS1R2-WT or selected TAS1R2 variants and TAS1R3-WT. For perillartine, the measure was carried out on the subunit alone (TAS1R2) and on the heterodimeric sweet taste receptor (TAS1R2/TAS1R3).

	**Sucralose**				**Neotame**				**Acesulfame K**			
**Variant**	**EC_50_ (µM)**	**R1**	**Max ΔF/F_0_**	**R2**	**EC_50_ (µM)**	**R1**	**Max ΔF/F_0_**	**R2**	**EC_50_ (µM)**	**R1**	**Max ΔF/F_0_**	**R2**
WT	90 ± 11	1.0	0.59 ± 0.03	1.0	0.84 ± 0.13	1.0	0.56 ± 0.03	1.0	705 ± 7	1.0	0.31 ± 0.01	1.0
C9S	88 ± 15	1.0	0.72 ± 0.05	1.2	0.85 ± 0.16	1.0	0.66 ± 0.04	1.2	466 ± 54	0.7	0.32 ± 0.01	1.0
P21L	80 ± 13	0.9	0.50 ± 0.03	0.8	0.67 ± 0.16	0.8	0.53 ± 0.04	0.9	374 ± 11	0.5	0.24 ± 0.01	0.8
I191V	87 ± 7	1.0	0.52 ± 0.02	0.9	0.84 ± 0.08	1.0	0.64 ± 0.02	1.1	492 ± 30	0.7	0.27 ± 0.01	0.9
S212G	96 ± 6	1.1	0.64 ± 0.02	1.1	0.99 ± 0.05	1.2	0.63 ± 0.01	1.1	438 ± 25	0.6	0.28 ± 0.01	0.9
I234V	60 ± 7	0.7	0.75 ± 0.03	1.3	0.80 ± 0.08	1.0	0.66 ± 0.02	1.2	510 ± 63	0.7	0.41 ± 0.02	1.3
R317G	62 ± 11	0.7	0.80 ± 0.05	1.4	0.73 ± 0.13	0.9	0.56 ± 0.03	1.0	465 ± 55	0.7	0.46 ± 0.02	1.5
R317P	98 ± 19	1.1	0.76 ± 0.06	1.3	0.86 ± 0.16	1.0	0.63 ± 0.04	1.1	685 ± 41	1.0	0.42 ± 0.01	1.4
I486V	54 ± 5	0.6	0.69 ± 0.02	1.2	0.71 ± 0.06	0.8	0.61 ± 0.01	1.1	778 ± 108	1.1	0.46 ± 0.03	1.5
A574T	81 ± 2	0.9	0.63 ± 0.01	1.1	0.73 ± 0.03	0.9	0.60 ± 0.01	1.1	658 ± 65	0.9	0.34 ± 0.02	1.1
I595T	108 ± 5	1.2	0.08 ± 0.02	0.1	1.27 ± 0.18	1.5	0.09 ± 0.01	0.2	300 ± 139	0.4	0.02 ± 0.01	0.1
K689Q	307 ± nd	3.4	0.08 ± 0.01	0.1	3.58 ± 0.46	4.3	0.12 ± 0.01	0.2	3000 ± ns	4.3	0.02 ± nd	0.1
R838K	82 ± 6	0.9	0.75 ± 0.02	1.3	0.66 ± 0.05	0.8	0.75 ± 0.02	1.3	484 ± 47	0.7	0.42 ± 0.02	1.4
	Rebaudioside A				Rebaudioside M				Mogroside V			
Variant	EC_50_ (µM)	R1	Max ΔF/F_0_	R2	EC_50_ (µM)	R1	Max ΔF/F_0_	R2	EC_50_ (µM)	R1	Max ΔF/F_0_	R2
WT	22.8 ± 1.0	1.0	1.06 ± 0.02	1.0	16.9 ± 0.5	1.0	1.12 ± 0.01	1.0	17.3 ± 1.2	1.0	1.28 ± 0.03	1.0
C9S	26.4 ± 0.5	1.2	1.18 ± 0.01	1.1	23.7 ± 1.6	1.4	1.18 ± 0.03	1.1	18.6 ± 1.6	1.1	1.32 ± 0.04	1.0
P21L	25.3 ± 0.5	1.1	1.09 ± 0.01	1.0	19.5 ± 0.9	1.2	0.99 ± 0.02	0.9	19.9 ± 1.1	1.2	1.18 ± 0.02	0.9
I191V	28.2 ± 1.8	1.2	1.19 ± 0.03	1.1	21.0 ± 0.4	1.2	1.10 ± 0.01	1.0	19.2 ± 1.7	1.1	1.33 ± 0.04	1.0
S212G	25.5 ± 0.6	1.1	1.16 ± 0.01	1.1	23.2 ± 1.1	1.4	1.14 ± 0.02	1.0	21.2 ± 2.1	1.2	1.32 ± 0.05	1.0
I234V	28.4 ± 3.8	1.2	1.06 ± 0.06	1.0	18.4 ± 0.6	1.1	1.14 ± 0.01	1.0	19.4 ± 1.6	1.1	1.28 ± 0.04	1.0
R317G	27.2 ± 1.0	1.2	1.07 ± 0.02	1.0	20.4 ± 0.4	1.2	1.23 ± 0.01	1.1	18.4 ± 1.0	1.1	1.34 ± 0.03	1.0
R317P	37.2 ± 3.8	1.6	1.15 ± 0.05	1.1	26.9 ± 0.5	1.6	1.19 ± 0.01	1.1	27.9 ± 2.5	1.6	1.51 ± 0.05	1.2
I486V	22.7 ± 1.1	1.0	1.06 ± 0.02	1.0	17.6 ± 0.6	1.0	1.20 ± 0.01	1.1	20.1 ± 0.9	1.2	1.39 ± 0.02	1.1
A574T	19.8 ± 1.3	0.9	0.98 ± 0.02	0.9	16.3 ± 0.5	1.0	1.08 ± 0.01	1.0	16.7 ± 3.1	1.0	1.25 ± 0.08	1.0
I595T	27.6 ± 2.0	1.2	0.16 ± 0.01	0.2	35.8 ± 4.0	2.1	0.23 ± 0.01	0.2	31.4 ± 6.0	1.8	0.27 ± 0.03	0.2
K689Q	77.0 ± 5.7	3.4	0.29 ± 0.01	0.3	75.8 ± 2.3	4.5	0.36 ± 0.01	0.3	57.9 ± 0.4	3.3	0.54 ± 0.01	0.4
R838K	23.0 ± 1.4	1.0	1.09 ± 0.03	1.0	18.2 ± 1.1	1.1	1.20 ± 0.01	1.1	17.1 ± 0.9	1.0	1.31 ± 0.02	1.0
	Saccharin				Cyclamate				NHDC			
Variant	EC_50_ (µM)	R1	Max ΔF/F_0_	R2	EC_50_ (µM)	R1	Max ΔF/F_0_	R2	EC_50_ (µM)	R1	Max ΔF/F_0_	R2
WT	274.4 ± 4.1	1.0	0.64 ± 0.01	1.0	1889 ± 46	1.0	0.87 ± 0.01	1.0	76.7 ± 4.4	1.0	0.71 ± 0.02	1.0
C9S	370.6 ± 38.3	1.3	0.55 ± 0.03	0.9	1806 ± 55	1.0	0.86 ± 0.01	1.0	99.0 ± 5.2	1.3	0.83 ± 0.02	1.2
P21L	352.6 ± 32.5	1.3	0.46 ± 0.03	0.7	2114 ± 55	1.1	0.86 ± 0.01	1.0	100.1 ± 5.1	1.3	0.81 ± 0.02	1.1
I191V	285.0 ± 2.5	1.0	0.37 ± 0.01	0.6	2017 ± 31	1.1	0.88 ± 0.01	1.0	108.5 ± 4.6	1.4	0.73 ± 0.01	1.0
S212G	304.6 ± 9.3	1.1	0.51 ± 0.01	0.8	1767 ± 44	0.9	0.92 ± 0.01	1.1	90.7 ± 8.1	1.2	0.84 ± 0.03	1.2
I234V	289.0 ± 14.4	1.0	0.66 ± 0.02	1.0	2250 ± 42	1.2	1.07 ± 0.01	1.2	94.7 ± 6.8	1.2	0.80 ± 0.02	1.1
R317G	224.5 ± 6.1	0.8	0.84 ± 0.01	1.3	2161 ± 44	1.1	1.15 ± 0.01	1.3	97.1 ± 5.0	1.3	0.85 ± 0.02	1.2
R317P	356.6 ± 24.2	1.3	0.38 ± 0.02	0.6	2858 ± 62	1.5	0.80 ± 0.01	0.9	171.3 ± 26.2	2.2	0.58 ± 0.04	0.8
I486V	293.6 ± 11.6	1.1	0.67 ± 0.02	1.0	1876 ± 36	1.0	1.08 ± 0.01	1.2	92.6 ± 5.2	1.2	0.92 ± 0.02	1.3
A574T	247.5 ± 15.7	0.9	0.57 ± 0.02	0.9	1783 ± 49	0.9	0.92 ± 0.01	1.1	73.4 ± 8.4	1.0	0.81 ± 0.03	1.1
I595T	-	-	-	-	2026 ± 102	1.1	0.06 ± 0.01	0.1	70.3 ± 1.3	0.9	0.09 ± 0.01	0.1
K689Q	-	-	-	-	5014 ± 220	2.7	0.41 ± 0.01	0.5	-	-	-	-
R838K	253.5 ± 5.0	0.9	0.65 ± 0.01	1.0	1734 ± 24	0.9	0.99 ± 0.01	1.1	75.8 ± 1.2	1.0	0.79 ± 0.05	1.1
	Thaumatin				Brazzein							
Variant	EC_50_ (µM)	R1	Max ΔF/F_0_	R2	EC_50_ (mg/L)	R1	Max ΔF/F_0_	R2				
WT	7.6 ± 0.3	1.0	0.72 ± 0.01	1.0		1.0	1.17 ± 0.04	1.0				
C9S	6.6 ± 0.1	0.9	0.61 ± 0.01	0.8	95.5 ± 9.7	1.2	1.14 ± 0.03	1.0				
P21L	3.3 ± nd	0.4	0.19 ± 0.04	0.3	111.8 ± 7.5	1.1	1.09 ± 0.01	0.9				
I191V	7.7 ± 0.9	1.0	0.34 ± 0.02	0.5	105.2 ± 35.7	1.7	1.12 ± 0.02	1.0				
S212G	6.1 ± 0.2	0.8	0.6 ± 0.01	0.8	161.6 ± 8.6	1.4	1.18 ± 0.02	1.0				
I234V	13.8 ± 0.2	1.8	1.25 ± 0.01	1.7	129.6 ± 8.2	1.1	1.34 ± 0.05	1.1				
R317G	10.9 ± 0.3	1.4	1.16 ± 0.02	1.6	100.8 ± 13.8	1.3	1.18 ± 0.03	1.0				
R317P	18.9 ± 0.7	2.5	0.42 ± 0.01	0.6	124.2 ± 12.4	2.5	0.91 ± 0.02	0.8				
I486V	10.5 ± 0.3	1.4	1.21 ± 0.02	1.7	234.2 ± 17.4	1.1	1.19 ± 0.03	1.0				
A574T	14.1 ± 0.3	1.9	1.37 ± 0.02	1.9	109.3 ± 9.8	1.2	1.14 ± 0.01	1.0				
I595T	27.8 ± 1.4	3.7	0.39 ± 0.01	0.5	116.2 ± 5.1	10.8	0.13 ± 0.02	0.1				
K689Q	33.5 ± nd	4.4	0.87 ± 0.09	1.2	1035.0 ± 143.2	5.7	0.64 ± 0.03	0.5				
R838K	10.4 ± 0.2	1.4	1.3 ± 0.02	1.8	545.7 ± 68.2	1.2	1.2 ± 0.03	1.0				
	Perillartine (TAS1R2)				Perillartine (TAS1R2/TAS1R3)							
Variant	EC_50_ (µM)	R1	Max ΔF/F_0_	R2	EC_50_ (µM)	R1	Max ΔF/F_0_	R2				
WT	90.0 ± 17.0	1.0	1.06 ± 0.06	1.0	17.5 ± 2.2	1.0	1.47 ± 0.07	1.0				
C9S	189.6 ± 26.6	2.1	0.44 ± 0.02	0.4	17.8 ± 1.2	1.0	1.29 ± 0.03	0.9				
P21L	148.6 ± 22.7	1.7	0.33 ± 0.02	0.3	26.3 ± 1.1	1.5	1.35 ± 0.03	0.9				
I191V	389.6 ± 47.0	4.3	0.19 ± 0.01	0.2	27.9 ± 0.5	1.6	1.27 ± 0.01	0.9				
S212G	177.8 ± 22.7	2.0	0.47 ± 0.02	0.4	22.9 ± 0.6	1.3	1.30 ± 0.01	0.9				
I234V	311.3 ± 24.6	3.5	0.29 ± 0.01	0.3	20.1 ± 1.1	1.1	1.52 ± 0.03	1.0				
R317G	224.8 ± 31.9	2.5	0.17 ± 0.01	0.2	20.7 ± 0.6	1.2	1.53 ± 0.03	1.0				
R317P	-	-	-	-	35.2 ± 2.2	2.0	1.16 ± 0.03	0.8				
I486V	123.9 ± 14.1	1.4	0.39 ± 0.02	0.4	17.7 ± 1.2	1.0	1.49 ± 0.04	1.0				
ZA574T	85.3 ± 12.4	0.9	0.25 ± 0.01	0.2	19.2 ± 0.9	1.1	1.39 ± 0.02	0.9				
I595T	-	-	-	-	53.2 ± 1.6	3.0	0.51 ± 0.01	0.3				
K689Q	-	-	-	-	132.5 ± 16.9	7.6	1.36 ± 0.01	0.9				
R838K	92.4 ± 19.7	1.0	0.35 ± 0.03	0.3	19.1 ± 1.2	1.1	1.45 ± 0.03	1.0				

WT: wild type; NHDC: neohesperidin dihydrochalcone; “-”: no activation; “nd”: not determined. R1 = Variant EC_50_/WT EC_50_; R1 > 1 represents a decrease in agonist potency at the receptor level. R2 = (Variant Max ΔF/F_0_)/(WT Max ΔF/F_0_); R2 < 1 represents a decrease in agonist efficacy at the receptor level.

**Table 5:** Experimental EC_50_ values and maximal activity values for selected sweeteners measured with TAS1R2-WT and TAS1R3-WT or selected TAS1R3 variants.

	Sucralose				Neotame				Acesulfame K			
Variant	EC_50_ (µM)	R1	Max ΔF/F_0_	R2	EC_50_ (µM)	R1	Max ΔF/F_0_	R2	EC_50_ (µM)	R1	Max ΔF/F_0_	R2
WT	109 ± 4	1.0	0.65 ± 0.01	1.0	1.22 ± 0.11	1.0	0.65 ± 0.02	1.0	622 ± 45	1.0	0.28 ± 0.01	1.0
A5T	135 ± 5	1.2	0.63 ± 0.01	1.0	1.18 ± 0.05	1.0	0.65 ± 0.01	1.0	852 ± 34	1.4	0.31 ± 0.01	1.1
L95P	-	-	-	-	-	-	-	-	-	-	-	-
M110T	282 ± 101	2.6	0.11 ± 0.02	0.2	2.70 ± 0.29	2.2	0.19 ± 0.01	0.3	1057 ± 648	1.7	0.20 ± 0.01	0.7
R247H	122 ± 2	1.1	0.69 ± 0.01	1.1	1.12 ± 0.07	0.9	0.71 ± 0.02	1.1	639 ± 17	1.0	0.33 ± 0.01	1.2
G367C	182 ± 7	1.7	0.32 ± 0.01	0.5	1.95 ± 0.09	1.6	0.31 ± 0.01	0.5	443 ± 121	0.7	0.06 ± 0.01	0.2
S446N	155 ± 11	1.4	0.68 ± 0.02	1.0	1.22 ± 0.10	1.0	0.63 ± 0.02	1.0	487 ± 78	0.8	0.19 ± 0.01	0.7
F514L	141 ± 3	1.3	0.49 ± 0.01	0.8	1.23 ± 0.06	1.0	0.45 ± 0.01	0.7	442 ± 24	0.7	0.14 ± 0.01	0.5
S551N	-	-	-	-	-	-	-	-	-	-	-	-
T716M	161 ± 14	1.5	0.47 ± 0.02	0.7	1.30 ± 0.06	1.1	0.54 ± 0.01	0.8	739 ± 72	1.2	0.30 ± 0.01	1.1
A735T	151 ± 4	1.4	0.70 ± 0.01	1.1	0.93 ± 0.07	0.8	0.61 ± 0.02	0.9	685 ± 56	1.1	0.47 ± 0.02	1.7
F749S	252 ± 36	2.3	0.18 ± 0.01	0.3	1.82 ± 0.12	1.5	0.22 ± 0.01	0.3	410 ± 242	0.7	0.04 ± 0.01	0.1
C757R	113 ± 10	1.0	0.84 ± 0.03	1.3	0.88 ± 0.12	0.7	0.75 ± 0.03	1.2	468 ± 31	0.8	0.42 ± 0.01	1.5
P780A	149 ± 12	1.4	0.76 ± 0.03	1.2	1.03 ± 0.08	0.8	0.63 ± 0.02	1.0	421 ± 31	0.7	0.30 ± 0.01	1.1
R813K	120 ± 9	1.1	0.90 ± 0.03	1.4	0.98 ± 0.07	0.8	0.79 ± 0.02	1.2	433 ± 34	0.7	0.40 ± 0.01	1.4
L823F	134 ± 8	1.2	0.84 ± 0.02	1.3	1.10 ± 0.06	0.9	0.76 ± 0.01	1.2	376 ± 34	0.6	0.33 ± 0.01	1.2
G832R	81 ± 4	0.7	0.91 ± 0.02	1.4	0.80 ± 0.07	0.7	0.88 ± 0.02	1.4	468 ± 64	0.8	0.55 ± 0.03	2.0
	Rebaudioside A				Rebaudioside M				Mogroside V			
Variant	EC_50_ (µM)	R1	Max ΔF/F_0_	R2	EC_50_ (µM)	R1	Max ΔF/F_0_	R2	EC_50_ (µM)	R1	Max ΔF/F_0_	R2
WT	57.8 ± 5.2	1.0	0.63 ± 0.03	1.0	40.4 ± 2.9	1.0	0.69 ± 0.02	1.0	31.3 ± 0.8	1.0	0.93 ± 0.01	1.0
A5T	46.9 ± 8.6	0.8	1.05 ± 0.08	1.7	30.2 ± 1.5	0.7	1.06 ± 0.02	1.5	31.3 ± 1.0	1.0	0.97 ± 0.01	1.0
L95P	-	-	-	-	-	-	-	-	262.5 ± nd	8.4	0.08 ± nd	0.1
M110T	103.9 ± 9.5	1.8	0.26 ± 0.01	0.4	103.2 ± 18.0	2.6	0.38 ± 0.04	0.6	94.5 ± 8.4	3.0	0.43 ± 0.02	0.5
R247H	40.7 ± 5.3	0.7	1.05 ± 0.06	1.7	29.7 ± 2.6	0.7	1.15 ± 0.04	1.7	28.7 ± 1.6	0.9	1.12 ± 0.02	1.2
G367C	125.1 ± 4.0	2.2	0.40 ± 0.06	0.6	47.3 ± 1.9	1.2	0.35 ± 0.01	0.5	82.4 ± 5.2	2.6	0.60 ± 0.02	0.6
S446N	56.1 ± 9.1	1.0	0.68 ± 0.05	1.1	36.9 ± 3.6	0.9	0.64 ± 0.03	0.9	47.7 ± 1.5	1.5	0.86 ± 0.01	0.9
F514L	65.2 ± 9.0	1.1	0.65 ± 0.09	1.0	32.1 ± 2.5	0.8	0.64 ± 0.02	0.9	44.7 ± 2.8	1.4	0.79 ± 0.02	0.8
S551N	-	-	-	-	-	-	-	-	111.2 ± nd	3.6	0.03 ± nd	0.0
T716M	41.3 ± 3.4	0.7	1.20 ± 0.04	1.9	30.7 ± 4.0	0.8	1.10 ± 0.06	1.6	36.4 ± 1.0	1.2	1.01 ± 0.01	1.1
A735T	30.6 ± 1.7	0.5	1.27 ± 0.03	2.0	23.0 ± 1.9	0.6	1.24 ± 0.04	1.8	28.7 ± 2.2	0.9	1.17 ± 0.03	1.3
F749S	88.9 ± 11.3	1.5	0.52 ± 0.03	0.8	71.8 ± 9.6	1.8	0.54 ± 0.03	0.8	65.5 ± 5.4	2.1	0.47 ± 0.02	0.5
C757R	28.4 ± 1.7	0.5	1.10 ± 0.03	1.7	24.3 ± 2.2	0.6	1.13 ± 0.04	1.6	21.7 ± 1.0	0.7	1.03 ± 0.02	1.1
P780A	26.3 ± 0.2	0.5	1.14 ± 0.01	1.8	23.0 ± 2.6	0.6	1.21 ± 0.05	1.8	22.5 ± 2.2	0.7	1.13 ± 0.04	1.2
R813K	40.6 ± 4.1	0.7	1.12 ± 0.05	1.8	27.4 ± 3.4	0.7	1.18 ± 0.06	1.7	22.0 ± 1.3	0.7	1.14 ± 0.02	1.2
L823F	31.8 ± 1.0	0.6	1.21 ± 0.02	1.9	33.2 ± 5.4	0.8	1.35 ± 0.09	2.0	22.0 ± 1.8	0.7	1.18 ± 0.04	1.3
G832R	26.7 ± 2.6	0.5	1.36 ± 0.05	2.2	22.7 ± 3.2	0.6	1.38 ± 0.07	2.0	19.1 ± 1.7	0.6	1.23 ± 0.04	1.3
	Saccharin				Cyclamate				NHDC			
Variant	EC_50_ (µM)	R1	Max ΔF/F_0_	R2	EC_50_ (µM)	R1	Max ΔF/F_0_	R2	EC_50_ (µM)	R1	Max ΔF/F_0_	R2
WT	252.7 ± 6.4	1.0	0.75 ± 0.01	1.0	1591 ± 28	1.0	1.32 ± 0.01	1.0	50.3 ± 11.0	1.0	1.13 ± 0.01	1.0
A5T	302.2 ± 25.1	1.2	0.79 ± 0.04	1.1	1789 ± 35	1.1	1.28 ± 0.01	1.0	76.5 ± 13.1	1.5	1.24 ± 0.01	1.1
L95P	-	-	-	-	-	-	-	-	-	-	-	-
M110T	462.5 ± 41.4	1.8	0.15 ± 0.06	0.2	2617 ± 98	1.6	0.41 ± 0.01	0.3	202.6 ± 17.1	4.0	0.48 ± 0.02	0.4
R247H	209.6 ± 3.9	0.8	0.76 ± 0.01	1.0	1523 ± 46	1.0	1.13 ± 0.02	0.9	65.0 ± 8.9	1.3	1.14 ± 0.05	1.0
G367C	377.9 ± 28.3	1.5	0.40 ± 0.01	0.5	2124 ± 34	1.3	0.90 ± 0.01	0.7	81.3 ± 13.1	1.6	0.75 ± 0.05	0.7
S446N	243.4 ± 12.9	1.0	0.67 ± 0.02	0.9	1480 ± 45	0.9	1.23 ± 0.02	0.9	49.4 ± 8.6	1.0	1.07 ± 0.06	0.9
F514L	208.6 ± 5.7	0.8	0.51 ± 0.01	0.7	1250 ± 30	0.8	1.29 ± 0.01	1.0	37.9 ± 8.4	0.8	1.21 ± 0.08	1.1
S551N	-	-	-	-	-	-	-	-	-	-	-	-
T716M	257.9 ± 31.6	1.0	0.59 ± 0.04	0.8	1855 ± 45	1.2	1.03 ± 0.01	0.8	55.4 ± 18.4	1.1	0.84 ± 0.09	0.7
A735T	272.4 ± 24.8	1.0	0.89 ± 0.04	1.2	1869 ± 32	1.2	1.20 ± 0.01	0.9	42.2 ± 1.4	0.8	1.00 ± 0.01	0.9
F749S	439.7 ± 39.5	1.7	0.27 ± 0.01	0.4	3433 ± 1986	2.2	1.26 ± 0.04	1.0	149.8 ± 19.6	3.0	0.29 ± 0.01	0.3
C757R	170.3 ± 10.4	0.7	0.75 ± 0.02	1.0	1150 ± 54	0.7	1.16 ± 0.03	0.9	36.5 ± 16.0	0.7	0.98 ± 0.01	0.9
P780A	247.1 ± 29.0	1.0	0.89 ± 0.05	1.2	1166 ± 62	0.7	1.04 ± 0.03	0.8	46.4 ± 12.9	0.9	1.50 ± 0.01	1.3
R813K	156.4 ± 5.9	0.6	0.83 ± 0.01	1.1	1331 ± 22	0.8	1.15 ± 0.01	0.9	34.2 ± 15.2	0.7	1.00 ± 0.02	0.9
L823F	162.5 ± 5.2	0.6	0.83 ± 0.01	1.1	1369 ± 34	0.9	1.24 ± 0.01	0.9	43.1 ± 14.7	0.9	1.00 ± 0.01	0.9
G832R	186.4 ± 6.6	0.7	1.02 ± 0.02	1.4	998 ± 46	0.6	1.25 ± 0.03	0.9	28.1 ± 12.3	0.6	1.03 ± 0.01	0.9
	Thaumatin				Brazzein				Perillartine			
Variant	EC_50_ (µM)	R1	Max ΔF/F_0_	R2	EC_50_ (mg/L)	R1	Max ΔF/F_0_	R2	EC_50_ (µM)	R1	Max ΔF/F_0_	R2
WT	10.4 ± 0.6	1.0	0.57 ± 0.02	1.0	112.8 ± 11.2	1.0	0.88 ± 0.03	1.0	13.8 ± 6.3	1.0	1.51 ± 0.22	1.0
A5T	9.7 ± 0.8	0.9	0.73 ± 0.04	1.3	108.3 ± 11.5	1.0	1.04 ± 0.04	1.2	13.2 ± 6.4	1.0	1.42 ± 0.23	0.9
L95P	27.4 ± 5.8	2.6	0.89 ± 0.01	1.6	2178.0 ± 616.0	19.3	0.10 ± 0.05	0.1	33.9 ± 2.8	2.5	0.14 ± 0.02	0.1
M110T	16.3 ± 0.9	1.6	0.38 ± 0.01	0.7	221.5 ± 11.3	2.0	0.68 ± 0.01	0.8	41.1 ± 19.4	3.0	1.31 ± 0.21	0.9
R247H	8.2 ± 2.3	0.8	0.73 ± 0.08	1.3	70.8 ± 8.1	0.6	0.95 ± 0.03	1.1	8.5 ± 3.1	0.6	1.26 ± 0.15	0.8
G367C	14.8 ± 0.5	1.4	0.52 ± 0.01	0.9	181.7 ± 6.9	1.6	0.72 ± 0.01	0.8	32.0 ± 17.1	2.3	1.49 ± 0.24	1.0
S446N	9.8 ± 1.1	0.9	0.70 ± 0.04	1.2	120.8 ± 9.3	1.1	0.98 ± 0.03	1.1	15.3 ± 8.6	1.1	1.42 ± 0.26	0.9
F514L	9.8 ± 1	0.9	0.72 ± 0.04	1.3	44.4 ± 6.4	0.4	1.07 ± 0.04	1.2	13.9 ± 7.3	1.0	1.40 ± 0.24	0.9
S551N	17.4 ± 2.2	1.7	0.25 ± 0.01	0.4	2378.0 ± 1623.0	21.1	0.21 ± 0.21	0.2	49.2 ± 13.6	3.6	0.52 ± 0.04	0.3
T716M	10.0 ± 0.5	1.0	0.76 ± 0.04	1.3	112.6 ± 13.6	1.0	0.94 ± 0.04	1.1	21.6 ± 11.4	1.6	1.73 ± 0.33	1.1
A735T	7.2 ± 1.5	0.7	0.70 ± 0.06	1.2	82.7 ± 14.9	0.7	0.90 ± 0.05	1.0	12.6 ± 5.9	0.9	1.56 ± 0.23	1.0
F749S	31.2 ± 3.5	3.0	0.42 ± 0.03	0.7	212.2 ± 8.6	1.9	0.65 ± 0.01	0.7	12.1 ± 6.1	0.9	1.90 ± 0.30	1.3
C757R	6.7 ± 1.4	0.6	0.69 ± 0.05	1.2	74.2 ± 1.9	0.7	0.86 ± 0.06	1.0	8.1 ± 2.6	0.6	1.23 ± 0.13	0.8
P780A	7.8 ± 1.3	0.8	0.86 ± 0.06	1.5	80.7 ± 14.8	0.7	1.06 ± 0.06	1.2	12.7 ± 5	0.9	1.44 ± 0.20	1.0
R813K	6.5 ± 0.9	0.6	0.74 ± 0.04	1.3	58.0 ± 10.5	0.5	1.00 ± 0.05	1.1	10.4 ± 3.6	0.8	1.28 ± 0.16	0.8
L823F	7.4 ± 1.1	0.7	0.71 ± 0.04	1.2	78.9 ± 14.2	0.7	0.98 ± 0.05	1.1	10.0 ± 3.1	0.7	1.39 ± 0.15	0.9
G832R	5.7 ± 0.7	0.5	0.76 ± 0.03	1.3	52.8 ± 15.7	0.5	0.97 ± 0.07	1.1	7.2 ± 3.0	0.5	1.18 ± 0.14	0.8

WT: wild type; NHDC: neohesperidin dihydrochalcone; “-”: no activation; “nd”: not determined. R1 = Variant EC_50_/WT EC_50_; R1 > 1 represents a decrease in agonist potency at the receptor level. R2 = (Variant Max ΔF/F_0_)/(WT Max ΔF/F_0_); R2 < 1 represents a decrease in agonist efficacy at the receptor level.

**Table 6:** *TAS1R2*/*TAS1R3* SNP sequencing results from an obese population and possible/probable SNPs associated with the loss of sweet taste receptor function.

Gene	Chromosome 1 Position	Ref	Alt	Variant Name	Tier	Frequency in the Study	Frequency in gnomAD	Rare Variant	Consequence	Impact	Damaging Effects Predicted (%)	Existing Variation
	18859598	C	T	P21P	NT	0.003	0.003	T	Synonymous	L		rs144454001
	18854834	G	A	S212S	NT	0.003	<0.001	T	Synonymous	L		rs371978810
	18839767	G	A	S784S	NT	0.003	<0.001	T	Synonymous	L		rs138899345
	18839650	C	T	T823T	NT	0.003	<0.001	T	Synonymous	L		rs774217162 and COSV64746111
	18840172	G	A	A649A	NT	0.009	0.002	T	Synonymous	L		rs115672344
	18840007	G	A	P704P	NT	0.012	0.010	T	Synonymous	L		rs34542537
	18840400	G	A	A573A	NT	0.018	0.046	T	Synonymous	L		rs11805253
	18839749	G	A	I790I	NT	0.040	0.049	T	Synonymous	L		rs12075191
	18854588	A	C	T294T	NT	0.281	0.291	F	Synonymous	L		rs28470550
	18857583	A	G	F77F	NT	0.293	0.320	F	Synonymous	L		rs68081213
	18839800	G	A	S773S	NT	0.302	0.294	F	Synonymous	L		rs12033832
	18839871	T	G	**K750Q**	2	0.003	-	T	Missense	M	probably damaging (100)	rs933473941
***TAS1R2***	18841752	C	T	**G523D**	2	0.003	<0.001	T	Missense	M	probably damaging (100)	rs766687403
	18854355	G	A	**S372F**	2	0.003	-	T	Missense	M	possibly damaging (87.6)	rs535257286 and COSV64747888
	18854529	G	T	**T314K**	2	0.003	<0.001	T	Missense	M	possibly damaging (48.4)	rs148292629
	18840335	A	G	*I595T*	2	0.040	0.017	T	Missense	M	benign (7.7)	rs41273167
	18849382	T	G	K476Q	3	0.003	<0.001	T	Missense	M	benign (10.7)	rs547302644
	18854723	C	T	M249I	3	0.003	0.003	T	Missense	M	benign (0.0)	rs148245865
	18859599	G	A	*P21L*	3	0.006	0.021	T	Missense	M	benign (0.3)	rs72953144
	18849511	C	T	*D433N*	3	0.009	0.002	T	Missense	M	benign (3.1)	rs114026861 and COSV64745359
	18839606	C	T	*R838K*	3	0.018	0.052	F	Missense	M	benign (0.0)	rs9988418
	18840399	C	T	*A574T*	3	0.040	0.056	F	Missense	M	benign (0.7)	rs6662276
	18849352	T	C	*I486V*	3	0.197	0.190	F	missense	M	benign (0.0)	rs28374389
	18854521	G	C	*R317G*	3	0.278	0.291	F	missense	M	benign (0.5)	rs34447754
	18854899	T	C	*I191V*	3	0.290	0.312	F	missense	M	benign (0.1)	rs35874116 and CM109811
	18859635	G	C	S9C	3	0.799	0.787	F	missense	M	benign (0.0)	rs9701796
	1333642	G	A	A579A	NT	0.003	0.004	T	synonymous	L	.	rs143667857
	1333882	C	T	F659F	NT	0.003	<0.001	T	synonymous	L	.	rs142902721
	1334422	C	T	G839G	NT	0.003	0.002	T	synonymous	L	.	rs148758835
	1332968	G	A	P441P	NT	0.003	0.024	T	synonymous	L	.	rs111703380
	1332116	G	A	T195T	NT	0.003	0.008	T	synonymous	L	.	rs146097837
	1332260	G	A	V243V	NT	0.003	<0.001	T	synonymous	L	.	rs1258667478
	1333007	C	T	Y454Y	NT	0.003	0.001	T	synonymous	L	.	rs142857537 and COSV59566129
	1331677	A	G	K77K	NT	0.006	0.005	T	synonymous	L	.	rs139515618
	1333082	G	A	R479R	NT	0.006	0.007	T	synonymous	L	.	rs138915131
	1331905	C	T	T153T	NT	0.006	0.001	T	synonymous	L	.	rs147731455
	1333624	C	T	L573L	NT	0.015	0.007	T	synonymous	L	.	rs140035477 and COSV59564228
	1331360	T	A	A5A	NT	0.065	0.045	T	synonymous	L	.	rs141430443
***TAS1R3***	1332779	C	T	P416P	NT	0.068	0.122	F	synonymous	L	.	rs3813210
	1333908	G	A	W668	1	0.003	<0.001	T	nonsense	H	.	rs147921760
	1331660	T	G	**W72G**	2	0.003	<0.001	T	missense	M	possibly damaging (81.1)	rs144594741
	1331726	C	T	**R94C**	2	0.003	<0.001	T	missense	M	possibly damaging (86.2)	rs138021134
	1333116	G	C	**D491H**	2	0.003	<0.001	T	missense	M	possibly damaging (56.4)	rs1358129448
	1334232	TCTC	T	VS776-777V	2	0.003	<0.001	T	indel	M	.	rs531899606
	1333090	G	A	R482H	3	0.003	<0.001	T	missense	M	benign (0.3)	rs143388404
	1334382	C	T	P826L	3	0.003	<0.001	T	missense	M	benign (0.3)	rs749544965
	1334108	G	A	*A735T*	3	0.006	0.063	F	missense	M	benign (1.1)	rs112507608
	1332271	G	A	*R247H*	3	0.025	0.073	F	missense	M	benign (0.5)	rs111615792
	1331358	G	A	*A5T*	3	0.065	0.045	T	missense	M	benign (2.1)	rs76755863
	1331358	GCT	ACA	*A5T*	3	0.065	.	T	missense	M	benign (2.1)	.
	1334174	T	C	*C757R*	3	0.966	0.972	F	missense	M	benign (0.0)	rs307377 and CM098260

Ref: reference nucleotide; Alt: altered nucleotide; Tier: synonymous variant (NT), truncating variant (nonsense, splice and frameshift) (1), missense variant predicted to be damaging by PolyPhen 2.2.2.2. (2), other missense variant (3); frequency in the study indicates the frequency of the altered allele among the participants [(nb heterozygote + (2 × nb homozygote))/((2 × nb participants))]; rare variant indicates that if the frequency in gnomAD is less than 0.050; impact: low (L), moderate (M) and high (H); damaging effects predicted by PolyPhen 2.2.2.2. (naïve Bayesian posterior probability); “-”: no damaging effect predicted. In italics, the SNP variants tested in our in vitro study and, in bold, the SNP variants probably or possibly causing damaging effects on sweet taste receptor function.

## Data Availability

The data presented in this study are available on request from the authors.

## Supplementary Materials

The following supporting information can be downloaded at https://www.mdpi.com/article/10.3390/nu17060949/s1. Protein sequence of human TAS1R2-WT; Protein sequence of human TAS1R3-WT; Table S1. *p*-values calculated for mTAS1R2-WT/mTAS1R3-I60T using ANOVA followed by Dunnett’s test of ΔF/F0 for each concentration of sweetener that elicited a response (with reference to mTAS1R2-WT/mTAS1R3-WT). Significative *p*-value are in bold (*p* < 0.05); Table S2. *p*-values calculated using ANOVA followed by Dunnett’s test for multiple comparison analysis of ΔF/F0 for each sweetener concentration and each TAS1R2-SNP/TAS1R3-WT (with reference to TAS1R2-WT/TAS1R3-WT). Significative *p*-value are in bold (*p* < 0.05); Table S3. *p*-values calculated using ANOVA followed by Dunnett’s test for multiple comparison analysis of ΔF/F0 for each sweetener concentration and each TAS1R2-WT/TAS1R3-SNP (with reference to TAS1R2-WT/TAS1R3-WT). Significative *p*-value are in bold (*p* < 0.05); Figure S1: Human TAS1R2-SNP/TAS1R3 dose–response curves with various sweeteners. HEK293T-Gα16gust44 cells were transiently transfected with pcDNA6-MAX-TAS1R2-WT-FLAG (blue line) or pcDNA6-MAX-TAS1R2-SNP-FLAG (red line) and pcDNA4-MAX-TAS1R3-FLAG. A total of 12 TAS1R2 variants were tested for each of the 11 sweeteners (A-L) (excluding of sucralose). For perillartine (L), HEK293T-Gα16gust44 cells were transiently transfected with pcDNA6-MAX-TAS1R2-WT-FLAG (blue dotted line) or pcDNA6-MAX-TAS1R2-SNP-FLAG (red dotted line) only. The data are presented as the mean ± SEM of 8 wells from 4 independent experiments. * *p* < 0.05, ** *p* < 0.01, *** *p* < 0.001, calculated using ANOVA followed by Dunnett’s test for multiple comparison analysis (with reference to TAS1R2-WT/TAS1R3-WT). The *p*-values are presented in Table S2. WT: wild type; AceK: acesulfame K; NHDC: neohesperidin dihydrochalcone; Figure S2: Human TAS1R2-SNP/TAS1R3 dose–response curves with various sweeteners. HEK293T-Gα16gust44 cells were transiently transfected with pcDNA6-MAX-TAS1R2-WT-FLAG (blue line) or pcDNA6-MAX-TAS1R2-SNP-FLAG (red line) and pcDNA4-MAX-TAS1R3-FLAG. A total of 12 TAS1R2 variants (A-L) and 12 sweeteners were tested. For perillartine, HEK293T-Gα16gust44 cells were transiently transfected with pcDNA6-MAX-TAS1R2-WT-FLAG (blue dotted line) or pcDNA6-MAX-TAS1R2-SNP-FLAG (red dotted line) only. The data are presented as the mean ± SEM of 8 wells from 4 independent experiments. * *p* < 0.05, ** *p* < 0.01, *** *p* < 0.001, calculated using ANOVA followed by Dunnett’s test for multiple comparison analysis (with reference to TAS1R2-WT/TAS1R3-WT). The *p*-values are presented in Table S2. WT: wild type; AceK: acesulfame K; NHDC: neohesperidin dihydrochalcone; Figure S3. Immunocytochemistry of HEK293T-Gα16gust44 cells expressing FLAG-tagged TAS1R2-SNPs constructs. The TAS1R-expressing cells are shown in green, and the plasma membrane is stained in red. The receptors were detected using a primary anti-FLAG antibody and fluorescently labelled by a secondary Alexa-488-conjugated antibody. All data were obtained from the same transfection experiment. HEK293T-Gα16gust44 cells in the absence of the TAS1R receptor (mock cells) showed no signal. Pictures were taken using an epi-fluorescence inverted microscope (Eclipse TiE, Nikon, Champigny sur Marne, France) equipped with an ×20 objective lens and a LucaR EMCCD camera (Andor Technology, Belfast, UK). The average cell fraction expressing the receptor (±sem) is provided in white in the overlay panel. Four to six images were counted and averaged per receptor construct pictures. WT: wild type; Figure S4: Human TAS1R2/TAS1R3-SNP dose–response curves with various sweeteners. HEK293T-Gα16gust44 cells were transiently transfected with pcDNA6-MAX-TAS1R2-FLAG and pcDNA4-MAX-TAS1R3-WT-FLAG (blue line) or pcDNA4-MAX-TAS1R3-SNP-FLAG (green line). A total of 16 TAS1R3 variants were tested for each of the 11 sweeteners (A-K) (excluding of sucralose). The data are presented as the mean ± SEM of 8 wells from 4 independent experiments. * *p* < 0.05, ** *p* < 0.01, *** *p* < 0.001, calculated using ANOVA followed by Dunnett’s test for multiple comparison analysis (with reference to TAS1R2-WT/TAS1R3-WT). The *p*-values are presented in Table S3. WT: wild type; AceK: acesulfame K; NHDC: neohesperidin dihydrochalcone; Figure S5: Human TAS1R2/TAS1R3-SNP dose–response curves with various sweeteners. HEK293T-Gα16gust44 cells were transiently transfected with pcDNA6-MAX-TAS1R2-FLAG and pcDNA4-MAX-TAS1R3-WT-FLAG (blue line) or pcDNA4-MAX-TAS1R3-SNP-FLAG (green line). A total of 16 TAS1R3 variants (A-P) and 12 sweeteners were tested. The data are presented as the mean ± SEM of 8 wells from 4 independent experiments. * *p* < 0.05, ** *p* < 0.01, *** *p* < 0.001, calculated using ANOVA followed by Dunnett’s test for multiple comparison analysis (with reference to TAS1R2-WT/TAS1R3-WT). The *p*-values are presented in Table S3. WT: wild type; AceK: acesulfame K; NHDC: neohesperidin dihydrochalcone; Figure S6. Immunocytochemistry of HEK293T-Gα16gust44 cells expressing FLAG-tagged TAS1R3-SNPs constructs. The TAS1R-expressing cells are shown in green, and the plasma membrane is stained in red. The receptors were detected using a primary anti-FLAG antibody and fluorescently labelled by a secondary Alexa-488-conjugated antibody. All data were obtained from the same transfection experiment. HEK293T-Gα16gust44 cells in the absence of the TAS1R receptor (mock cells) showed no signal. Pictures were taken using an epi-fluorescence inverted microscope (Eclipse TiE, Nikon, Champigny sur Marne, France) equipped with an ×20 objective lens and a LucaR EMCCD camera (Andor Technology, Belfast, UK). The average cell fraction expressing the receptor (±sem) is provided in white in the overlay panel. Four to six images were counted and averaged per receptor construct pictures. WT: wild type.

## References

[1] Briand L., Salles C., Etiévant P., Guichard E., Salles C., Voilley A. (2016). Taste Perception and Integration. Flavor from Food to Behaviors, Wellbeing and Health.

[2] Belloir C., Neiers F., Briand L. (2017). Sweeteners and Sweetness Enhancers. Curr. Opin. Clin. Nutr. Metab. Care.

[3] Malik V.S., Popkin B.M., Bray G.A., Després J.-P., Willett W.C., Hu F.B. (2010). Sugar-Sweetened Beverages and Risk of Metabolic Syndrome and Type 2 Diabetes: A Meta-Analysis. Diabetes Care.

[4] Peres M.A., Sheiham A., Liu P., Demarco F.F., Silva A.E.R., Assunção M.C., Menezes A.M., Barros F.C., Peres K.G. (2016). Sugar Consumption and Changes in Dental Caries from Childhood to Adolescence. J. Dent. Res..

[5] World Health Organization (2003). Diet, Nutrition, and the Prevention of Chronic Diseases: Report of a Joint WHO/FAO Expert Consultation.

[6] Li X., Staszewski L., Xu H., Durick K., Zoller M., Adler E. (2002). Human Receptors for Sweet and Umami Taste. Proc. Natl. Acad. Sci. USA.

[7] Nelson G., Hoon M.A., Chandrashekar J., Zhang Y., Ryba N.J.P., Zuker C.S. (2001). Mammalian Sweet Taste Receptors. Cell.

[8] Nelson G., Chandrashekar J., Hoon M.A., Feng L., Zhao G., Ryba N.J.P., Zuker C.S. (2002). An Amino-Acid Taste Receptor. Nature.

[9] Jiang P., Ji Q., Liu Z., Snyder L.A., Benard L.M.J., Margolskee R.F., Max M. (2004). The Cysteine-Rich Region of T1R3 Determines Responses to Intensely Sweet Proteins. J. Biol. Chem..

[10] Assadi-Porter F.M., Maillet E.L., Radek J.T., Quijada J., Markley J.L., Max M. (2010). Key Amino Acid Residues Involved in Multi-Point Binding Interactions between Brazzein, a Sweet Protein, and the T1R2–T1R3 Human Sweet Receptor. J. Mol. Biol..

[11] Masuda T., Taguchi W., Sano A., Ohta K., Kitabatake N., Tani F. (2013). Five Amino Acid Residues in Cysteine-Rich Domain of Human T1R3 Were Involved in the Response for Sweet-Tasting Protein, Thaumatin. Biochimie.

[12] Nie Y., Vigues S., Hobbs J.R., Conn G.L., Munger S.D. (2005). Distinct Contributions of T1R2 and T1R3 Taste Receptor Subunits to the Detection of Sweet Stimuli. Curr. Biol..

[13] Zhang F., Klebansky B., Fine R.M., Liu H., Xu H., Servant G., Zoller M., Tachdjian C., Li X. (2010). Molecular Mechanism of the Sweet Taste Enhancers. Proc. Natl. Acad. Sci. USA.

[14] Behrens M., Meyerhof W., Hellfritsch C., Hofmann T. (2011). Sweet and Umami Taste: Natural Products, Their Chemosensory Targets, and Beyond. Angew. Chem. Int. Ed..

[15] DuBois G.E. (2016). Molecular Mechanism of Sweetness Sensation. Physiol. Behav..

[16] Laffitte A., Belloir C., Neiers F., Briand L. (2022). Functional Characterization of the Venus Flytrap Domain of the Human TAS1R2 Sweet Taste Receptor. Int. J. Mol. Sci..

[17] Belloir C., Jeannin M., Karolkowski A., Scott C., Briand L. (2024). A Receptor-Based Assay to Study the Sweet and Bitter Tastes of Sweeteners and Binary Sweet Blends: The SWEET Project. Chem. Senses.

[18] Jiang P., Cui M., Ji Q., Snyder L., Liu Z., Benard L., Margolskee R.F., Osman R., Max M. (2005). Molecular Mechanisms of Sweet Receptor Function. Chem. Senses.

[19] Xu H., Staszewski L., Tang H., Adler E., Zoller M., Li X. (2004). Different Functional Roles of T1R Subunits in the Heteromeric Taste Receptors. Proc. Natl. Acad. Sci. USA.

[20] Liu B., Ha M., Meng X.-Y., Kaur T., Khaleduzzaman M., Zhang Z., Jiang P., Li X., Cui M. (2011). Molecular Mechanism of Species-Dependent Sweet Taste toward Artificial Sweeteners. J. Neurosci..

[21] Maillet E.L., Cui M., Jiang P., Mezei M., Hecht E., Quijada J., Margolskee R.F., Osman R., Max M. (2015). Characterization of the Binding Site of Aspartame in the Human Sweet Taste Receptor. Chem. Senses.

[22] Masuda K., Koizumi A., Nakajima K., Tanaka T., Abe K., Misaka T., Ishiguro M. (2012). Characterization of the Modes of Binding between Human Sweet Taste Receptor and Low-Molecular-Weight Sweet Compounds. PLoS ONE.

[23] Jiang P., Cui M., Zhao B., Snyder L.A., Benard L.M.J., Osman R., Max M., Margolskee R.F. (2005). Identification of the Cyclamate Interaction Site within the Transmembrane Domain of the Human Sweet Taste Receptor Subunit T1R3. J. Biol. Chem..

[24] Winnig M., Bufe B., Kratochwil N.A., Slack J.P., Meyerhof W. (2007). The Binding Site for Neohesperidin Dihydrochalcone at the Human Sweet Taste Receptor. BMC Struct. Biol..

[25] Jiang P., Cui M., Zhao B., Liu Z., Snyder L.A., Benard L.M.J., Osman R., Margolskee R.F., Max M. (2005). Lactisole Interacts with the Transmembrane Domains of Human T1R3 to Inhibit Sweet Taste. J. Biol. Chem..

[26] Morini G., Bassoli A., Temussi P.A. (2005). From Small Sweeteners to Sweet Proteins:  Anatomy of the Binding Sites of the Human T1R2_T1R3 Receptor. J. Med. Chem..

[27] Ohta K., Masuda T., Tani F., Kitabatake N. (2011). Introduction of a Negative Charge at Arg82 in Thaumatin Abolished Responses to Human T1R2–T1R3 Sweet Receptors. Biochem. Biophys. Res. Commun..

[28] Choi J.-H., Lee J., Yang S., Kim J. (2017). Genetic Variations in Taste Perception Modify Alcohol Drinking Behavior in Koreans. Appetite.

[29] Dotson C.D., Babich J., Steinle N.I. (2012). Genetic Predisposition and Taste Preference: Impact on Food Intake and Risk of Chronic Disease. Curr. Nutr. Rep..

[30] Lee R.J., Hariri B.M., McMahon D.B., Chen B., Doghramji L., Adappa N.D., Palmer J.N., Kennedy D.W., Jiang P., Margolskee R.F. (2017). Bacterial D-Amino Acids Suppress Sinonasal Innate Immunity through Sweet Taste Receptors in Solitary Chemosensory Cells. Sci. Signal..

[31] Lee R.J., Cohen N.A. (2015). Taste Receptors in Innate Immunity. Cell. Mol. Life Sci..

[32] Mfuna Endam L., Filali-Mouhim A., Boisvert P., Boulet L.-P., Bossé Y., Desrosiers M. (2014). Genetic Variations in Taste Receptors Are Associated with Chronic Rhinosinusitis: A Replication Study. Int. Forum Allergy Rhinol..

[33] Tarragon E., Moreno J.J. (2018). Role of Endocannabinoids on Sweet Taste Perception, Food Preference, and Obesity-Related Disorders. Chem. Senses.

[34] Triantafillou V., Workman A.D., Kohanski M.A., Cohen N.A. (2018). Taste Receptor Polymorphisms and Immune Response: A Review of Receptor Genotypic-Phenotypic Variations and Their Relevance to Chronic Rhinosinusitis. Front. Cell. Infect. Microbiol..

[35] Barham H.P., Taha M.A., Broyles S.T., Stevenson M.M., Zito B.A., Hall C.A. (2021). Association Between Bitter Taste Receptor Phenotype and Clinical Outcomes Among Patients with COVID-19. JAMA Netw. Open.

[36] Santin A., Spedicati B., Pecori A., Nardone G.G., Concas M.P., Piatti G., Menini A., Tirelli G., Boscolo-Rizzo P., Girotto G. (2024). The Bittersweet Symphony of COVID-19: Associations between TAS1Rs and TAS2R38 Genetic Variations and COVID-19 Symptoms. Life.

[37] Lin C., Civantos A.M., Arnold M., Stevens E.M., Cowart B.J., Colquitt L.R., Mansfield C., Kennedy D.W., Brooks S.G., Workman A.D. (2021). Divergent Bitter and Sweet Taste Perception Intensity in Chronic Rhinosinusitis Patients. Int. Forum Allergy Rhinol..

[38] Liszt K.I., Wang Q., Farhadipour M., Segers A., Thijs T., Nys L., Deleus E., der Schueren B.V., Gerner C., Neuditschko B. (2022). Human Intestinal Bitter Taste Receptors Regulate Innate Immune Responses and Metabolic Regulators in Obesity. J. Clin. Investig..

[39] Laffitte A., Neiers F., Briand L. (2014). Functional Roles of the Sweet Taste Receptor in Oral and Extraoral Tissues. Curr. Opin. Clin. Nutr. Metab. Care.

[40] Chamoun E., Liu A.S., Duizer L.M., Feng Z., Darlington G., Duncan A.M., Haines J., Ma D.W.L. (2021). Single Nucleotide Polymorphisms in Sweet, Fat, Umami, Salt, Bitter and Sour Taste Receptor Genes Are Associated with Gustatory Function and Taste Preferences in Young Adults. Nutr. Res..

[41] Garcia-Bailo B., Toguri C., Eny K.M., El-Sohemy A. (2009). Genetic Variation in Taste and Its Influence on Food Selection. OMICS.

[42] Bufe B., Breslin P.A.S., Kuhn C., Reed D.R., Tharp C.D., Slack J.P., Kim U.-K., Drayna D., Meyerhof W. (2005). The Molecular Basis of Individual Differences in Phenylthiocarbamide and Propylthiouracil Bitterness Perception. Curr. Biol..

[43] Raliou M., Grauso M., Hoffmann B., Schlegel–Le-Poupon C., Nespoulous C., Débat H., Belloir C., Wiencis A., Sigoillot M., Preet Bano S. (2011). Human Genetic Polymorphisms in T1R1 and T1R3 Taste Receptor Subunits Affect Their Function. Chem. Senses.

[44] Shigemura N., Shirosaki S., Ohkuri T., Sanematsu K., Islam A.S., Ogiwara Y., Kawai M., Yoshida R., Ninomiya Y. (2009). Variation in Umami Perception and in Candidate Genes for the Umami Receptor in Mice and Humans. Am. J. Clin. Nutr..

[45] Shigemura N., Shirosaki S., Sanematsu K., Yoshida R., Ninomiya Y. (2009). Genetic and Molecular Basis of Individual Differences in Human Umami Taste Perception. PLoS ONE.

[46] Fushan A.A., Simons C.T., Slack J.P., Manichaikul A., Drayna D. (2009). Allelic Polymorphism within the *TAS1R3* Promoter Is Associated with Human Taste Sensitivity to Sucrose. Curr. Biol..

[47] Mainland J.D., Matsunami H. (2009). Taste Perception: How Sweet It Is (To Be Transcribed by You). Curr. Biol..

[48] Kim U., Wooding S., Riaz N., Jorde L.B., Drayna D. (2006). Variation in the Human TAS1R Taste Receptor Genes. Chem. Senses.

[49] Eny K.M., Wolever T.M., Corey P.N., El-Sohemy A. (2010). Genetic Variation in *TAS1R2* (Ile191Val) Is Associated with Consumption of Sugars in Overweight and Obese Individuals in 2 Distinct Populations. Am. J. Clin. Nutr..

[50] Pioltine M.B., De Melo M.E., Santos A.S., Machado A.D., Fernandes A.E., Fujiwara C.T., Cercato C., Mancini M.C. (2018). Genetic Variations in Sweet Taste Receptor Gene Are Related to Chocolate Powder and Dietary Fiber Intake in Obese Children and Adolescents. J. Pers. Med..

[51] Dias A.G., Eny K.M., Cockburn M., Chiu W., Nielsen D.E., Duizer L., El-Sohemy A. (2015). Variation in the TAS1R2 Gene, Sweet Taste Perception and Intake of Sugars. J. Nutr. Nutr..

[52] Han P., Keast R.S.J., Roura E. (2017). Salivary Leptin and TAS1R2/TAS1R3 Polymorphisms Are Related to Sweet Taste Sensitivity and Carbohydrate Intake from a Buffet Meal in Healthy Young Adults. Br. J. Nutr..

[53] Liang Y., Yao J., Qiu R., Chen A., Huang H., Lin H., Yu L. (2022). The Rs35874116 Single Nucleotide Polymorphism Increases Sweet Intake and the Risk of Severe Early Childhood Caries: A Case–Control Study. BMC Oral Health.

[54] Melis M., Mastinu M., Naciri L.C., Muroni P., Tomassini Barbarossa I. (2022). Associations between Sweet Taste Sensitivity and Polymorphisms (SNPs) in the TAS1R2 and TAS1R3 Genes, Gender, PROP Taster Status, and Density of Fungiform Papillae in a Genetically Homogeneous Sardinian Cohort. Nutrients.

[55] Poirier N., Roudnitzky N., Brockhoff A., Belloir C., Maison M., Thomas-Danguin T., Meyerhof W., Briand L. (2012). Efficient Production and Characterization of the Sweet-Tasting Brazzein Secreted by the Yeast *Pichia pastoris*. J. Agric. Food Chem..

[56] Kim S.-K., Chen Y., Abrol R., Goddard W.A., Guthrie B. (2017). Activation Mechanism of the G Protein-Coupled Sweet Receptor Heterodimer with Sweeteners and Allosteric Agonists. Proc. Natl. Acad. Sci. USA.

[57] Cai C., Jiang H., Li L., Liu T., Song X., Liu B. (2016). Characterization of the Sweet Taste Receptor Tas1r2 from an Old World Monkey Species Rhesus Monkey and Species-Dependent Activation of the Monomeric Receptor by an Intense Sweetener Perillartine. PLoS ONE.

[58] Choi J.-H., Lee J., Choi I.J., Kim Y.-W., Ryu K.W., Kim J. (2016). Variations in TAS1R Taste Receptor Gene Family Modify Food Intake and Gastric Cancer Risk in a Korean Population. Mol. Nutr. Food Res..

[59] Reed D.R., Tanaka T., McDaniel A.H. (2006). Diverse Tastes: Genetics of Sweet and Bitter Perception. Physiol. Behav..

[60] Dotson C.D., Zhang L., Xu H., Shin Y.-K., Vigues S., Ott S.H., Elson A.E.T., Choi H.J., Shaw H., Egan J.M. (2008). Bitter Taste Receptors Influence Glucose Homeostasis. PLoS ONE.

[61] Melo S.V., Agnes G., Vitolo M.R., Mattevi V.S., Campagnolo P.D.B., Almeida S. (2017). Evaluation of the Association between the *TAS1R2* and *TAS1R3* Variants and Food Intake and Nutritional Status in Children. Genet. Mol. Biol..

[62] Ramos-Lopez O., Panduro A., Martinez-Lopez E., Roman S. (2016). Sweet Taste Receptor TAS1R2 Polymorphism (Val191Val) Is Associated with a Higher Carbohydrate Intake and Hypertriglyceridemia among the Population of West Mexico. Nutrients.

[63] Chen Q.-Y., Alarcon S., Tharp A., Ahmed O.M., Estrella N.L., Greene T.A., Rucker J., Breslin P.A. (2009). Perceptual Variation in Umami Taste and Polymorphisms in *TAS1R* Taste Receptor Genes123. Am. J. Clin. Nutr..

[64] Belloir C., Gautier A., Karolkowski A., Delompré T., Jeannin M., Moitrier L., Neiers F., Briand L. (2025). Optimized Vector for Functional Expression of the Human Bitter Taste Receptor TAS2R14 in HEK293 Cells. Protein Expr. Purif..

[65] Belloir C., Brulé M., Tornier L., Neiers F., Briand L. (2021). Biophysical and Functional Characterization of the Human TAS1R2 Sweet Taste Receptor Overexpressed in a HEK293S Inducible Cell Line. Sci. Rep..

[66] Slack J.P., Brockhoff A., Batram C., Menzel S., Sonnabend C., Born S., Galindo M.M., Kohl S., Thalmann S., Ostopovici-Halip L. (2010). Modulation of Bitter Taste Perception by a Small Molecule hTAS2R Antagonist. Curr. Biol..

[67] Ueda T., Ugawa S., Yamamura H., Imaizumi Y., Shimada S. (2003). Functional Interaction between T2R Taste Receptors and G-Protein α Subunits Expressed in Taste Receptor Cells. J. Neurosci..

[68] Gibbons C., O’Hara B., O’Connor D., Hardman C., Wilton M., Harrold J.A., Almiron-Roig E., Navas-Carretero S., Hodgkins C.E., Nazare J.A. (2022). Acute and Repeated Impact of Sweeteners and Sweetness Enhancers in Solid and Semi-Solid Foods on Appetite: Protocol for a Multicentre, Cross-over, RCT in People with Overweight/Obesity—The SWEET Project. BMJ Open.

[69] Almiron-Roig E., Navas-Carretero S., Castelnuovo G., Kjølbæk L., Romo-Hualde A., Normand M., Maloney N., Hardman C.A., Hodgkins C.E., Moshoyiannis H. (2023). Impact of Acute Consumption of Beverages Containing Plant-Based or Alternative Sweetener Blends on Postprandial Appetite, Food Intake, Metabolism, and Gastro-Intestinal Symptoms: Results of the SWEET Beverages Trial. Appetite.

[70] Gnirke A., Melnikov A., Maguire J., Rogov P., LeProust E.M., Brockman W., Fennell T., Giannoukos G., Fisher S., Russ C. (2009). Solution Hybrid Selection with Ultra-Long Oligonucleotides for Massively Parallel Targeted Sequencing. Nat. Biotechnol..

[71] Adzhubei I.A., Schmidt S., Peshkin L., Ramensky V.E., Gerasimova A., Bork P., Kondrashov A.S., Sunyaev S.R. (2010). A Method and Server for Predicting Damaging Missense Mutations. Nat. Methods.

[72] Bachmanov A.A., Tordoff M.G., Beauchamp G.K. (2001). Sweetener Preference of C57BL/6ByJ and 129P3/J Mice. Chem. Senses.

[73] Reed D.R., Li S., Li X., Huang L., Tordoff M.G., Starling-Roney R., Taniguchi K., West D.B., Ohmen J.D., Beauchamp G.K. (2004). Polymorphisms in the Taste Receptor Gene (*Tas1r3*) Region Are Associated with Saccharin Preference in 30 Mouse Strains. J. Neurosci..

[74] Methven L., Ellis L., Kavaliauskaite G. (2018). Investigating Perception and Liking of Non-Nutritive Sweeteners in Individuals Representing Different Taste Receptor Genotypes. Proceedings of the 15th Weurman Flavour Research Symposium.

[75] Koc G., Soyocak A., Andac-Ozturk S. (2021). TAS1R2 Rs35874116 and TRPM5 Rs886277 Polymorphisms Are Not Related with Risk of Obesity. Int. J. Clin. Pract..

[76] Smith N.J., Grant J.N., Moon J.I., So S.S., Finch A.M. (2021). Critically Evaluating Sweet Taste Receptor Expression and Signaling through a Molecular Pharmacology Lens. FEBS J..

[77] Hwang L.-D., Lin C., Gharahkhani P., Cuellar-Partida G., Ong J.-S., An J., Gordon S.D., Zhu G., MacGregor S., Lawlor D.A. (2019). New Insight into Human Sweet Taste: A Genome-Wide Association Study of the Perception and Intake of Sweet Substances. Am. J. Clin. Nutr..

[78] Diószegi J., Mohammad Kurshed A.A., Pikó P., Kósa Z., Sándor J., Ádány R. (2021). Association of Single Nucleotide Polymorphisms with Taste and Food Preferences of the Hungarian General and Roma Populations. Appetite.

[79] Fernández-Carrión R., Sorlí J.V., Coltell O., Pascual E.C., Ortega-Azorín C., Barragán R., Giménez-Alba I.M., Alvarez-Sala A., Fitó M., Ordovas J.M. (2022). Sweet Taste Preference: Relationships with Other Tastes, Liking for Sugary Foods and Exploratory Genome-Wide Association Analysis in Subjects with Metabolic Syndrome. Biomedicines.

[80] Frayling T.M., Beaumont R.N., Jones S.E., Yaghootkar H., Tuke M.A., Ruth K.S., Casanova F., West B., Locke J., Sharp S. (2018). A Common Allele in FGF21 Associated with Sugar Intake Is Associated with Body Shape, Lower Total Body-Fat Percentage, and Higher Blood Pressure. Cell Rep..

[81] Graham C.A.-M., Spedicati B., Pelliccione G., Gasparini P., Concas M.P. (2023). Regulator of G-Protein Signalling 9: A New Candidate Gene for Sweet Food Liking?. Foods.

[82] Kawafune K., Hachiya T., Nogawa S., Takahashi S., Jia H., Saito K., Kato H. (2020). Strong Association between the 12q24 Locus and Sweet Taste Preference in the Japanese Population Revealed by Genome-Wide Meta-Analysis. J. Hum. Genet..

[83] Kogelman L.J.A., Zhernakova D.V., Westra H.-J., Cirera S., Fredholm M., Franke L., Kadarmideen H.N. (2015). An Integrative Systems Genetics Approach Reveals Potential Causal Genes and Pathways Related to Obesity. Genome Med..

[84] Priego T., Sánchez J., Picó C., Ahrens W., De Henauw S., Kourides Y., Lissner L., Molnár D., Moreno L.A., Russo P. (2015). TAS1R3 and UCN2 Transcript Levels in Blood Cells Are Associated with Sugary and Fatty Food Consumption in Children. J. Clin. Endocrinol. Metab..

[85] Keskitalo K., Knaapila A., Kallela M., Palotie A., Wessman M., Sammalisto S., Peltonen L., Tuorila H., Perola M. (2007). Sweet Taste Preferences Are Partly Genetically Determined: Identification of a Trait Locus on Chromosome 161. Am. J. Clin. Nutr..

[86] Stevens H., Piluso F., Gasparini P., Mavrommatis Y., Pilic L., Graham C.A.-M., Concas M.P. (2024). The Genetics of Sweet Taste: Perception, Feeding Behaviours, and Health. Proceedings.

[87] Bahauddin A.R., Shaari N., Shariff Z., Karim R. (2020). Association Between TAS1R2 Gene Polymorphism (Rs12033832) and Sweet Taste Perception Amongst Malay Obese and Non- Obese Subjects. Malays. J. Med. Health Sci..

