# Supplementary material for: TAS1R2/TAS1R3 Single-Nucleotide Polymorphisms Affect Sweet Taste Receptor Activation by Sweeteners: The SWEET Project
Source: Nutrients. 2025 Mar 8;17(6):949. doi: 10.3390/nu17060949 (PMC11945486; doi:10.3390/nu17060949)
Supplement: Supplementary file 1 [file nutrients-17-00949-s001.zip › nutrients-3480980-supplementary.pdf]

***TAS1R2*/*TAS1R3* single nucleotide polymorphisms affect sweet taste receptor activation by sweeteners: The SWEET Project – Supplementary data**

- **Protein sequence of human TAS1R2-WT**

MGPRAKTICSLFFLLWVLAEPAENSDFYLPGDYLLGGLFSLHANMKGIVHLNFLQVPMCKEYEVKVI
GYNLMQAMRFAVEEINNDSSLLPGVLLGYEIVDVCYISNNVQPVLYFLAHEDNLLPIQEDYSNYISRV
VAVIGPDNSESVMTVANFLSLFLLPQITYSAISDELRDKVRFPALLRTTPSADHHIEAMVQLMLHFRW
NWIIVLVSSDTYGRDNGQLLGERVARRDICIAFQETLPTLQPNQNMTSEERQRLVTIVDKLQQSTARV
VVVFSPDLTLYHFFNEVLRQNFTGAVWIASESWAIDPVLHNLTELRHLGTFLGITIQSVPIPGFSEFRE
WGPQAGPPPLSRTSQSYTCNQECDNCLNATLSFNTILRLSGERVVYSVYSAVYAVAHALHSLLGCDK
STCTKRVVYPWQLLEEIWKVNFTLLDHQIFFDPQGDVALHLEIVQWQWDRSQNPFQSVASYYPLQR
QLKNIQDISWHTINNTIPMSMCSKRCQSGQKKKPVGIHVCCFECIDCLPGTFLNHTEDEYECQACPN
NEWSYQSETSCFKRQLVFLEWHEAPTIAVALLAALGFLSTLAILVIFWRHFQTPIVRSAGGPMCFLML
TLLLVAYMVVPVYVGPPKVSTCLCRQALFPLCFTICISCIAVRSFQIVCAFKMASRFPRAYSYWVRYQG
PYVSMAFITVLKMVIVVIGMLATGLSPTTRTDPDDPKITIVSCNPNYRNSLLFNTSLDLLLSVVGFSFAY
MGKELPTNYNEAKFITLSMTFYFTSSVSLCTFMSAYSGVLVTIVDLLVTVLNLLAISLGYFGPKCYMILF
YPERNTPAYFNSMIQGYTMRRD

- **Protein sequence of human TAS1R3-WT**

MLGPAVLGLSLWALLHPGTGAPLCLSQQLRMKGDYVLGGLFPLGEAEEAGLRSRTRPSSPVCTRFSS
NGLLWALAMKMAVEEINNKSDLLPGLRLGYDLFDTCSEPVVAMKPSLMFLAKAGSRDIAAYCNYT
QYQPRVLAVIGPHSSELAMVTGKFFSFFLMPQVSYGASMELLSARETFPSFFRTVPSDRVQLTAAAELL
QEFGWNWVAALGSDDEYGRQGLSIFSALAAARGICIAHEGLVPLPRADDSRLGKVQDVLHQVNQS
SVQVVLLFASVHAAHALFNYSISSRLSPKVWVASEAWLTSDLVMGLPGMAQMGTVLGFLQRGAQL
HEFPQYVKTHLALATDPAFCSALGEREQGLEEDVVGQRCPQCDCITLQNVSAGLNHHQTFSVYAAV
YSVAQALHNTLQCNASGCPAQDPVKPWQLLENMYNLTFHVGGLPLRFDSSGNVDMEYDLKLWV
WQGSVPRLHDVGRFNGSLRTERLKIRWHTSDNQKPVSRCSRQCQEGQVRRVKGFHSCCYDCVDCE
AGSYRQNPDDIACTFCGQDEWSPERSTRCFRRRSRFLAWGEPAVLLLLLLLSLALGLVLAALGLFVH
HRDSPLVQASGGPLACFGLVCLGLVCLSVLLFPGQPSPARCLAQQPLSHLPLTGCLSTLFLQAAEIFVE
SELPLSWADRLSGCLRGPWAWLVVLLAMLVEVALCTWYLVAFPPEVVTDWHMLPTEALVHCRTRS
WVSFGLAHATNATLAFLCFLGTFLVRSQPGRYNRARGLTFAMLAYFITWVSFVPLLANVQVVLRPAV
QMGALLLCVLGILAAFHLPRCYLLMRQPGLNTPEFFLGGGPGDAQGQNDGNTGNQGKHE

Table S1. *p*-Values calculated for mTAS1R2-WT/mTAS1R3-I60T using ANOVA followed by Dunnett's test of ΔF/F0 for each concentration of sweetener that elicited a response (with reference to mTAS1R2-WT/mTAS1R3-WT). Significative *p*-value are in bold ($p < 0.05$).

Sucralose		AceK		RebA		RebM		Mogroside V		Saccharin	
**Concentrations (μM)**	***p*-values**	**Concentrations (μM)**	***p*-values**	**Concentrations (μM)**	***p*-values**	**Concentrations (μM)**	***p*-values**	**Concentrations (μM)**	***p*-values**	**Concentrations (μM)**	***p*-values**
30	0.549	10	0.453	1	0.691	1	0.800	1	0.474	30	0.901
100	0.994	30	0.294	3	0.647	3	0.647	3	0.565	100	0.527
300	**0.001**	100	**0.001**	10	**0.010**	10	**0.010**	10	0.343	300	0.134
1000	**<0.0001**	300	**<0.001**	30	**<0.001**	30	**<0.001**	30	0.022	1000	**<0.001**
3000	**<0.001**	1000	**0.003**	100	**<0.0001**	100	**<0.0001**	100	**0.012**	3000	**<0.0001**
10000	**0.013**	3000	**0.001**	300	**<0.001**	300	**<0.001**	300	**0.004**	10000	**<0.001**
-	-	-	-	-	-	-	-	1000	0.004	-	-

WT: wild-type; AceK: acesulfame K; RebA: rebaudioside A; RebM: rebaudioside M; NHDC: neohesperidin dihydrochalcone.

Table S2. *p*-Values calculated using ANOVA followed by Dunnett's test for multiple comparison analysis of ΔF/F0 for each sweetener concentration and each TAS1R2-SNP/TAS1R3-WT (with reference to TAS1R2-WT/TAS1R3-WT). Significative *p*-value are in bold ($p < 0.05$).

	**Concentrations**	**C9S**	**P21L**	**I191V**	**S212G**	**I234V**	**R317G**	**R317P**	**I486V**	**A574T**	**I595T**	**K689Q**	**R838K**
**Sucralose (μM)**	**3**	0.991	0.999	1.000	0.982	0.736	1.000	0.372	0.936	1.000	0.235	0.982	1.000
	**10**	0.998	1.000	0.999	1.000	0.905	1.000	0.518	1.000	1.000	0.426	0.800	1.000
	**30**	0.896	1.000	1.000	1.000	0.296	**0.029**	1.000	0.482	0.987	0.225	0.139	0.791
	**100**	0.981	1.000	1.000	1.000	0.054	0.068	0.980	0.084	1.000	**0.002**	**0.001**	0.808
	**300**	0.978	1.000	1.000	0.992	0.195	0.266	0.916	0.371	0.999	**<0.0001**	**<0.0001**	0.519
	**1000**	0.864	0.999	1.000	1.000	0.309	0.142	0.564	0.840	1.000	**<0.0001**	**<0.0001**	0.519
**Neotame (μM)**	**0.03**	0.410	1.000	1.000	1.000	0.493	0.799	1.000	0.992	0.987	1.000	1.000	0.999
	**0.1**	0.489	1.000	0.979	1.000	1.000	1.000	0.313	1.000	0.691	0.248	0.522	0.530
	**0.3**	1.000	1.000	0.996	1.000	1.000	1.000	0.896	1.000	1.000	0.300	0.470	0.178
	**1**	0.999	1.000	0.995	1.000	0.704	0.997	0.976	0.445	0.992	**<0.001**	**<0.0001**	0.064
	**3**	0.857	1.000	0.998	1.000	0.454	1.000	1.000	0.990	0.996	**<0.0001**	**<0.0001**	0.208
	**10**	0.854	1.000	0.998	1.000	0.924	1.000	0.999	1.000	1.000	**<0.0001**	**<0.0001**	0.312
	**30**	0.996	0.983	0.926	0.961	0.848	1.000	0.810	0.807	0.958	**<0.0001**	**<0.0001**	**0.033**
**AceK (μM)**	**10**	1.000	1.000	1.000	0.474	1.000	0.997	0.879	0.999	1.000	1.000	0.875	1.000
	**30**	1.000	1.000	0.661	1.000	1.000	0.547	1.000	1.000	0.990	0.085	1.000	0.970
	**100**	0.954	1.000	0.558	1.000	0.981	0.999	0.698	1.000	1.000	0.203	0.098	1.000
	**300**	0.817	0.997	1.000	0.998	0.254	0.057	1.000	0.849	1.000	0.229	0.157	0.267
	**1000**	0.962	1.000	1.000	0.984	0.449	0.076	0.762	0.923	1.000	**0.018**	**0.005**	0.146
	**3000**	0.998	1.000	1.000	1.000	0.561	0.119	0.509	0.584	1.000	**0.001**	**0.001**	0.371
**RebA (μM)**	**1**	0.952	0.548	0.911	0.124	1.000	1.000	1.000	0.997	0.980	0.165	0.984	1.000
	**3**	0.965	0.735	0.896	1.000	1.000	1.000	0.373	1.000	0.988	0.373	0.119	1.000
	**10**	0.996	0.969	0.843	0.989	0.758	0.954	**0.043**	1.000	1.000	**0.001**	**<0.001**	1.000
	**30**	1.000	1.000	1.000	1.000	1.000	0.998	0.758	1.000	1.000	**<0.0001**	**<0.0001**	1.000
	**100**	0.979	1.000	1.000	0.999	1.000	1.000	1.000	0.999	1.000	**<0.0001**	**<0.0001**	1.000
	**300**	0.996	1.000	0.997	1.000	1.000	1.000	1.000	1.000	0.999	**<0.0001**	**<0.0001**	1.000
**RebM (μM)**	**1**	0.794	1.000	1.000	1.000	0.997	1.000	0.492	0.992	0.539	0.448	0.088	0.962
	**3**	0.974	0.315	0.738	0.523	1.000	1.000	0.365	0.998	0.290	**0.003**	**0.001**	0.537
	**10**	0.969	0.785	0.855	0.892	0.977	1.000	0.161	1.000	1.000	**<0.001**	**<0.0001**	1.000
	**30**	1.000	0.987	1.000	0.996	1.000	1.000	0.965	0.998	1.000	**<0.0001**	**<0.0001**	1.000
	**100**	1.000	1.000	1.000	1.000	0.996	0.990	1.000	0.977	1.000	**<0.0001**	**<0.0001**	1.000
	**300**	1.000	0.990	1.000	1.000	1.000	0.989	1.000	0.997	1.000	**<0.0001**	**<0.0001**	1.000
**Mogroside V (μM)**	**1**	1.000	1.000	1.000	0.971	1.000	1.000	0.962	1.000	1.000	**0.039**	0.134	1.000
	**3**	1.000	1.000	1.000	0.808	0.994	0.938	0.209	1.000	0.998	**0.035**	**0.015**	0.986
	**10**	1.000	0.998	1.000	1.000	0.996	1.000	0.692	1.000	1.000	**0.002**	**0.001**	1.000
	**30**	1.000	0.999	1.000	1.000	1.000	1.000	1.000	1.000	1.000	**<0.0001**	**<0.0001**	1.000
	**100**	1.000	0.996	1.000	1.000	1.000	1.000	1.000	1.000	1.000	**<0.0001**	**<0.001**	1.000
	**300**	1.000	1.000	1.000	1.000	1.000	1.000	0.970	1.000	1.000	**<0.0001**	**0.001**	1.000
**Saccharin (μM)**	**3**	0.521	**0.003**	0.629	0.711	0.597	**0.011**	0.154	**0.016**	0.979	0.906	1.000	1.000
	**10**	1.000	0.194	0.341	0.844	0.128	0.502	**0.019**	0.057	1.000	1.000	1.000	0.999
	**30**	1.000	0.966	1.000	1.000	0.998	**0.033**	0.997	0.637	1.000	0.947	0.525	1.000
	**100**	0.993	0.866	0.258	0.998	1.000	**0.002**	0.169	0.980	1.000	**0.030**	**0.008**	1.000
	**300**	0.368	0.136	0.100	0.702	1.000	**0.003**	**0.015**	1.000	1.000	**<0.0001**	**<0.0001**	0.997
	**1000**	0.650	0.182	**0.021**	0.636	1.000	**0.009**	**0.046**	0.999	0.991	**<0.0001**	**<0.0001**	1.000
**Cyclamate (μM)**	**10**	1.000	0.968	1.000	1.000	0.767	0.952	1.000	1.000	0.635	0.631	1.000	0.897
	**30**	1.000	0.998	0.760	0.806	0.972	1.000	1.000	1.000	0.975	1.000	0.995	1.000
	**100**	0.956	0.297	0.935	1.000	1.000	1.000	1.000	0.989	0.999	1.000	1.000	1.000
	**300**	0.938	0.977	0.978	1.000	0.998	1.000	1.000	0.665	0.932	1.000	0.997	1.000

	**1000**	1.000	1.000	1.000	0.999	0.974	0.994	0.513	0.532	0.999	**0.014**	**0.010**	0.857
	**3000**	1.000	1.000	1.000	0.999	1.000	0.868	0.151	0.815	0.996	**<0.0001**	**<0.0001**	0.721
	**10000**	1.000	1.000	1.000	1.000	0.754	0.133	0.988	0.490	1.000	**<0.0001**	**<0.001**	0.924
**NHDC (μM)**	**1**	0.812	0.943	1.000	0.990	1.000	0.887	1.000	1.000	1.000	0.992	0.994	0.994
	**3**	1.000	0.998	0.945	1.000	1.000	1.000	0.849	1.000	1.000	0.285	0.577	1.000
	**10**	0.989	1.000	0.999	1.000	1.000	0.992	0.305	1.000	1.000	**0.049**	0.116	1.000
	**30**	0.997	1.000	1.000	1.000	1.000	1.000	0.163	0.993	0.992	**0.021**	**0.006**	1.000
	**100**	1.000	1.000	0.995	0.997	1.000	1.000	0.088	0.986	0.999	**<0.0001**	**<0.0001**	1.000
	**300**	1.000	1.000	1.000	1.000	1.000	1.000	0.077	0.930	0.909	**<0.0001**	**<0.0001**	0.916
	**1000**	0.999	1.000	1.000	0.967	1.000	0.989	0.807	0.611	0.911	**<0.0001**	**<0.0001**	0.992
**Thaum atin (μM)**	**0.1**	0.999	0.531	0.882	0.169	0.102	**0.011**	0.782	0.215	0.984	0.962	0.889	0.317
	**0.3**	1.000	1.000	1.000	1.000	0.982	**0.048**	0.931	0.873	1.000	1.000	0.999	1.000
	**1**	0.987	0.617	0.920	1.000	1.000	1.000	1.000	0.923	1.000	1.000	1.000	1.000
	**3**	1.000	0.973	0.612	1.000	1.000	1.000	0.230	0.890	1.000	0.307	0.160	0.931
	**10**	1.000	0.426	0.447	1.000	1.000	0.991	**0.001**	0.885	1.000	**<0.001**	**<0.001**	0.813
	**30**	0.101	0.997	1.000	0.114	0.815	0.373	**0.018**	0.505	0.996	**<0.0001**	**0.001**	0.893
**Brazze in (g/L)**	**0.01**	1.000	1.000	0.897	0.465	**0.028**	0.055	0.330	**0.006**	0.966	1.000	0.999	0.900
	**0.03**	0.943	0.572	0.355	0.770	0.498	0.992	**0.023**	1.000	0.980	**0.002**	**0.008**	1.000
	**0.1**	0.943	0.068	**0.002**	0.108	0.987	0.739	**<0.0001**	1.000	0.623	**<0.0001**	**<0.0001**	0.997
	**0.3**	0.987	0.794	0.139	1.000	0.597	0.983	**<0.0001**	1.000	1.000	**<0.0001**	**<0.0001**	1.000
	**1**	1.000	0.623	0.943	1.000	0.100	1.000	**0.001**	0.999	0.999	**<0.0001**	**<0.0001**	1.000
	**3**	1.000	0.557	1.000	1.000	0.589	0.999	0.415	0.996	1.000	**<0.0001**	**<0.0001**	1.000
**Perilla rtine (TAS1 R2/ TAS1 R3) (μM)**	**0.3**	1.000	0.949	0.956	0.901	0.996	**0.047**	1.000	0.839	**0.012**	0.996	0.997	0.285
	**1**	0.691	0.998	0.979	1.000	**0.003**	0.177	0.316	0.847	0.077	0.986	0.804	0.137
	**3**	0.207	0.691	0.099	0.621	0.998	1.000	0.707	1.000	1.000	0.164	0.437	1.000
	**10**	0.623	0.108	0.058	0.326	1.000	1.000	**0.024**	1.000	0.993	**0.003**	**0.002**	0.998
	**30**	0.374	**0.006**	**0.001**	0.107	1.000	1.000	**<0.0001**	1.000	0.996	**<0.0001**	**<0.0001**	1.000
	**100**	0.053	0.264	0.060	**0.046**	1.000	1.000	**<0.0001**	1.000	0.859	**<0.0001**	**<0.0001**	0.999
	**300**	1.000	0.997	0.998	1.000	0.924	0.728	0.719	0.996	1.000	**<0.0001**	0.102	1.000
**Perilla rtine (TAS1 R2) (μM)**	**3**	0.935	1.000	0.992	1.000	0.999	0.348	1.000	1.000	0.576	0.310	0.848	0.733
	**10**	0.980	0.551	0.970	0.990	0.956	1.000	0.890	0.955	0.666	0.786	0.567	0.194
	**30**	0.082	0.058	**0.012**	0.060	**0.009**	**0.016**	**0.009**	**0.026**	**0.016**	**0.009**	**0.007**	**0.035**
	**100**	**<0.0001**	**<0.0001**	**<0.0001**	**<0.0001**	**<0.0001**	**<0.0001**	**<0.0001**	**0.000**	**0.000**	**<0.0001**	**<0.0001**	**0.002**
	**300**	**<0.0001**	**<0.0001**	**<0.0001**	**<0.0001**	**<0.0001**	**<0.0001**	**<0.0001**	**<0.0001**	**<0.0001**	**<0.0001**	**<0.0001**	**<0.0001**
	**1000**	**<0.0001**	**<0.0001**	**<0.0001**	**<0.0001**	**<0.0001**	**<0.0001**	**<0.0001**	**<0.0001**	**<0.0001**	**<0.0001**	**<0.0001**	**<0.0001**
	**3000**	**<0.0001**	**<0.0001**	**<0.0001**	**<0.0001**	**<0.0001**	**<0.0001**	**<0.0001**	**<0.0001**	**<0.0001**	**<0.0001**	**<0.0001**	**<0.0001**

WT: wild-type; AceK: acesulfame K; RebA: rebaudioside A; RebM: rebaudioside M; NHDC: neohesperidin dihydrochalcone.

Table S3. *p*-Values calculated using ANOVA followed by Dunnett's test for multiple comparison analysis of $\Delta F/F0$ for each sweetener concentration and each TAS1R2-WT/TAS1R3-SNP (with reference to TAS1R2-WT/TAS1R3-WT). Significative *p*-value are in bold ($p < 0.05$).

	**Concentrations**	**A5T**	**L95P**	**M110T**	**R247H**	**G367C**	**S446N**	**F514L**	**S551N**	**T716M**	**A735T**	**F749S**	**C757R**	**P780A**	**R813K**	**L823F**	**G832R**
**Sucralose (μM)**	**3**	0.999	1.000	0.985	1.000	1.000	1.000	1.000	1.000	1.000	1.000	1.000	1.000	1.000	0.588	0.906	1.000
	**10**	1.000	0.983	1.000	0.945	1.000	1.000	1.000	0.997	0.461	1.000	1.000	0.930	0.241	**0.030**	0.981	**0.004**
	**30**	0.955	**0.028**	0.448	1.000	0.070	0.799	0.863	**0.013**	0.599	0.999	**0.048**	0.985	1.000	0.990	1.000	**0.002**
	**100**	0.999	**<0.001**	**0.001**	1.000	**0.012**	1.000	0.637	**<0.001**	0.419	0.998	**0.002**	0.829	1.000	0.580	1.000	0.069
	**300**	1.000	**<0.0001**	**<0.0001**	1.000	**0.036**	1.000	0.826	**<0.0001**	0.610	1.000	**<0.001**	0.975	1.000	0.657	0.995	0.104
	**1000**	1.000	**<0.0001**	**0.001**	1.000	0.107	1.000	0.941	**<0.0001**	0.879	1.000	**0.002**	0.859	0.999	0.308	0.867	0.233
**Neotame (μM)**	**0.03**	1.000	0.751	1.000	1.000	0.968	0.834	1.000	0.643	0.493	0.312	0.991	0.820	0.534	0.681	1.000	0.876
	**0.1**	1.000	0.098	0.749	0.998	0.999	1.000	0.927	1.000	0.999	1.000	0.999	1.000	1.000	1.000	0.987	**0.002**
	**0.3**	1.000	0.560	0.644	1.000	0.814	1.000	1.000	0.537	1.000	1.000	0.905	0.264	0.990	0.995	1.000	**<0.0001**
	**1**	1.000	**<0.001**	**0.004**	1.000	0.055	1.000	0.986	**<0.001**	1.000	0.980	**0.018**	0.215	1.000	0.366	0.980	**0.005**
	**3**	1.000	**<0.0001**	**<0.0001**	1.000	**0.007**	1.000	0.388	**<0.0001**	1.000	1.000	**0.001**	0.500	1.000	0.429	0.968	0.191
	**10**	1.000	**<0.0001**	**<0.001**	1.000	**0.011**	1.000	0.390	**<0.0001**	0.988	1.000	**0.002**	0.957	1.000	0.984	1.000	0.630
	**30**	1.000	**<0.0001**	**0.003**	1.000	0.125	1.000	0.815	**<0.0001**	1.000	1.000	**0.012**	1.000	1.000	0.968	0.993	0.460
**AceK (μM)**	**10**	1.000	1.000	1.000	0.999	1.000	0.999	1.000	1.000	0.954	1.000	0.900	1.000	0.974	0.896	1.000	0.983
	**30**	0.930	0.999	1.000	1.000	0.965	0.483	0.997	0.992	1.000	0.994	1.000	0.991	1.000	1.000	1.000	0.856
	**100**	0.959	0.536	0.530	1.000	0.139	0.744	0.527	0.622	1.000	1.000	0.998	1.000	0.997	0.854	1.000	**0.008**
	**300**	0.998	0.067	0.071	0.998	0.231	1.000	0.960	0.057	1.000	0.679	0.522	0.100	0.314	**0.001**	**0.020**	**<0.0001**
	**1000**	1.000	**<0.0001**	**<0.001**	0.999	**0.008**	0.983	0.802	**<0.001**	1.000	0.126	**0.002**	**0.009**	0.672	**0.009**	0.144	**<0.0001**
	**3000**	1.000	**<0.0001**	**<0.0001**	0.968	**0.001**	0.677	0.178	**<0.0001**	1.000	**0.009**	**<0.001**	**0.048**	0.999	0.071	0.886	**<0.0001**
**RebA (μM)**	**1**	0.972	0.779	0.951	0.999	1.000	0.996	1.000	1.000	1.000	1.000	1.000	0.977	1.000	0.999	1.000	0.526
	**3**	0.999	0.984	0.926	1.000	1.000	1.000	1.000	1.000	0.970	0.904	1.000	0.998	0.490	0.990	1.000	**<0.0001**
	**10**	1.000	0.986	0.969	0.995	1.000	1.000	1.000	0.996	0.301	**0.002**	1.000	0.154	**<0.0001**	0.077	**0.001**	**<0.0001**
	**30**	0.309	0.337	0.575	0.131	0.938	1.000	1.000	0.400	**0.017**	**<0.0001**	1.000	**<0.001**	**<0.0001**	**0.019**	**<0.001**	**<0.0001**
	**100**	0.349	**0.016**	0.222	0.104	0.518	1.000	1.000	**0.027**	**0.003**	**<0.0001**	0.988	**0.001**	**<0.001**	**0.021**	**<0.001**	**<0.0001**
	**300**	0.264	**0.008**	0.320	0.157	0.639	1.000	1.000	**0.011**	**0.011**	**0.001**	0.998	**0.021**	**0.019**	**0.047**	**0.005**	**<0.001**
**Reb M (μM)**	**1**	0.769	0.834	0.988	0.942	1.000	1.000	0.999	1.000	1.000	1.000	1.000	1.000	0.195	0.418	0.457	**0.013**
	**3**	0.967	0.330	0.582	1.000	0.996	1.000	1.000	1.000	0.981	0.473	1.000	0.997	0.032	0.116	0.298	**<0.0001**
	**10**	0.992	0.652	0.747	0.324	0.959	1.000	1.000	0.755	0.361	**0.001**	0.999	**0.020**	**0.001**	**0.017**	**0.005**	**<0.0001**
	**30**	0.392	0.080	0.320	0.098	0.740	1.000	1.000	0.164	0.114	**0.001**	0.942	**0.015**	**<0.001**	**0.013**	**0.010**	**<0.0001**
	**100**	0.113	**0.007**	0.254	0.115	0.671	1.000	1.000	**0.011**	0.275	**0.008**	0.934	0.063	**0.008**	**0.028**	**0.004**	**0.001**
	**300**	0.351	**0.004**	0.544	0.127	0.637	1.000	1.000	**0.007**	0.228	**0.024**	0.986	0.140	**0.032**	**0.099**	**0.007**	**0.001**
**Mogroside V (μM)**	**1**	0.984	0.439	0.632	0.999	0.881	0.460	1.000	0.997	0.782	0.998	0.996	1.000	0.860	0.999	1.000	0.689
	**3**	0.146	0.164	**0.005**	0.971	0.947	0.678	1.000	0.295	0.992	1.000	0.682	0.596	0.601	0.267	0.398	**<0.001**
	**10**	0.960	**<0.0001**	**<0.0001**	0.963	**0.003**	0.263	0.861	**<0.001**	1.000	0.402	**0.001**	0.060	**0.002**	**0.002**	**0.008**	**<0.0001**
	**30**	1.000	**<0.0001**	**<0.0001**	0.689	**<0.0001**	0.136	0.266	**<0.0001**	1.000	0.053	**<0.0001**	**0.006**	**0.001**	**0.001**	**<0.0001**	**<0.0001**
	**100**	0.861	**<0.0001**	**<0.0001**	0.065	**<0.0001**	0.414	0.097	**<0.0001**	0.994	0.090	**<0.0001**	0.310	0.212	0.055	**0.016**	**<0.0001**
	**300**	0.995	**<0.0001**	**<0.0001**	0.118	**<0.0001**	0.874	0.245	**<0.0001**	0.945	**0.006**	**<0.0001**	0.409	**0.039**	**0.023**	**0.002**	**<0.001**
**Saccharin (μM)**	**3**	0.999	0.733	0.867	0.997	1.000	0.999	1.000	1.000	1.000	1.000	1.000	1.000	1.000	0.967	0.998	1.000
	**10**	0.371	0.270	**0.036**	1.000	0.465	0.886	0.994	0.195	0.767	1.000	0.974	0.665	1.000	1.000	1.000	1.000
	**30**	0.433	**0.020**	0.179	0.986	0.414	1.000	1.000	0.310	0.965	1.000	0.609	1.000	0.914	**0.017**	0.742	**0.003**
	**100**	0.998	**0.001**	**0.003**	1.000	0.242	0.997	1.000	**0.007**	0.968	1.000	**0.033**	0.940	0.399	**0.026**	0.161	**0.001**
	**300**	1.000	**<0.0001**	**<0.0001**	1.000	**0.015**	1.000	0.971	**<0.0001**	0.967	1.000	**<0.001**	0.915	0.999	0.128	0.147	**0.039**
	**1000**	1.000	**<0.0001**	**<0.0001**	1.000	0.079	1.000	0.690	**<0.0001**	0.968	0.997	**0.003**	1.000	0.997	0.944	0.942	0.224
**Cyclamate (μM)**	**10**	0.985	0.500	0.546	0.722	1.000	1.000	1.000	1.000	0.990	1.000	1.000	1.000	1.000	1.000	1.000	1.000
	**30**	0.922	0.715	0.836	0.653	1.000	1.000	1.000	0.810	1.000	1.000	1.000	1.000	0.949	1.000	1.000	1.000
	**100**	0.138	**0.033**	0.223	0.860	0.971	0.995	1.000	0.597	0.996	0.832	0.984	1.000	0.758	1.000	1.000	1.000
	**300**	0.246	**0.007**	**0.002**	0.384	**0.031**	1.000	0.995	**0.030**	0.238	0.651	**0.020**	1.000	1.000	1.000	1.000	0.939
	**1000**	0.733	**<0.0001**	**<0.0001**	0.929	**0.026**	1.000	0.932	**<0.0001**	0.117	0.413	**<0.0001**	0.997	1.000	1.000	1.000	**0.048**
	**3000**	1.000	**<0.0001**	**<0.0001**	1.000	0.081	1.000	1.000	**<0.0001**	0.734	1.000	**<0.0001**	1.000	1.000	1.000	1.000	0.898

	**10000**	1.000	**<0.0001**	**<0.0001**	0.994	0.232	1.000	1.000	**<0.0001**	0.794	1.000	**<0.0001**	0.999	0.863	1.000	1.000	1.000
**NHDC (μM)**	**1**	0.438	**0.001**	**0.018**	0.931	**0.001**	0.681	0.865	**0.001**	**0.002**	0.998	**<0.001**	1.000	0.068	1.000	0.846	1.000
	**3**	**0.022**	**<0.0001**	**<0.0001**	0.385	**0.006**	0.966	1.000	**<0.0001**	**0.016**	0.166	**0.001**	1.000	0.094	0.987	0.991	0.998
	**10**	0.612	**<0.0001**	**<0.001**	0.318	**0.007**	0.935	0.920	**<0.001**	0.231	0.977	**<0.001**	1.000	0.985	1.000	1.000	0.804
	**30**	0.989	**<0.0001**	**<0.0001**	0.999	**0.004**	1.000	0.403	**<0.0001**	0.365	1.000	**<0.0001**	1.000	0.971	0.978	1.000	0.102
	**100**	0.962	**<0.0001**	**<0.0001**	0.999	**0.003**	1.000	0.981	**<0.0001**	0.478	1.000	**<0.0001**	0.999	**0.003**	0.998	1.000	0.522
	**300**	1.000	**<0.0001**	**<0.0001**	1.000	**0.027**	1.000	1.000	**<0.0001**	0.771	1.000	**<0.0001**	1.000	**0.002**	1.000	1.000	1.000
	**1000**	0.999	**<0.0001**	**<0.0001**	0.955	**0.040**	0.984	1.000	**<0.0001**	0.974	1.000	**0.001**	0.987	**0.021**	1.000	1.000	1.000
**Thaumatin (μM)**	**0.1**	1.000	1.000	1.000	0.998	1.000	1.000	1.000	0.996	1.000	1.000	1.000	1.000	1.000	0.963	1.000	0.993
	**0.3**	1.000	0.691	1.000	0.983	1.000	1.000	1.000	1.000	0.985	0.991	1.000	1.000	0.827	1.000	1.000	0.717
	**1**	1.000	1.000	1.000	1.000	1.000	0.690	0.978	0.997	1.000	0.118	1.000	0.988	**0.042**	0.207	0.595	0.103
	**3**	0.991	0.604	0.726	0.521	1.000	0.429	0.489	1.000	0.972	**0.049**	1.000	0.142	0.066	**0.017**	0.124	**<0.001**
	**10**	0.833	**0.019**	0.210	0.332	0.933	0.976	0.951	0.369	0.815	0.091	0.130	0.101	**0.013**	**0.025**	0.395	**0.003**
	**30**	0.907	**0.001**	0.476	0.462	0.996	0.993	0.978	**0.041**	0.778	0.626	0.069	0.895	0.138	0.819	0.983	0.777
	**100**	0.947	**0.039**	0.998	1.000	1.000	1.000	1.000	0.180	0.990	1.000	0.813	1.000	0.809	0.996	1.000	0.986
**Brazzein (g/L)**	**0.01**	1.000	1.000	1.000	0.998	1.000	1.000	**0.001**	0.999	1.000	0.877	1.000	0.708	1.000	0.993	0.997	0.490
	**0.03**	0.999	0.783	0.920	0.689	0.992	1.000	**<0.0001**	0.683	1.000	0.448	0.985	0.351	0.924	**0.022**	0.786	**0.002**
	**0.1**	0.999	**0.007**	0.216	0.218	0.669	1.000	**0.001**	**0.009**	1.000	0.846	0.203	0.781	0.186	**0.043**	0.484	**0.021**
	**0.3**	1.000	**<0.0001**	0.184	0.972	0.365	1.000	0.428	**<0.0001**	1.000	0.999	0.193	1.000	0.430	0.852	0.892	0.515
	**1**	0.995	**<0.0001**	0.733	1.000	0.828	1.000	0.998	**<0.0001**	1.000	1.000	0.604	1.000	0.993	1.000	1.000	1.000
	**3**	0.998	**<0.0001**	0.992	1.000	0.998	1.000	0.985	**<0.001**	1.000	1.000	0.967	1.000	1.000	1.000	1.000	1.000
**Perillartine (μM)**	**0.3**	0.731	0.341	1.000	1.000	0.924	0.915	0.983	0.928	0.999	1.000	1.000	1.000	1.000	0.994	1.000	0.996
	**1**	0.969	0.506	0.599	0.891	0.724	0.894	0.677	0.519	0.999	1.000	0.883	1.000	0.873	0.912	0.966	0.842
	**3**	0.961	**<0.0001**	**<0.0001**	0.909	**<0.001**	**0.02**5	0.173	**0.001**	0.194	0.988	0.454	1.000	0.990	1.000	0.297	1.000
	**10**	1.000	**0.031**	0.103	1.000	0.964	1.000	1.000	**0.022**	1.000	1.000	0.871	1.000	1.000	1.000	1.000	1.000
	**30**	1.000	**<0.0001**	**<0.0001**	1.000	**0.005**	0.988	1.000	**<0.0001**	0.984	1.000	0.929	1.000	1.000	1.000	0.822	1.000
	**100**	1.000	**<0.0001**	0.278	0.995	1.000	1.000	1.000	**<0.0001**	1.000	1.000	0.366	0.985	1.000	0.995	1.000	0.969
	**300**	0.973	**<0.0001**	0.521	0.600	1.000	0.991	0.879	**<0.0001**	0.885	0.999	**0.072**	0.538	0.997	0.391	0.985	0.132

WT: wild-type; AceK: acesulfame K; RebA: rebaudioside A; RebM: rebaudioside M; NHDC: neohesperidin dihydrochalcone.

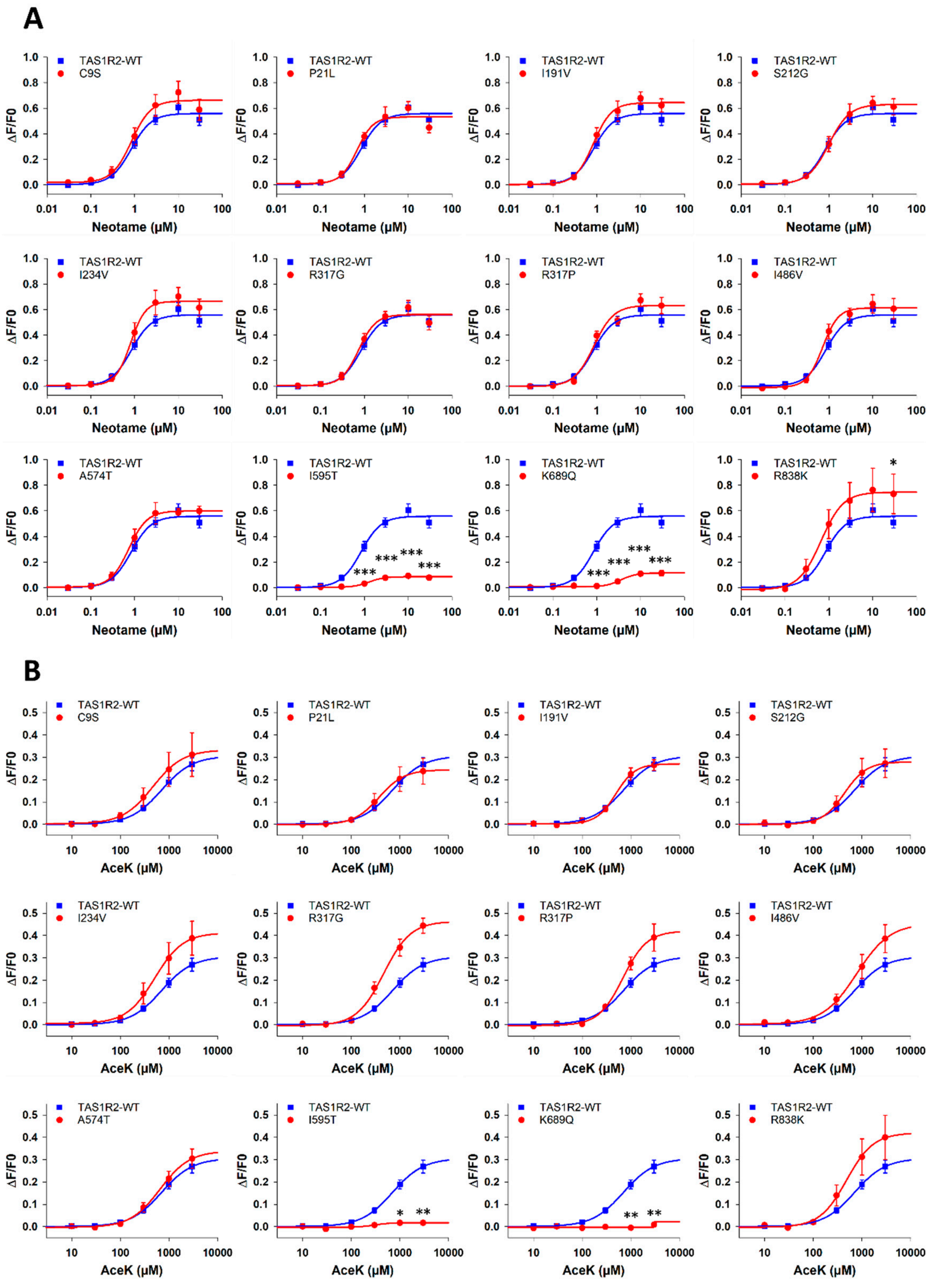

A
TAS1R2-WT
C9S
P21L
I191V
S212G
I234V
R317G
R317P
I486V
A574T
I595T
K689Q
R838K
ΔF/F0
Neotame (μM)
***
*
B
AceK (μM)
**

C

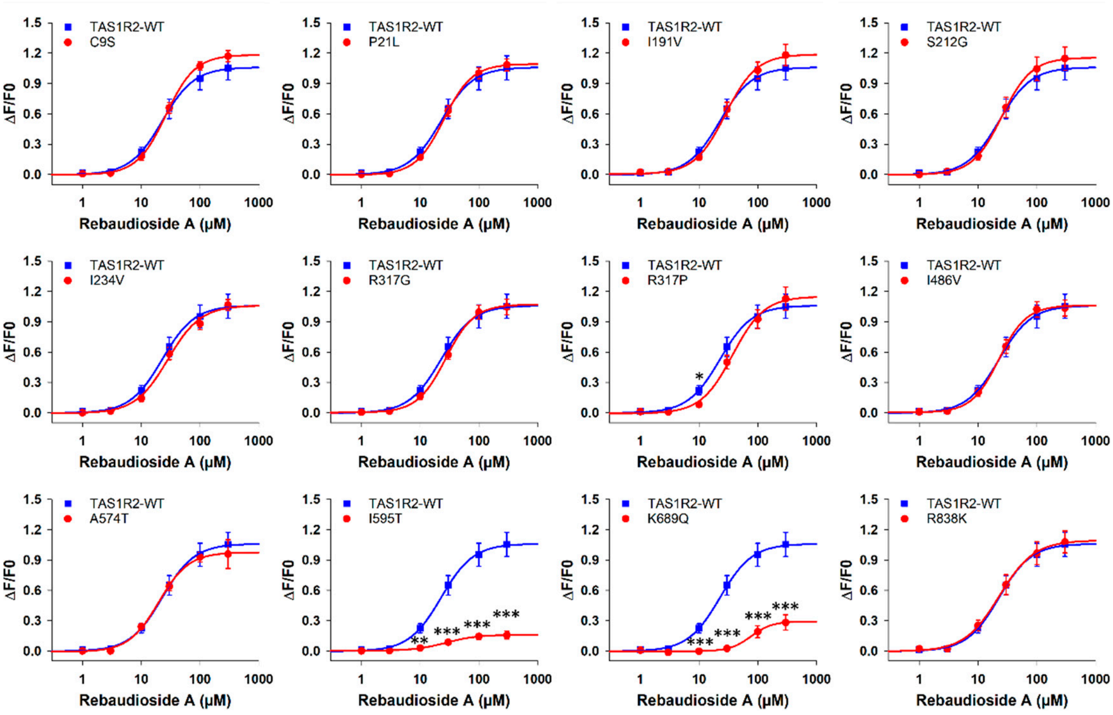
TAS1R2-WT
C9S
TAS1R2-WT
P21L
TAS1R2-WT
I191V
TAS1R2-WT
S212G
TAS1R2-WT
I234V
TAS1R2-WT
R317G
TAS1R2-WT
R317P
TAS1R2-WT
I486V
TAS1R2-WT
A574T
TAS1R2-WT
I595T
TAS1R2-WT
K689Q
TAS1R2-WT
R838K
ΔF/F0
Rebaudioside A (μM)
1.5
1.2
0.9
0.6
0.3
0.0
1
10
100
1000
*
**
***

D

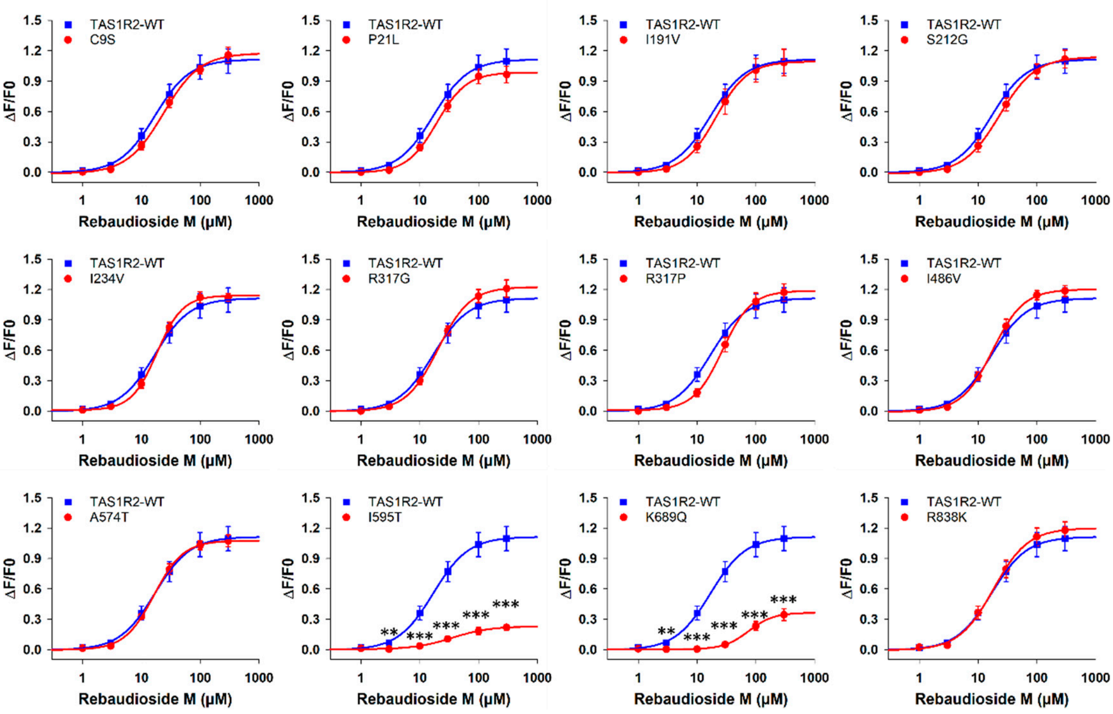
TAS1R2-WT
C9S
TAS1R2-WT
P21L
TAS1R2-WT
I191V
TAS1R2-WT
S212G
TAS1R2-WT
I234V
TAS1R2-WT
R317G
TAS1R2-WT
R317P
TAS1R2-WT
I486V
TAS1R2-WT
A574T
TAS1R2-WT
I595T
TAS1R2-WT
K689Q
TAS1R2-WT
R838K
ΔF/F0
Rebaudioside M (μM)
1.5
1.2
0.9
0.6
0.3
0.0
1
10
100
1000
**
***

E

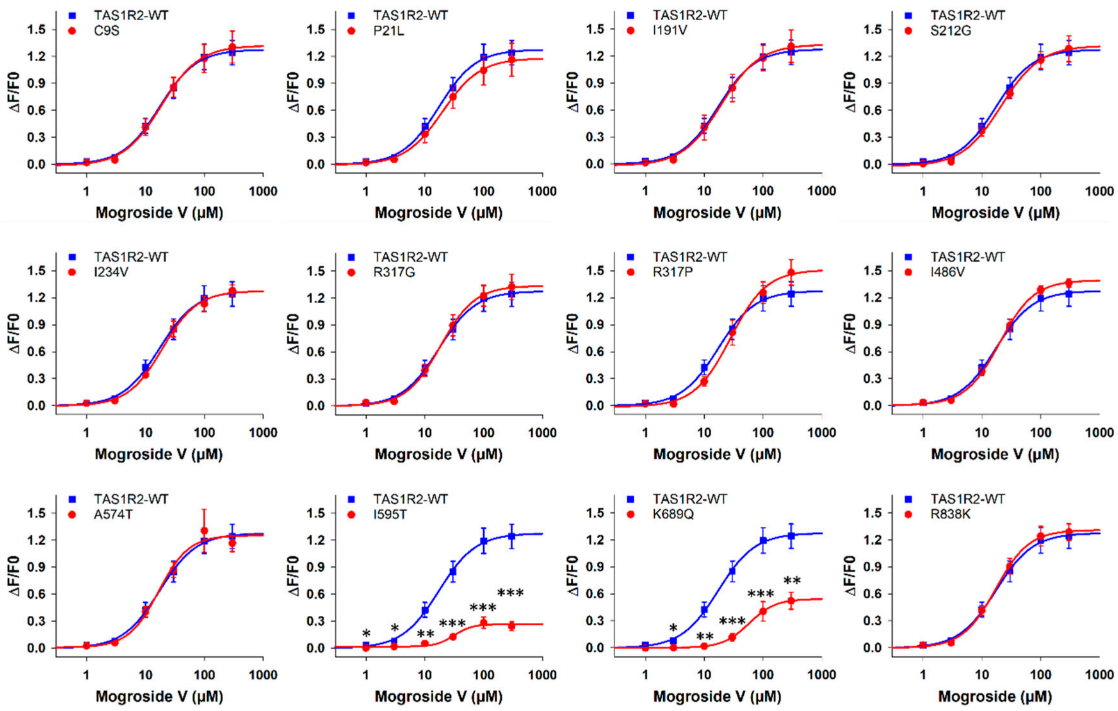
TAS1R2-WT
C9S
P21L
I191V
S212G
I234V
R317G
R317P
I486V
A574T
I595T
K689Q
R838K
ΔF/F0
Mogroside V (μM)
Mogroside (μM)

F

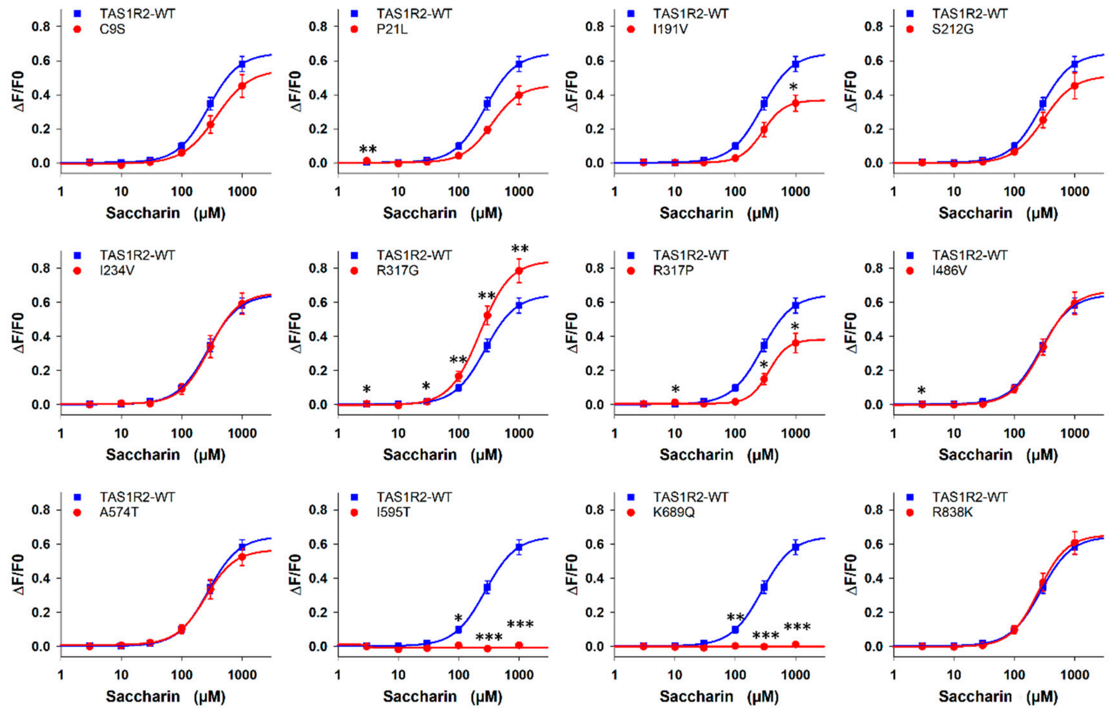
TAS1R2-WT
C9S
P21L
I191V
S212G
I234V
R317G
R317P
I486V
A574T
I595T
K689Q
R838K
ΔF/F0
Saccharin (μM)

G

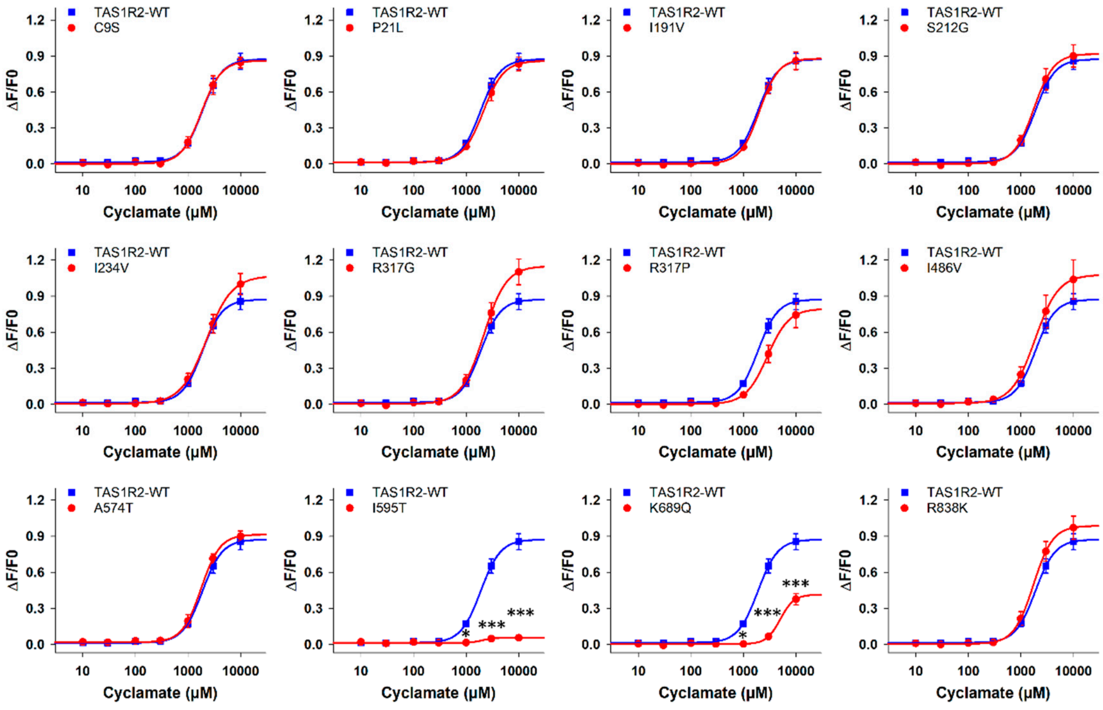

TAS1R2-WT
C9S
TAS1R2-WT
P21L
TAS1R2-WT
I191V
TAS1R2-WT
S212G
TAS1R2-WT
I234V
TAS1R2-WT
R317G
TAS1R2-WT
R317P
TAS1R2-WT
I486V
TAS1R2-WT
A574T
TAS1R2-WT
I595T
TAS1R2-WT
K689Q
TAS1R2-WT
R838K
ΔF/F0
0.0
0.3
0.6
0.9
1.2
10
100
1000
10000
Cyclamate (μM)
*
***

H

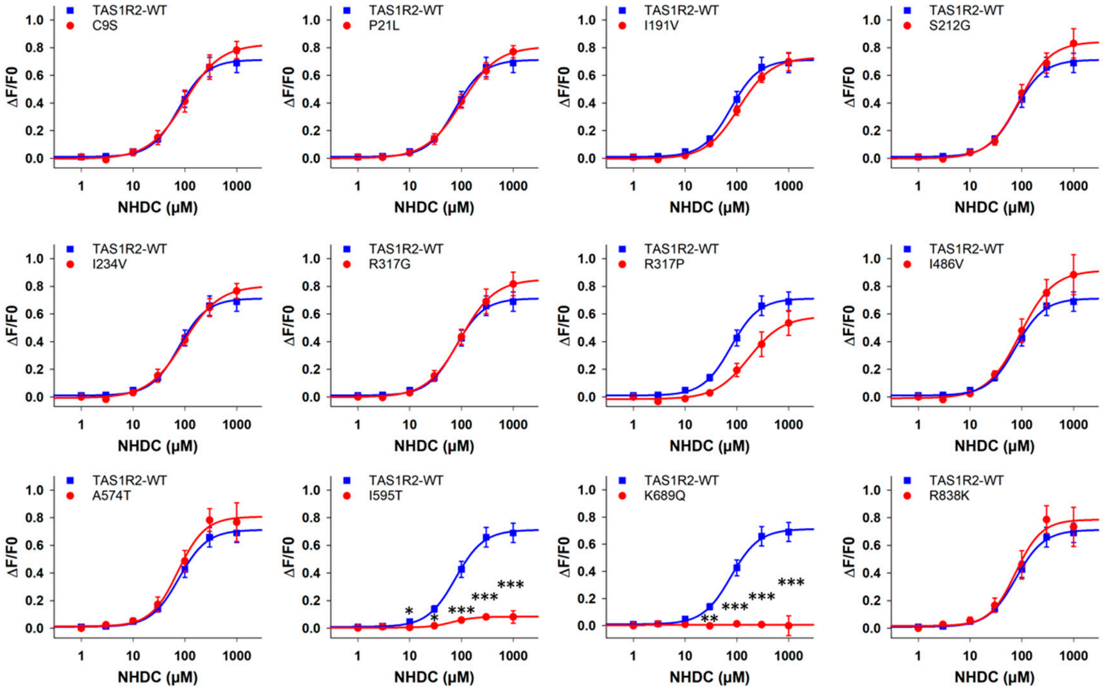

TAS1R2-WT
C9S
TAS1R2-WT
P21L
TAS1R2-WT
I191V
TAS1R2-WT
S212G
TAS1R2-WT
I234V
TAS1R2-WT
R317G
TAS1R2-WT
R317P
TAS1R2-WT
I486V
TAS1R2-WT
A574T
TAS1R2-WT
I595T
TAS1R2-WT
K689Q
TAS1R2-WT
R838K
ΔF/F0
0.0
0.2
0.4
0.6
0.8
1.0
1
10
100
1000
NHDC (μM)
*
**
***

I

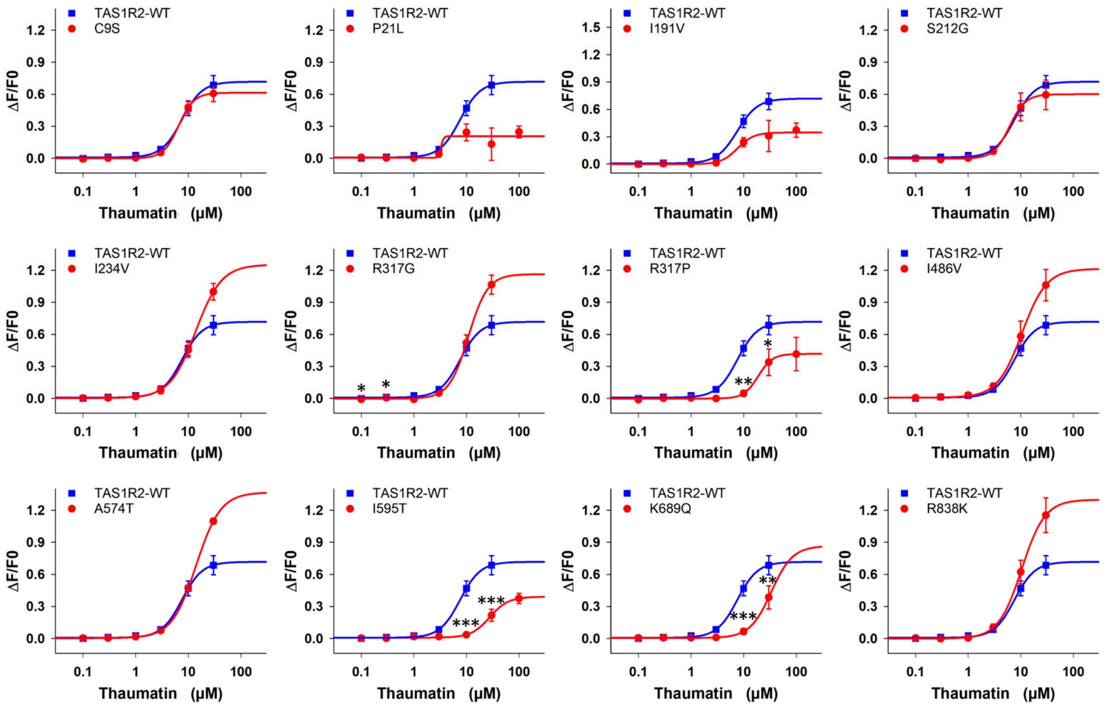
TAS1R2-WT
C9S
P21L
I191V
S212G
I234V
R317G
R317P
I486V
A574T
I595T
K689Q
R838K
ΔF/F0
Thaumatin (μM)

J

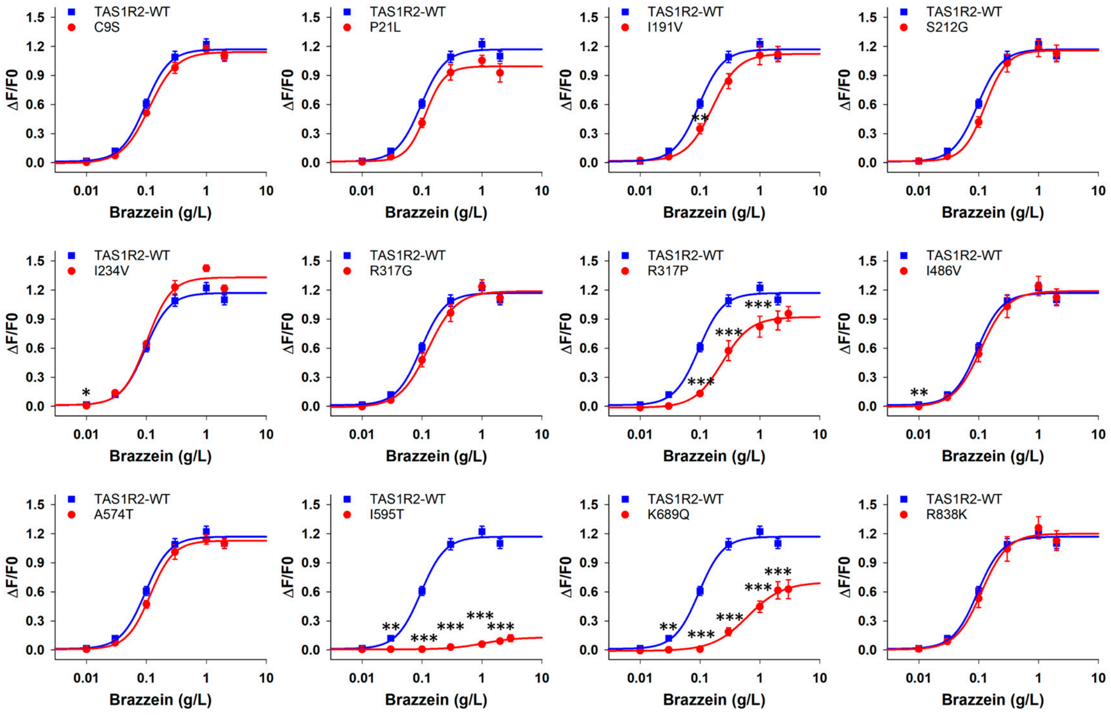
TAS1R2-WT
C9S
P21L
I191V
S212G
I234V
R317G
R317P
I486V
A574T
I595T
K689Q
R838K
ΔF/F0
Brazzein (g/L)

K

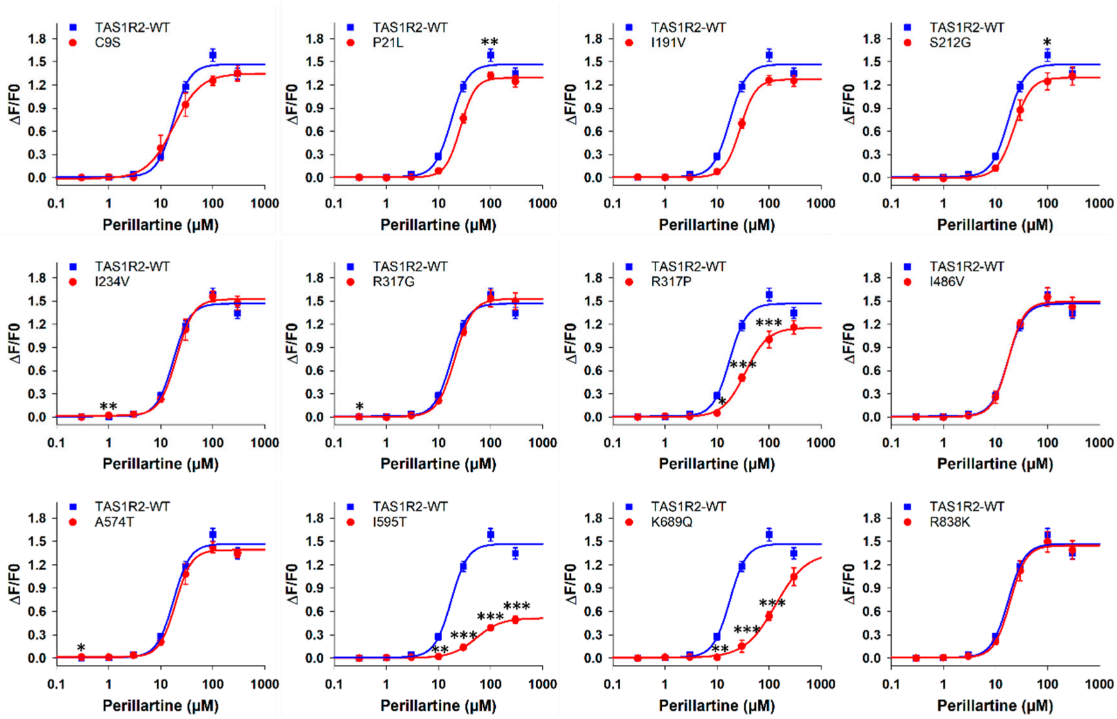

L

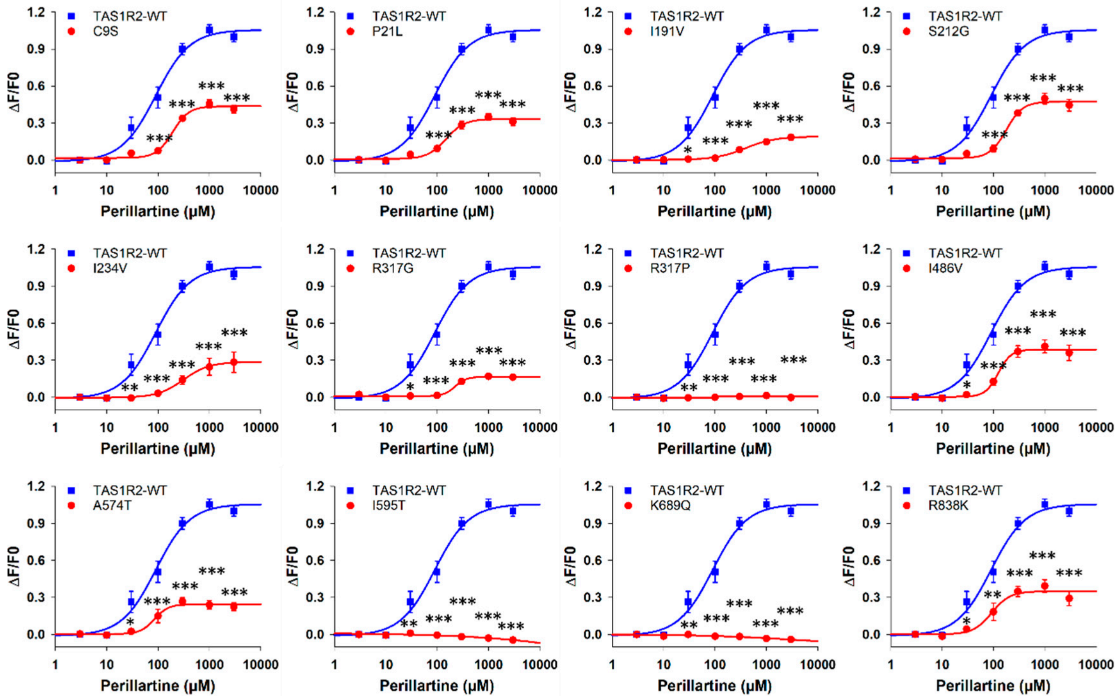

Figure S1. Human TAS1R2-SNP/TAS1R3 dose-response curves with various sweeteners. HEK293T-Gα16gust44 cells were transiently transfected with pcDNA6-MAX-TAS1R2-WT-FLAG (blue line) or pcDNA6-MAX-TAS1R2-SNP-FLAG (red line) and pcDNA4-MAX-TAS1R3-FLAG. A total of 12 TAS1R2 variants were tested for each of the 11 sweeteners (A-L) (excluding of sucralose). For perillartine (L), HEK293T-Gα16gust44 cells were transiently transfected with pcDNA6-MAX-TAS1R2-WT-FLAG (blue dotted line) or pcDNA6-MAX-TAS1R2-SNP-FLAG (red dotted line) only. The data are presented as the mean ± sem of 8 wells from 4 independent experiments. * $p < 0.05$, ** $p < 0.01$, *** $p < 0.001$, calculated using ANOVA followed by Dunnett's test for multiple comparison analysis (with reference to TAS1R2-WT/TAS1R3-WT). The *p*-values are presented in Table S2. WT: wild-type; AceK: acesulfame K; NHDC: neohesperidin dihydrochalcone.

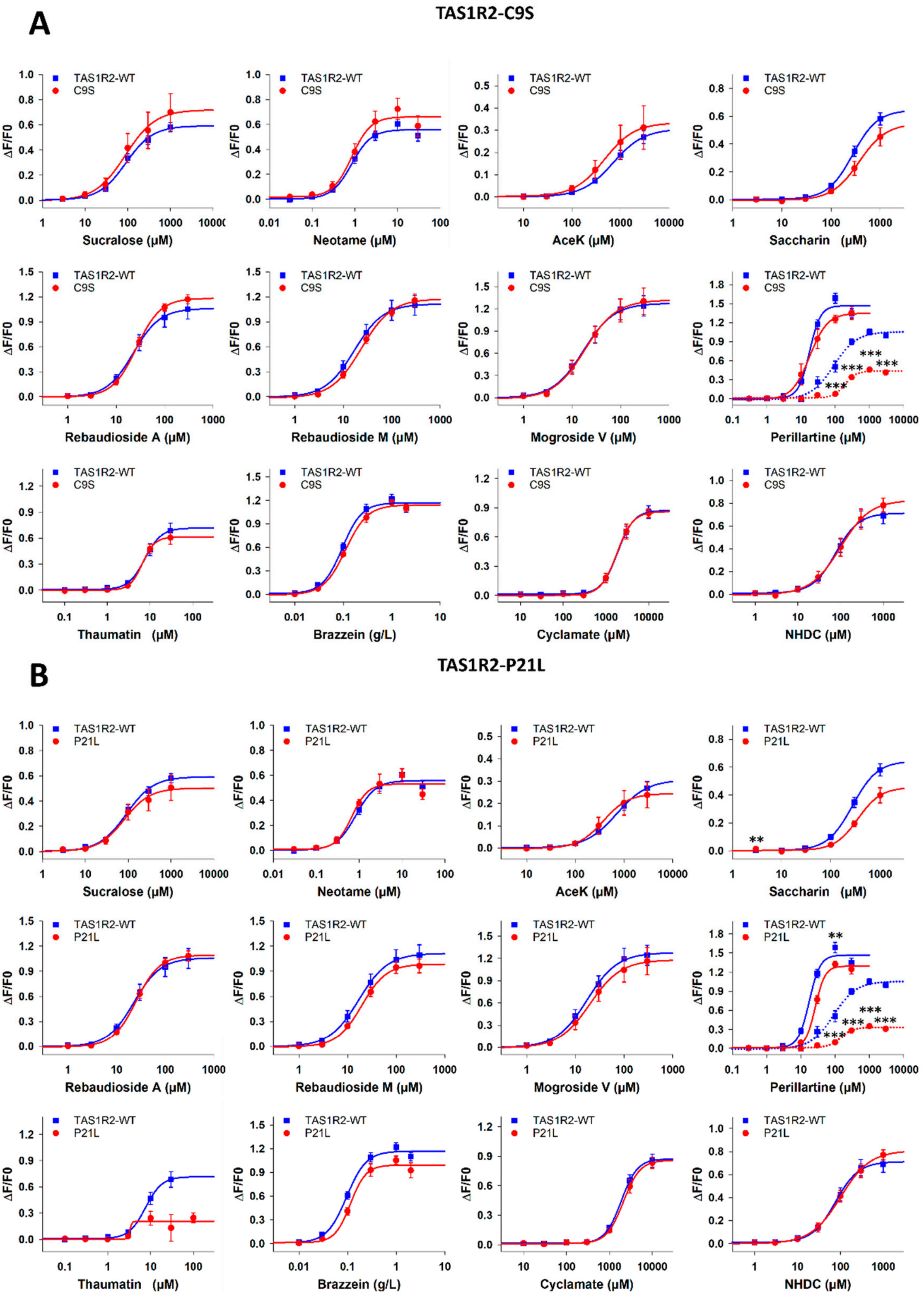
A
TAS1R2-C9S
TAS1R2-WT
C9S
ΔF/F0
Sucralose (μM)
Neotame (μM)
AceK (μM)
Saccharin (μM)
Rebaudioside A (μM)
Rebaudioside M (μM)
Mogroside V (μM)
Perillartine (μM)
Thaumatin (μM)
Brazzein (g/L)
Cyclamate (μM)
NHDC (μM)
***
B
TAS1R2-P21L
TAS1R2-WT
P21L
ΔF/F0
Sucralose (μM)
Neotame (μM)
AceK (μM)
Saccharin (μM)
**
Rebaudioside A (μM)
Rebaudioside M (μM)
Mogroside V (μM)
Perillartine (μM)
***
Thaumatin (μM)
Brazzein (g/L)
Cyclamate (μM)
NHDC (μM)

C TAS1R2-I191V

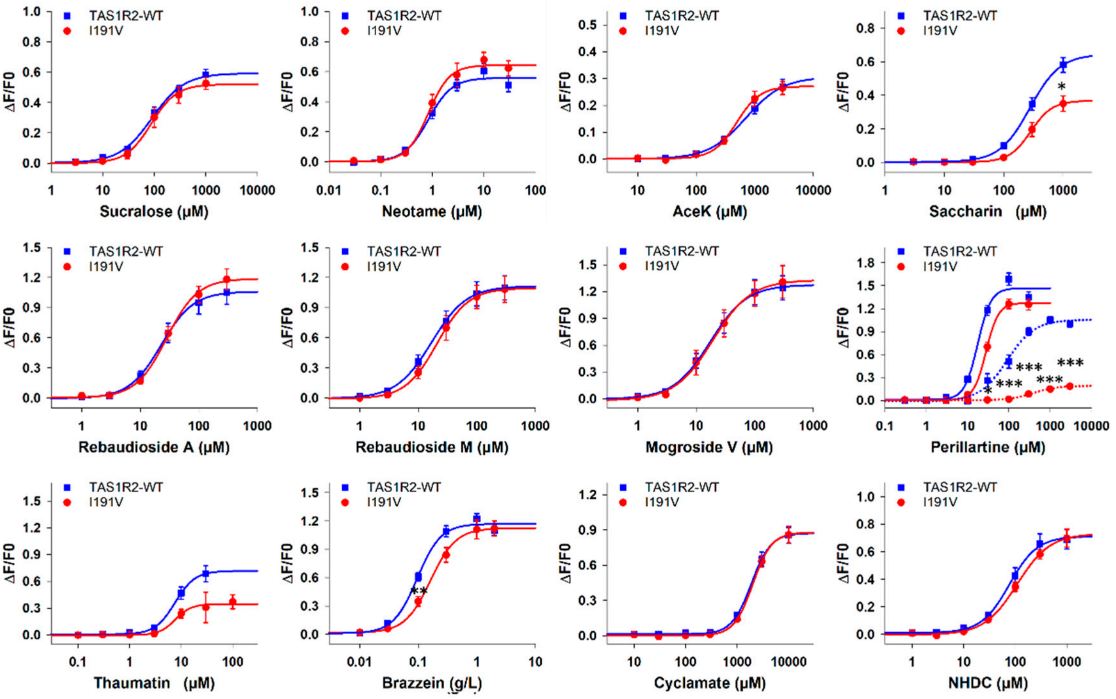

D TAS1R2-S212G

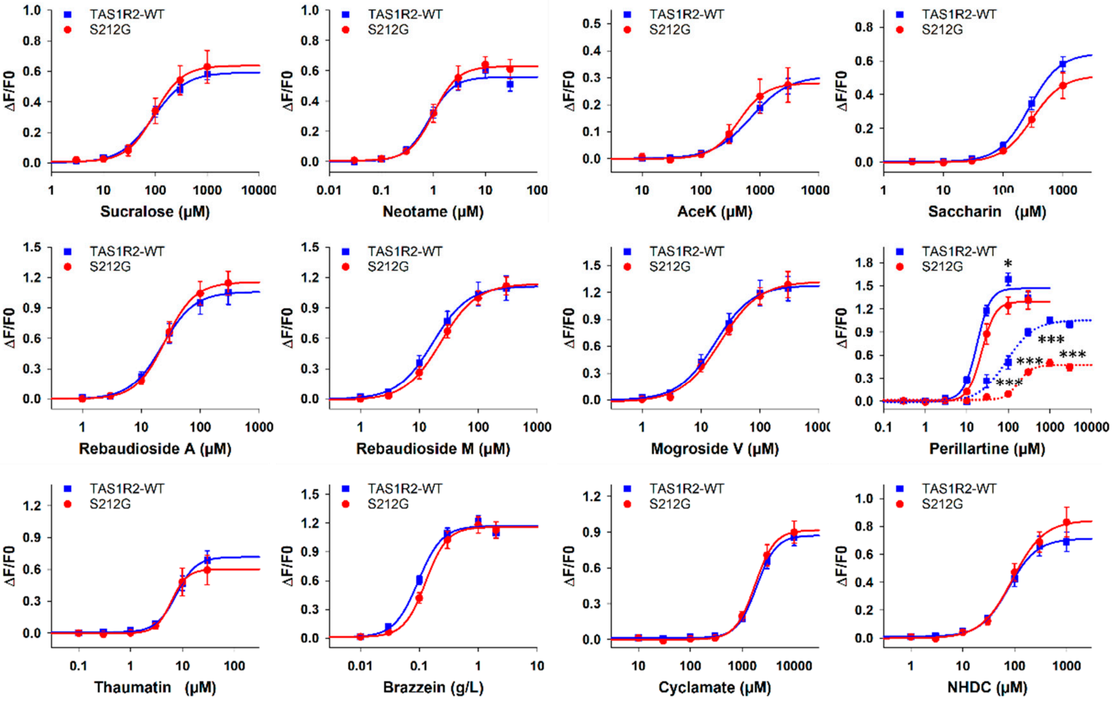

E TAS1R2-I234V

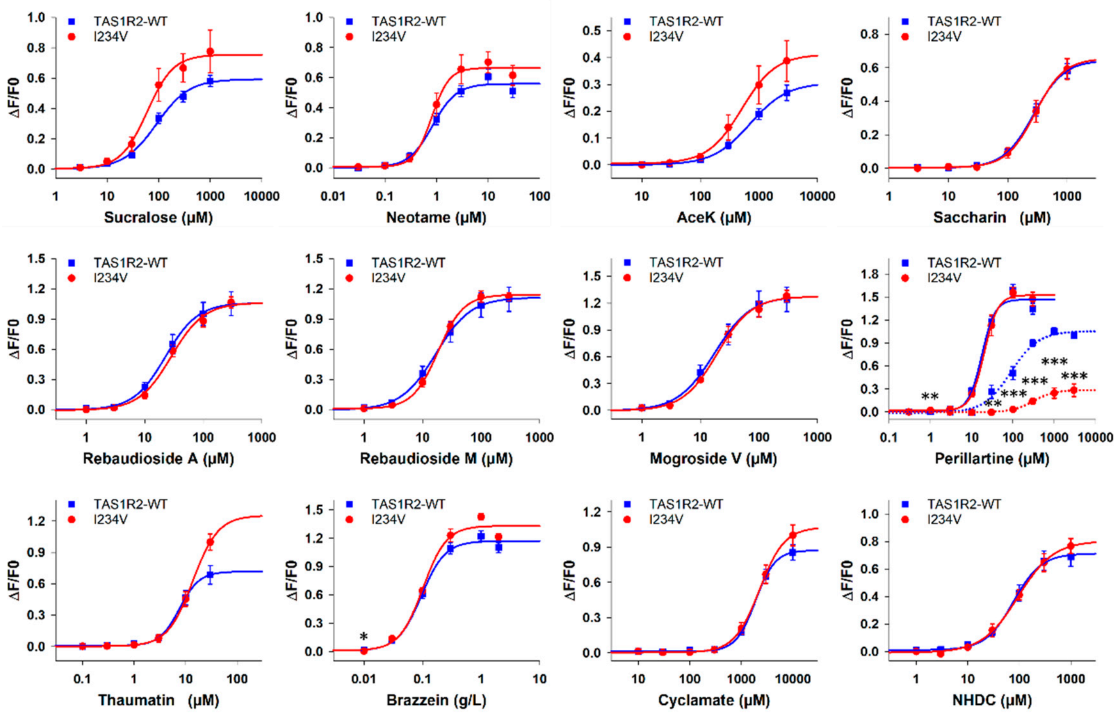

F TAS1R2-R317G

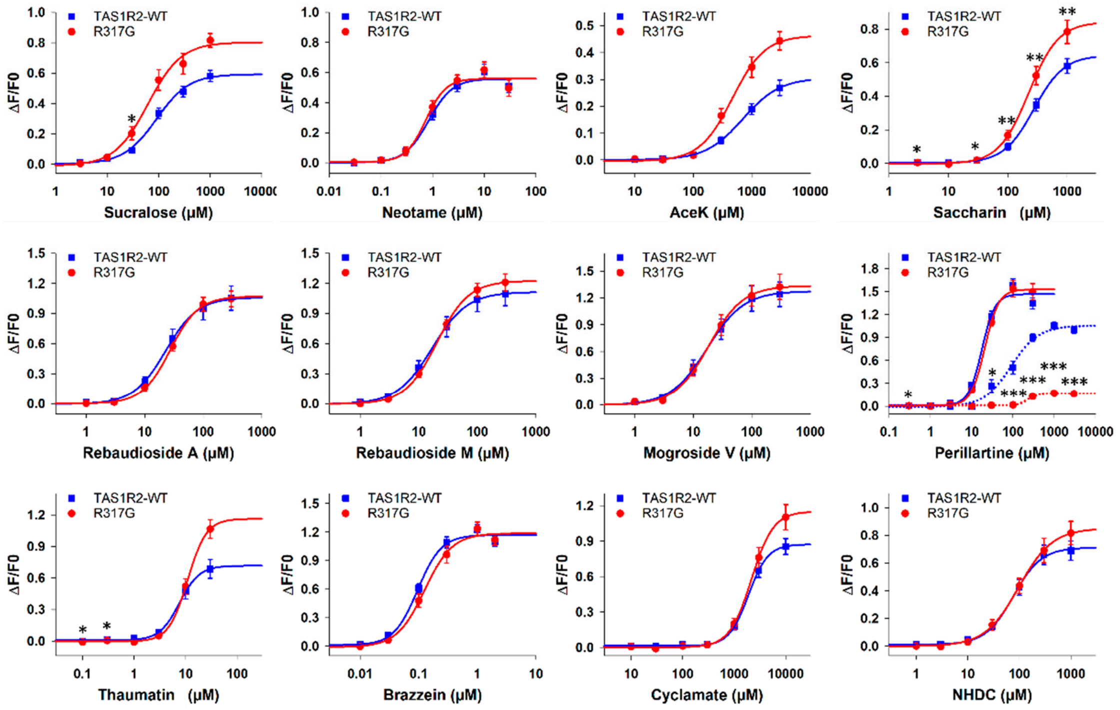

G

TAS1R2-R317P

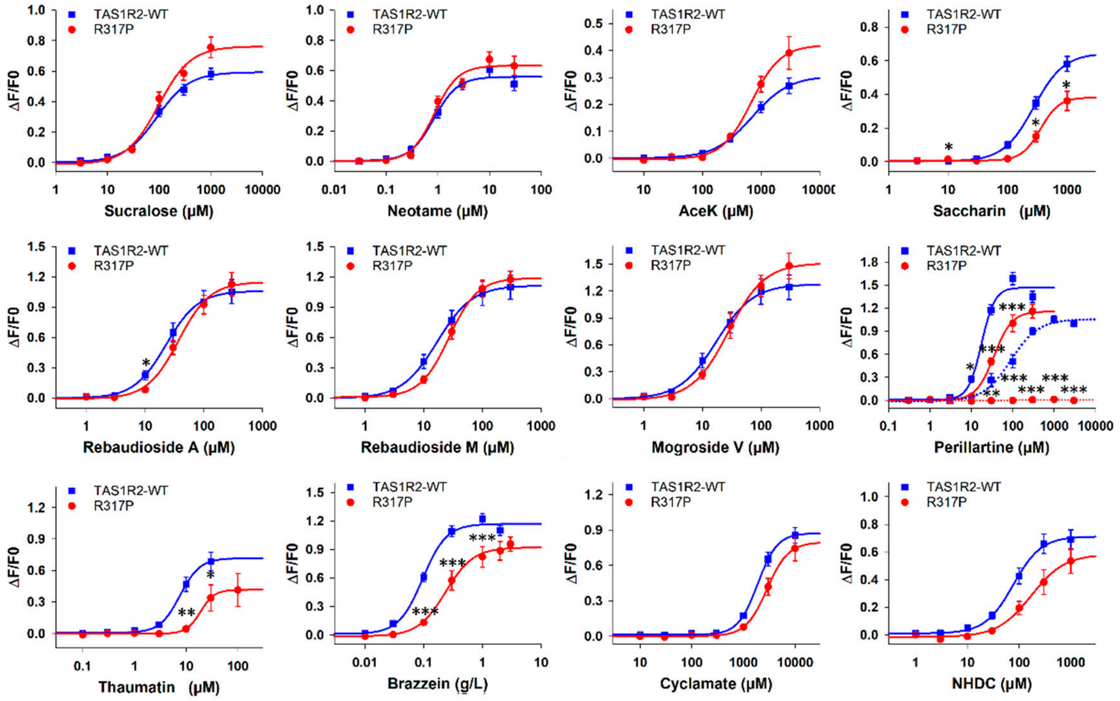

H

TAS1R2-I486V

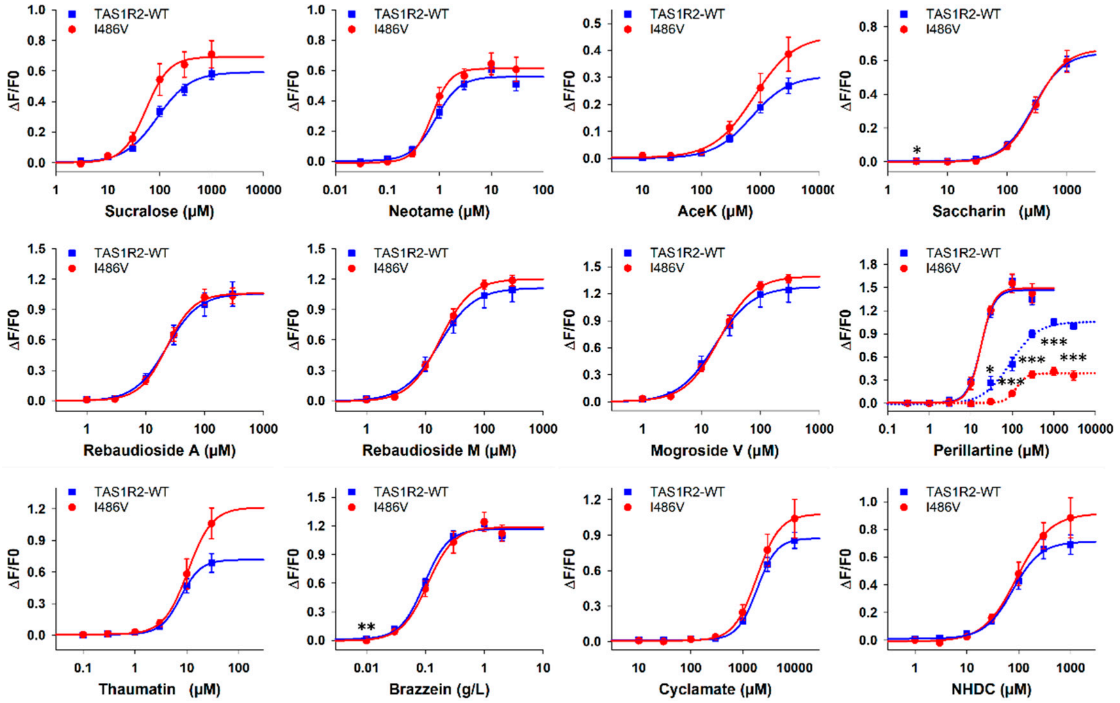

I TAS1R2-A574T

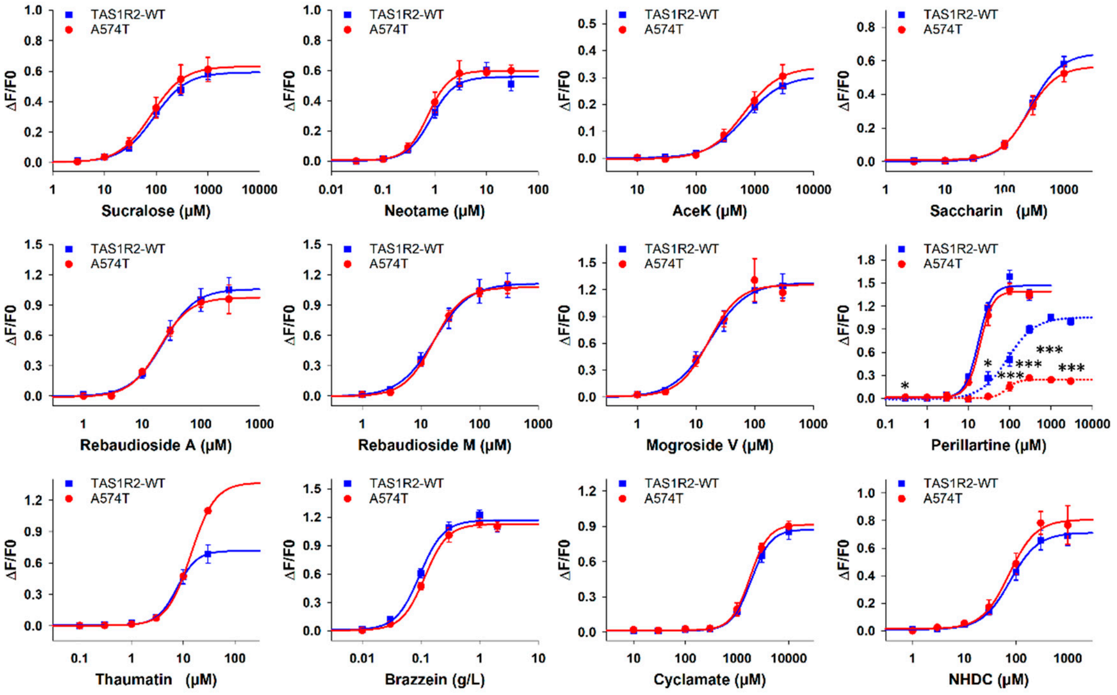

J TAS1R2-I595T

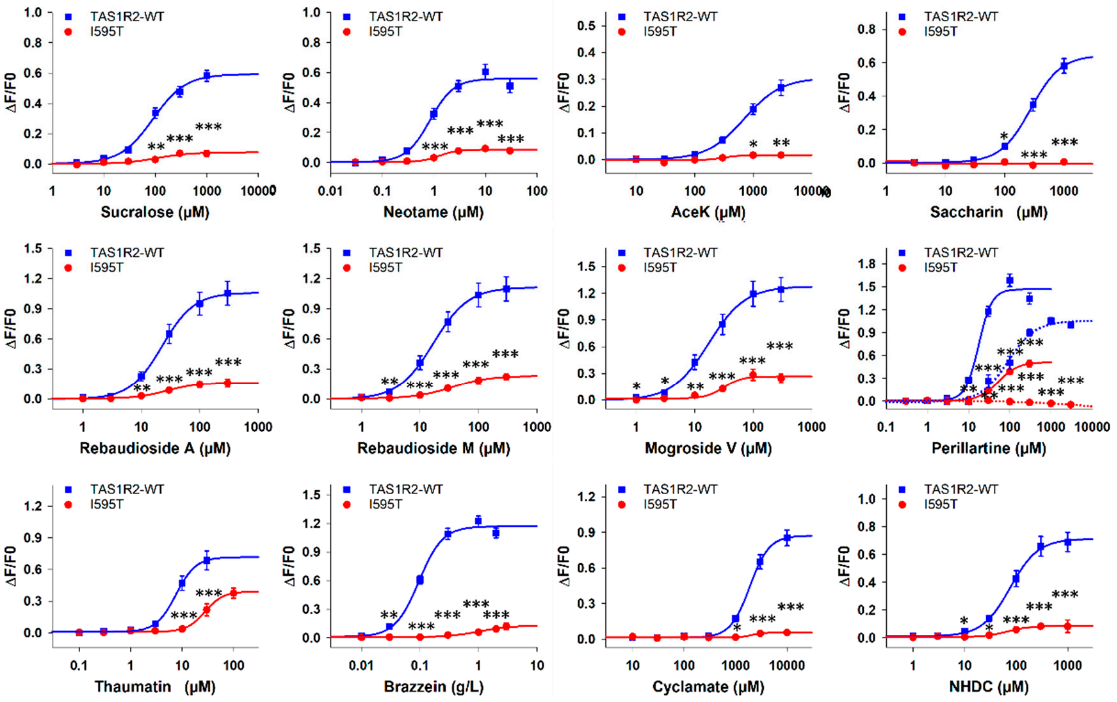

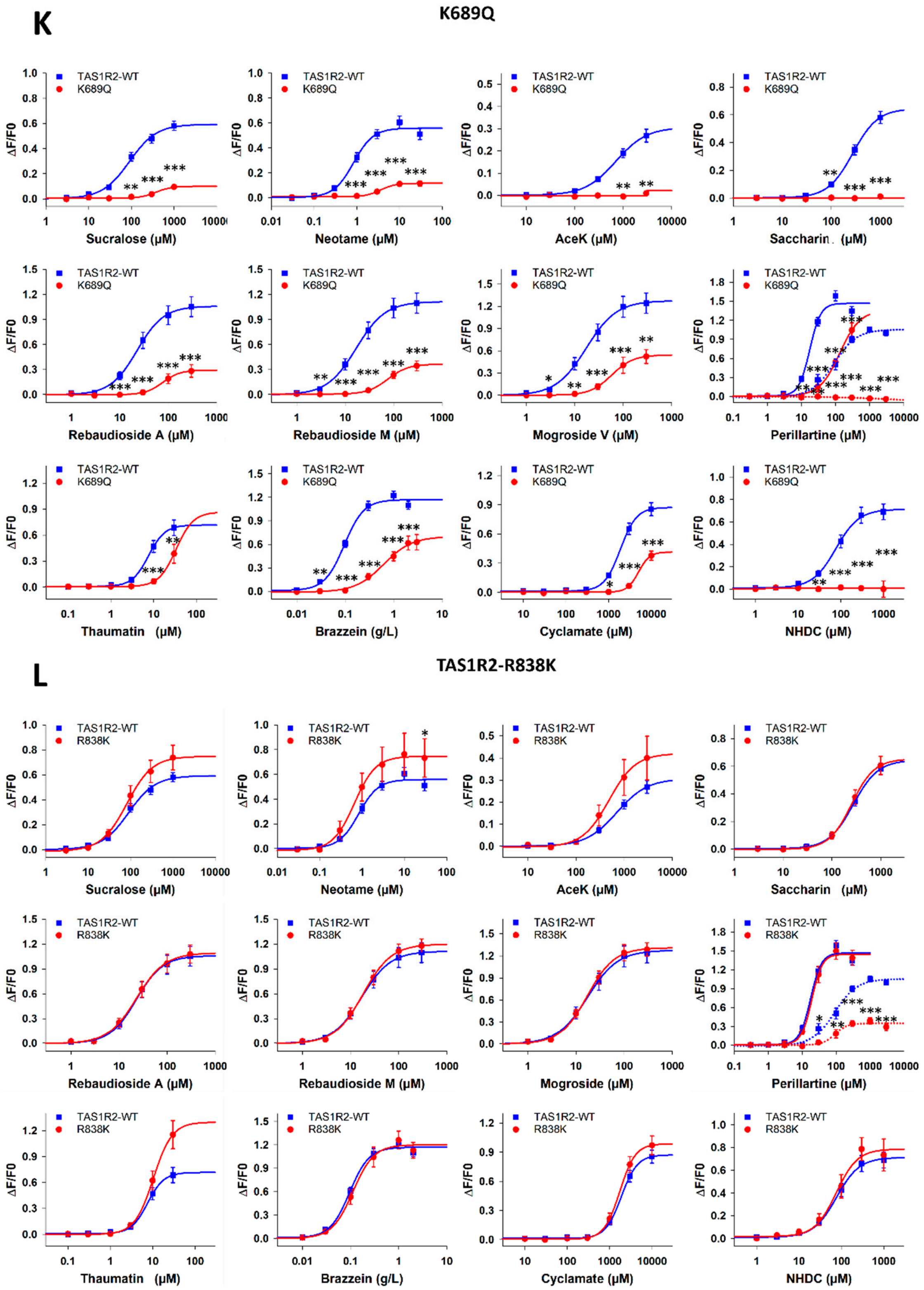

Figure S2. Human TAS1R2-SNP/TAS1R3 dose-response curves with various sweeteners. HEK293T-Gα16gust44 cells were transiently transfected with pcDNA6-MAX-TAS1R2-WT-FLAG (blue line) or pcDNA6-MAX-TAS1R2-SNP-FLAG (red line) and pcDNA4-MAX-TAS1R3-FLAG. A total of 12 TAS1R2 variants (A-L) and 12 sweeteners were tested. For perillartine, HEK293T-Gα16gust44 cells were transiently transfected with pcDNA6-MAX-TAS1R2-WT-FLAG (blue dotted line) or pcDNA6-MAX-TAS1R2-SNP-FLAG (red dotted line) only. The data are presented as the mean ± sem of 8 wells from 4 independent experiments. * $p < 0.05$, ** $p < 0.01$, *** $p < 0.001$, calculated using ANOVA followed by Dunnett's test for multiple comparison analysis (with reference to TAS1R2-WT/TAS1R3-WT). The *p*-values are presented in Table S2. WT: wild-type; AceK: acesulfame K; NHDC: neohesperidin dihydrochalcone.

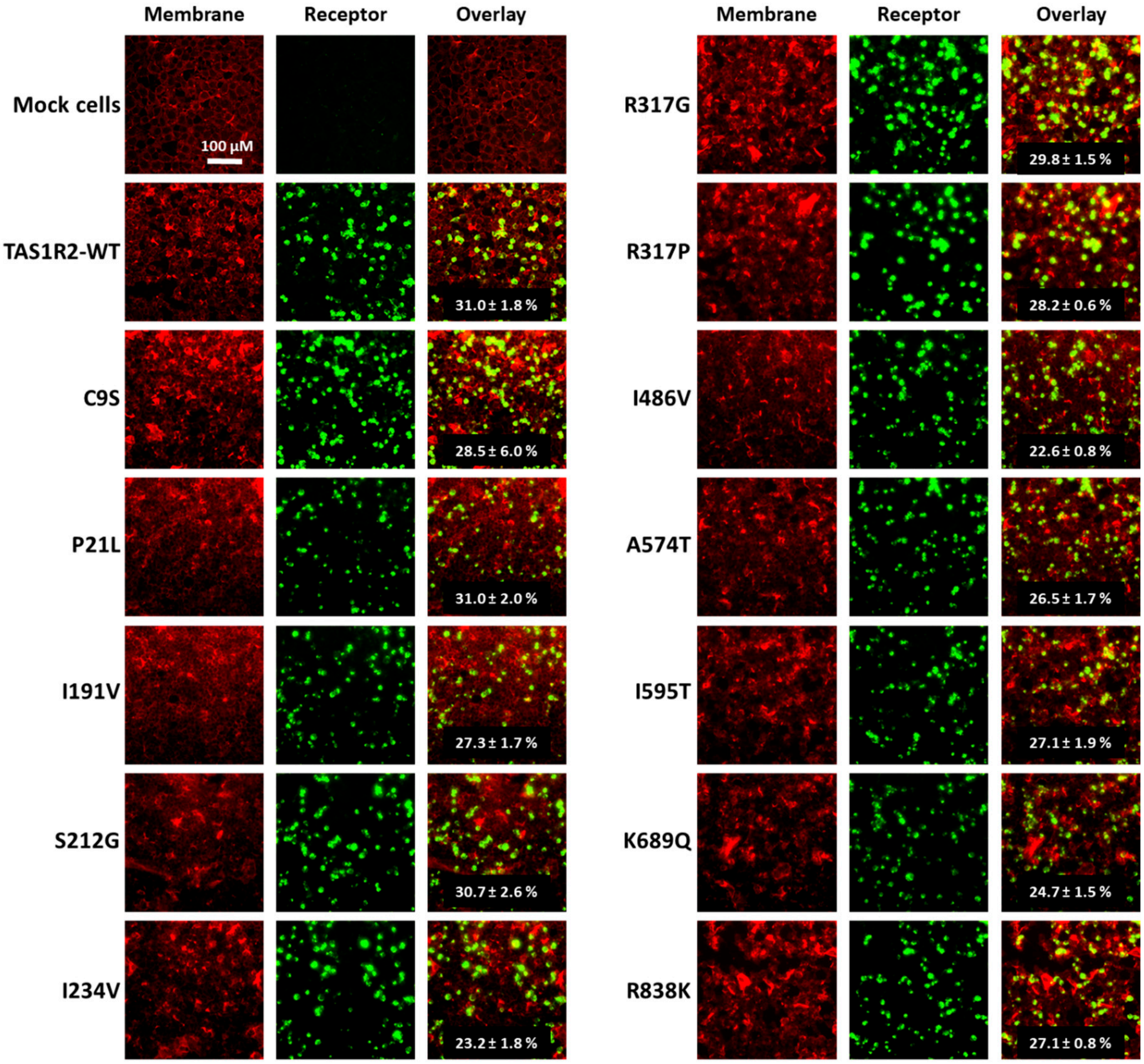

Figure S3. Immunocytochemistry of HEK293T-Gα16gust44 cells expressing FLAG-tagged TAS1R2-SNPs constructs. The TAS1R-expressing cells are shown in green. and the plasma membrane is stained in red. The receptors were detected using a primary anti-FLAG antibody and fluorescently labelled by a secondary Alexa-488–conjugated antibody. All data were obtained from the same transfection experiment. HEK293T-Gα16gust44 cells in the absence of the TAS1R receptor (mock cells) showed no signal. Pictures were taken using an epi-fluorescence inverted microscope (Eclipse TiE. Nikon. Champigny sur Marne. France) equipped with an x20 objective lens and a LucaR EMCCD camera (Andor Technology. Belfast. UK). The average cell fraction expressing the receptor (± sem) is provided in white in the overlay panel. Four to six images were counted. and averaged per receptor construct pictures. WT: wild-type.

A

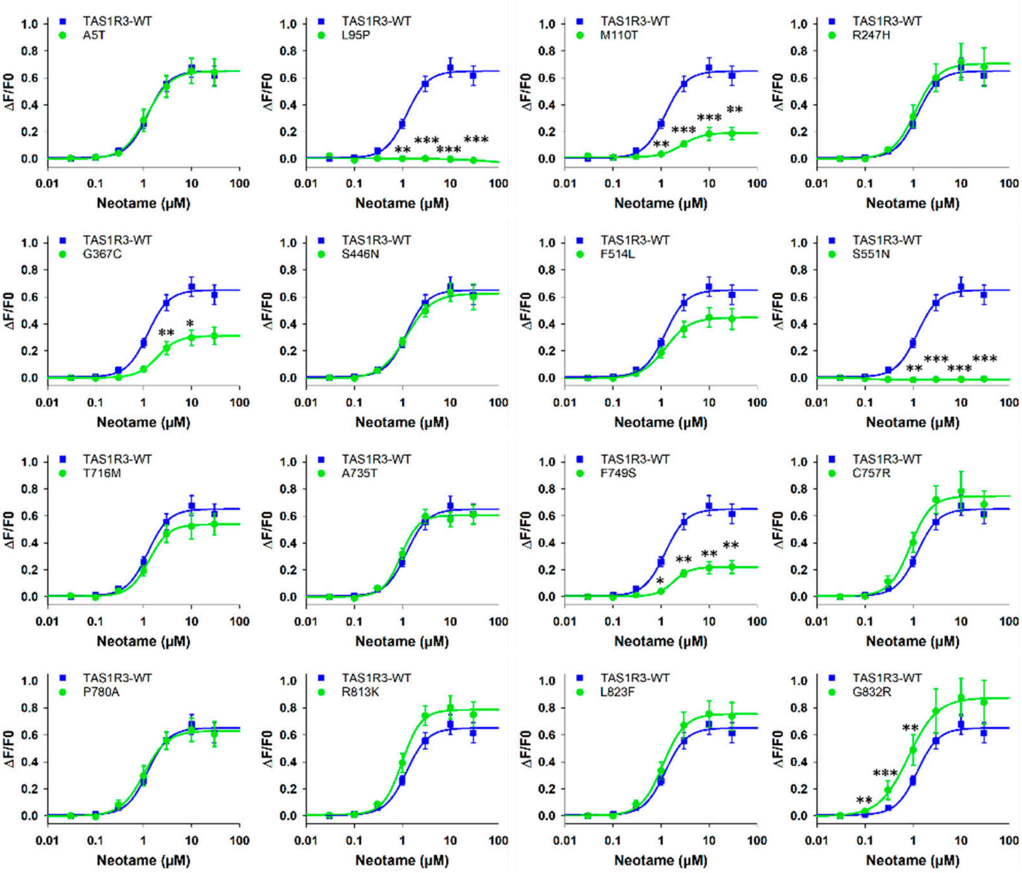

TAS1R3-WT
A5T
L95P
M110T
R247H
G367C
S446N
F514L
S551N
T716M
A735T
F749S
C757R
P780A
R813K
L823F
G832R
ΔF/F0
Neotame (μM)

B

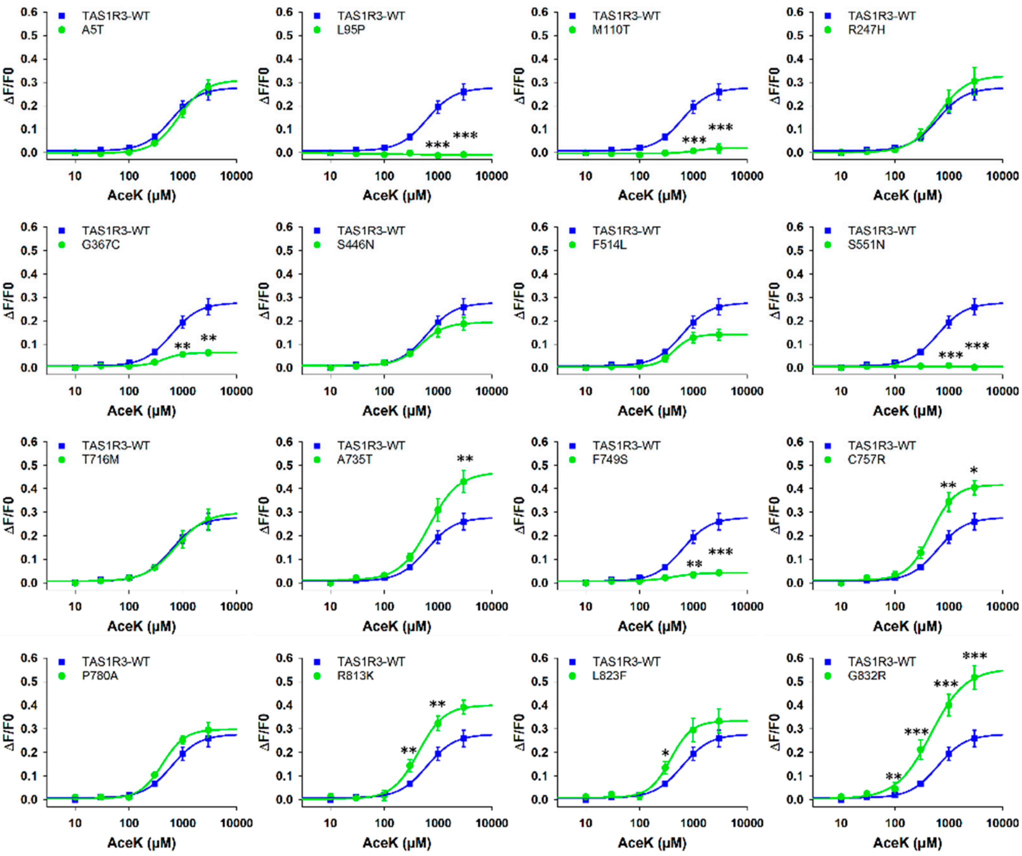

TAS1R3-WT
A5T
L95P
M110T
R247H
G367C
S446N
F514L
S551N
T716M
A735T
F749S
C757R
P780A
R813K
L823F
G832R
ΔF/F0
AceK (μM)

C

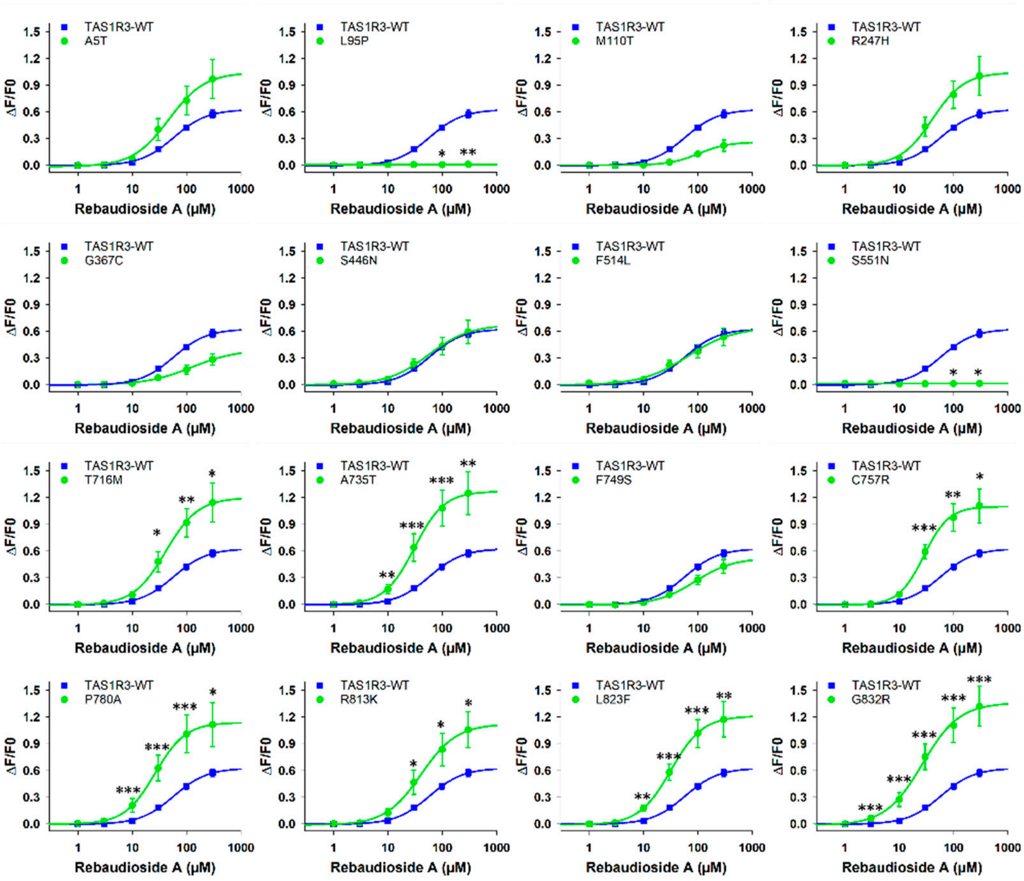
TAS1R3-WT
A5T
L95P
M110T
R247H
G367C
S446N
F514L
S551N
T716M
A735T
F749S
C757R
P780A
R813K
L823F
G832R
ΔF/F0
Rebaudioside A (μM)

D

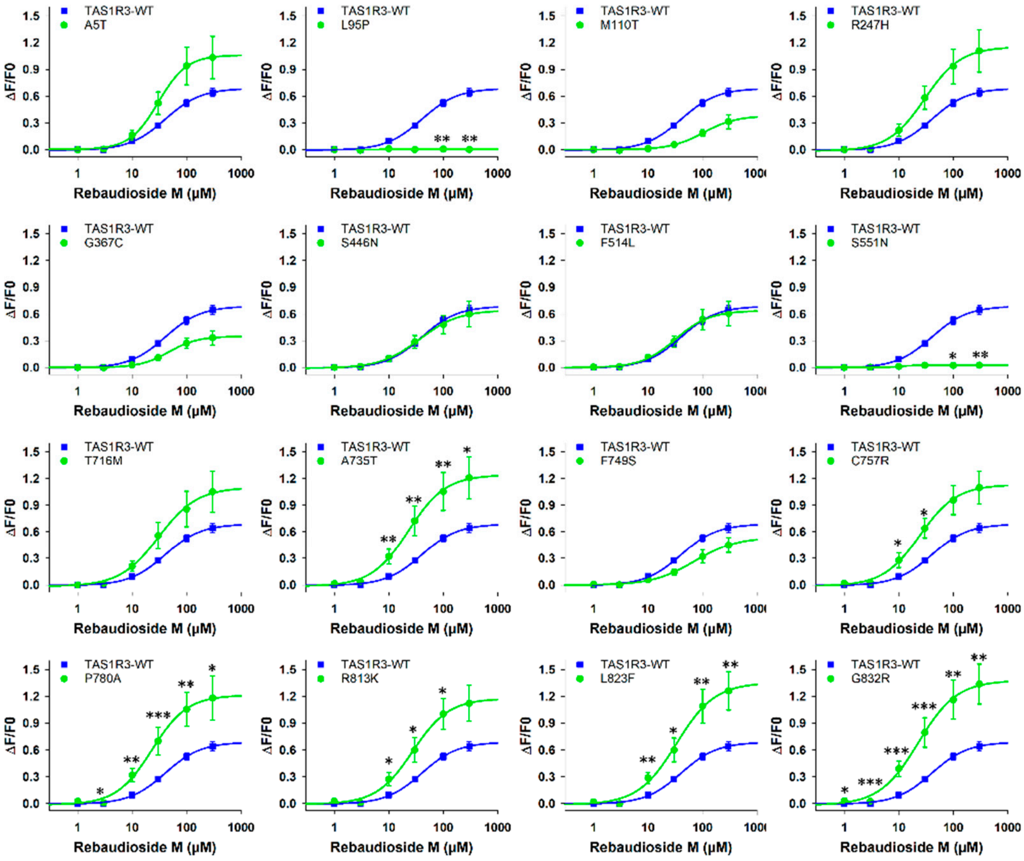
TAS1R3-WT
A5T
L95P
M110T
R247H
G367C
S446N
F514L
S551N
T716M
A735T
F749S
C757R
P780A
R813K
L823F
G832R
ΔF/F0
Rebaudioside M (μM)

E

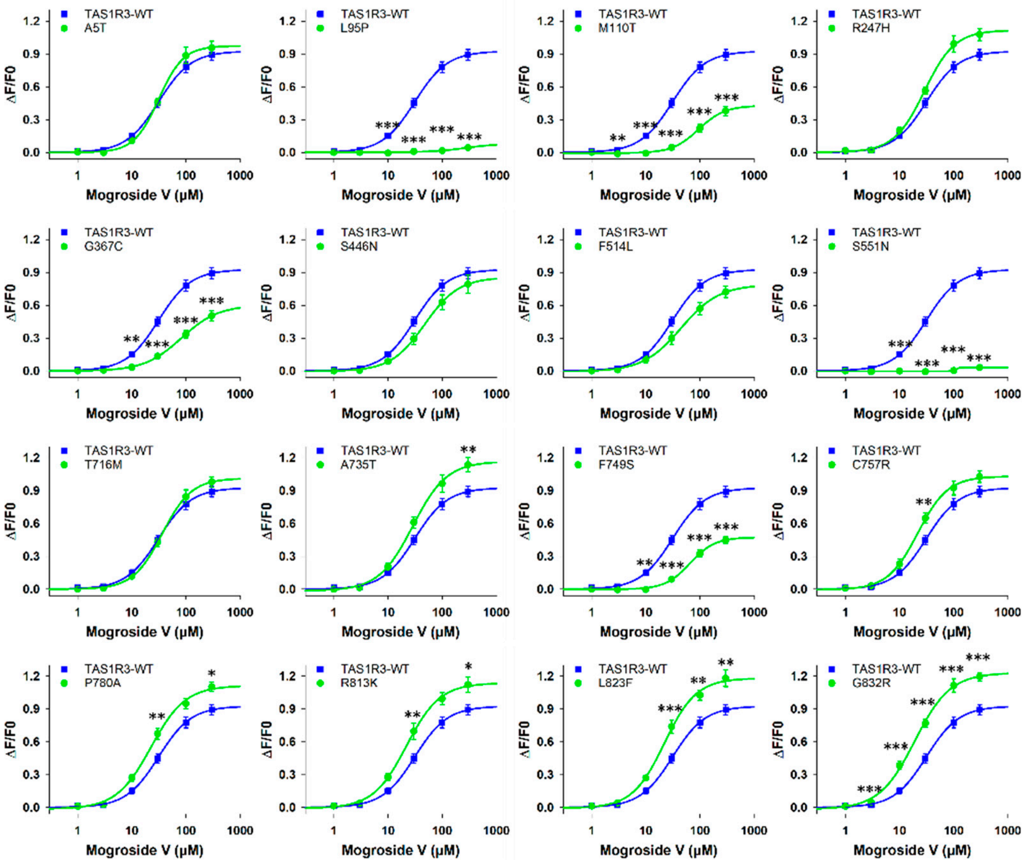
TAS1R3-WT
A5T
ΔF/F0
Mogroside V (μM)
TAS1R3-WT
L95P
ΔF/F0
Mogroside V (μM)
TAS1R3-WT
M110T
ΔF/F0
Mogroside V (μM)
TAS1R3-WT
R247H
ΔF/F0
Mogroside V (μM)
TAS1R3-WT
G367C
ΔF/F0
Mogroside V (μM)
TAS1R3-WT
S446N
ΔF/F0
Mogroside V (μM)
TAS1R3-WT
F514L
ΔF/F0
Mogroside V (μM)
TAS1R3-WT
S551N
ΔF/F0
Mogroside V (μM)
TAS1R3-WT
T716M
ΔF/F0
Mogroside V (μM)
TAS1R3-WT
A735T
ΔF/F0
Mogroside V (μM)
TAS1R3-WT
F749S
ΔF/F0
Mogroside V (μM)
TAS1R3-WT
C757R
ΔF/F0
Mogroside V (μM)
TAS1R3-WT
P780A
ΔF/F0
Mogroside V (μM)
TAS1R3-WT
R813K
ΔF/F0
Mogroside V (μM)
TAS1R3-WT
L823F
ΔF/F0
Mogroside V (μM)
TAS1R3-WT
G832R
ΔF/F0
Mogroside V (μM)

F

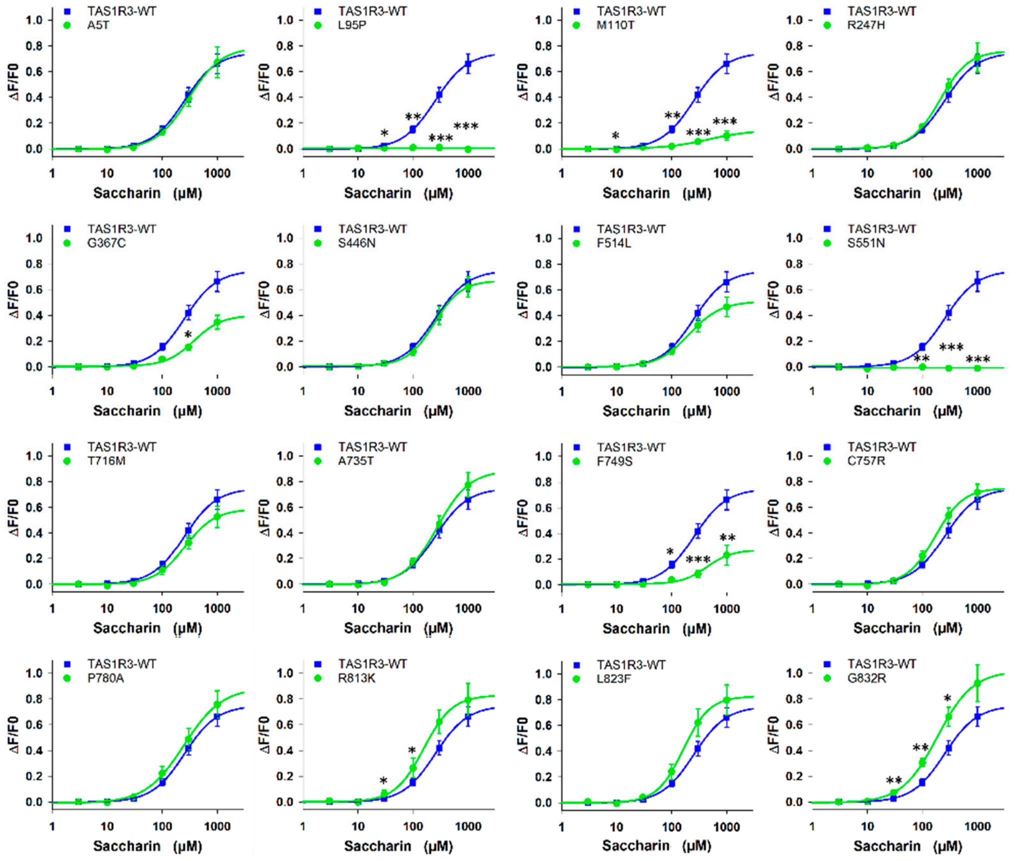
TAS1R3-WT
A5T
ΔF/F0
Saccharin (μM)
TAS1R3-WT
L95P
ΔF/F0
Saccharin (μM)
TAS1R3-WT
M110T
ΔF/F0
Saccharin (μM)
TAS1R3-WT
R247H
ΔF/F0
Saccharin (μM)
TAS1R3-WT
G367C
ΔF/F0
Saccharin (μM)
TAS1R3-WT
S446N
ΔF/F0
Saccharin (μM)
TAS1R3-WT
F514L
ΔF/F0
Saccharin (μM)
TAS1R3-WT
S551N
ΔF/F0
Saccharin (μM)
TAS1R3-WT
T716M
ΔF/F0
Saccharin (μM)
TAS1R3-WT
A735T
ΔF/F0
Saccharin (μM)
TAS1R3-WT
F749S
ΔF/F0
Saccharin (μM)
TAS1R3-WT
C757R
ΔF/F0
Saccharin (μM)
TAS1R3-WT
P780A
ΔF/F0
Saccharin (μM)
TAS1R3-WT
R813K
ΔF/F0
Saccharin (μM)
TAS1R3-WT
L823F
ΔF/F0
Saccharin (μM)
TAS1R3-WT
G832R
ΔF/F0
Saccharin (μM)

G

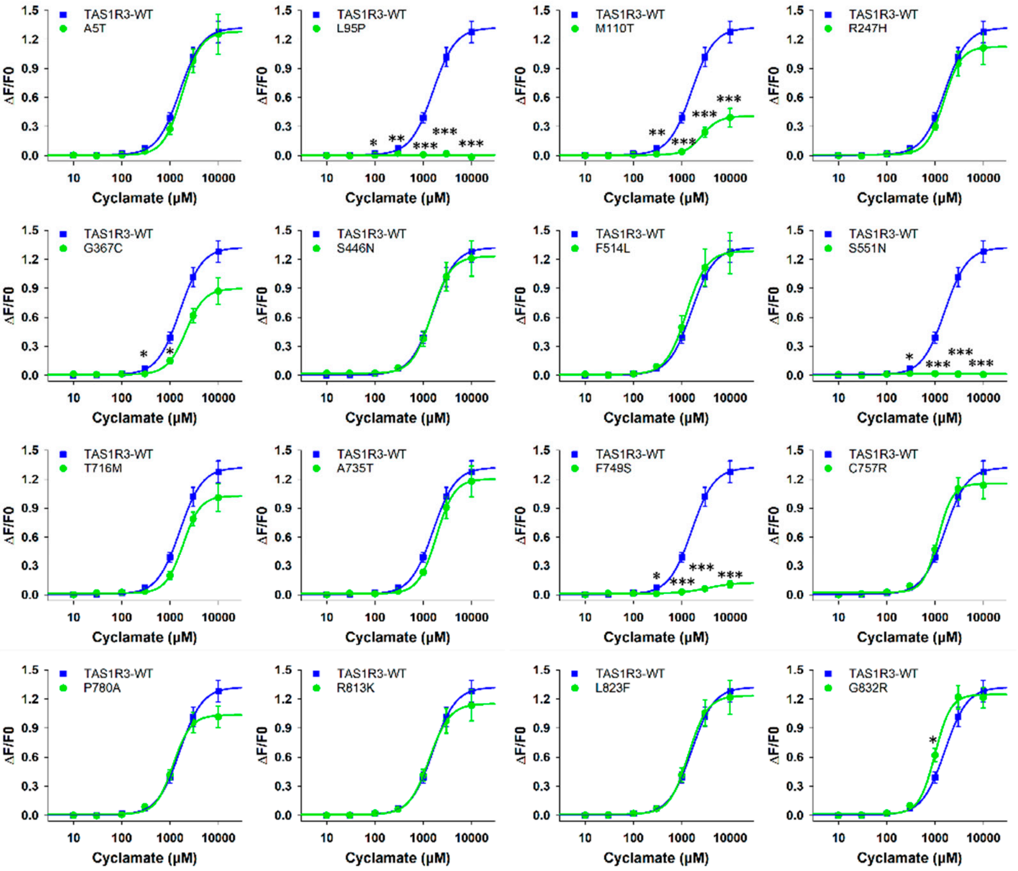
TAS1R3-WT
A5T
L95P
M110T
R247H
G367C
S446N
F514L
S551N
T716M
A735T
F749S
C757R
P780A
R813K
L823F
G832R
ΔF/F0
0.0
0.3
0.6
0.9
1.2
1.5
10
100
1000
10000
Cyclamate (μM)

H

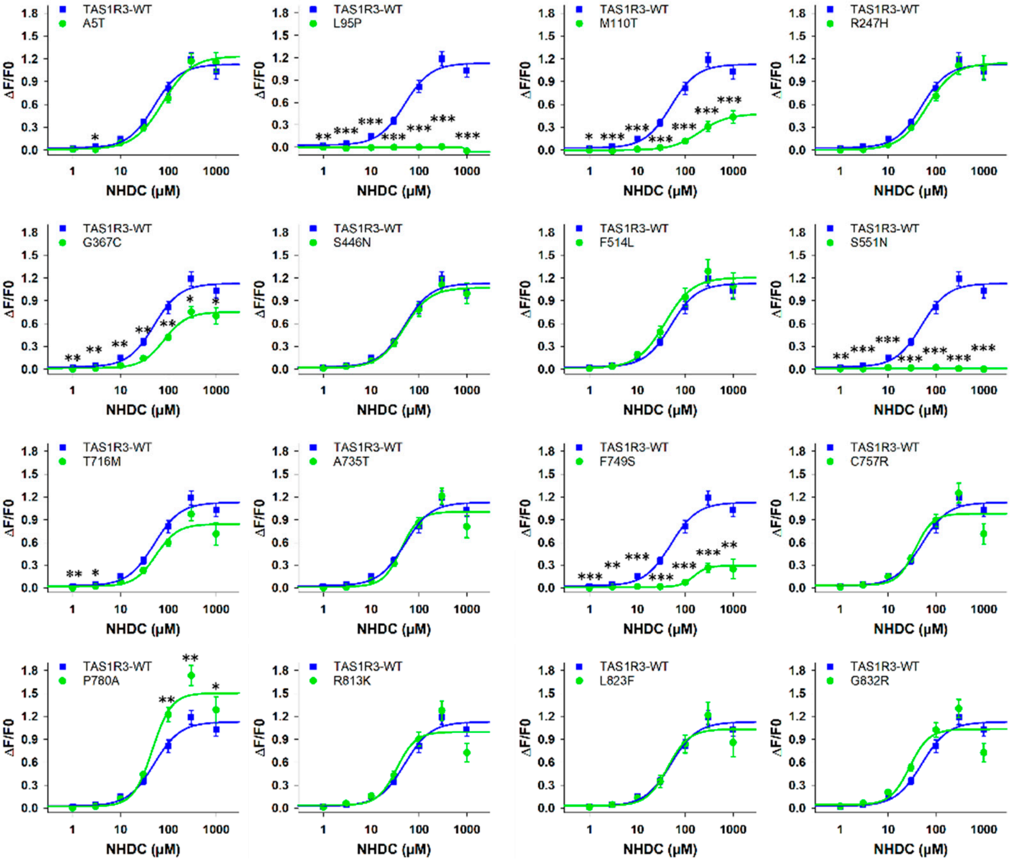
TAS1R3-WT
A5T
L95P
M110T
R247H
G367C
S446N
F514L
S551N
T716M
A735T
F749S
C757R
P780A
R813K
L823F
G832R
ΔF/F0
0.0
0.3
0.6
0.9
1.2
1.5
1.8
1
10
100
1000
NHDC (μM)

I

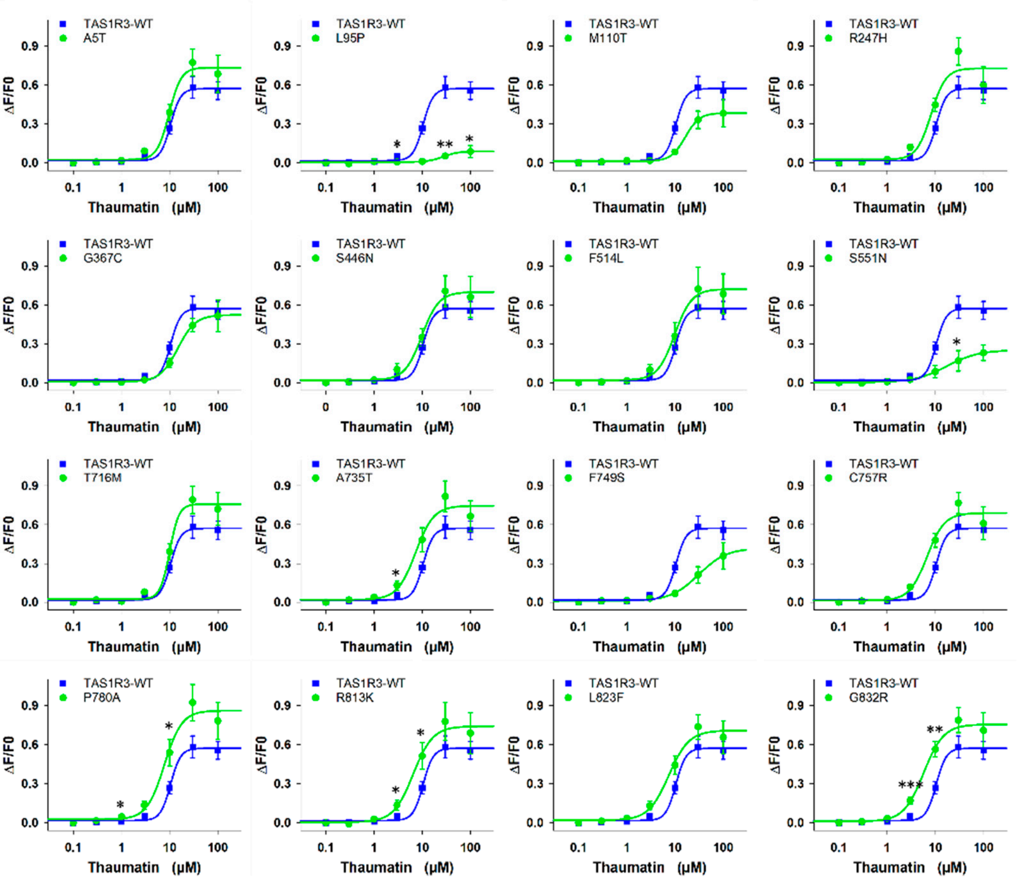

TAS1R3-WT
A5T
L95P
M110T
R247H
G367C
S446N
F514L
S551N
T716M
A735T
F749S
C757R
P780A
R813K
L823F
G832R
ΔF/F0
Thaumatin (μM)

J

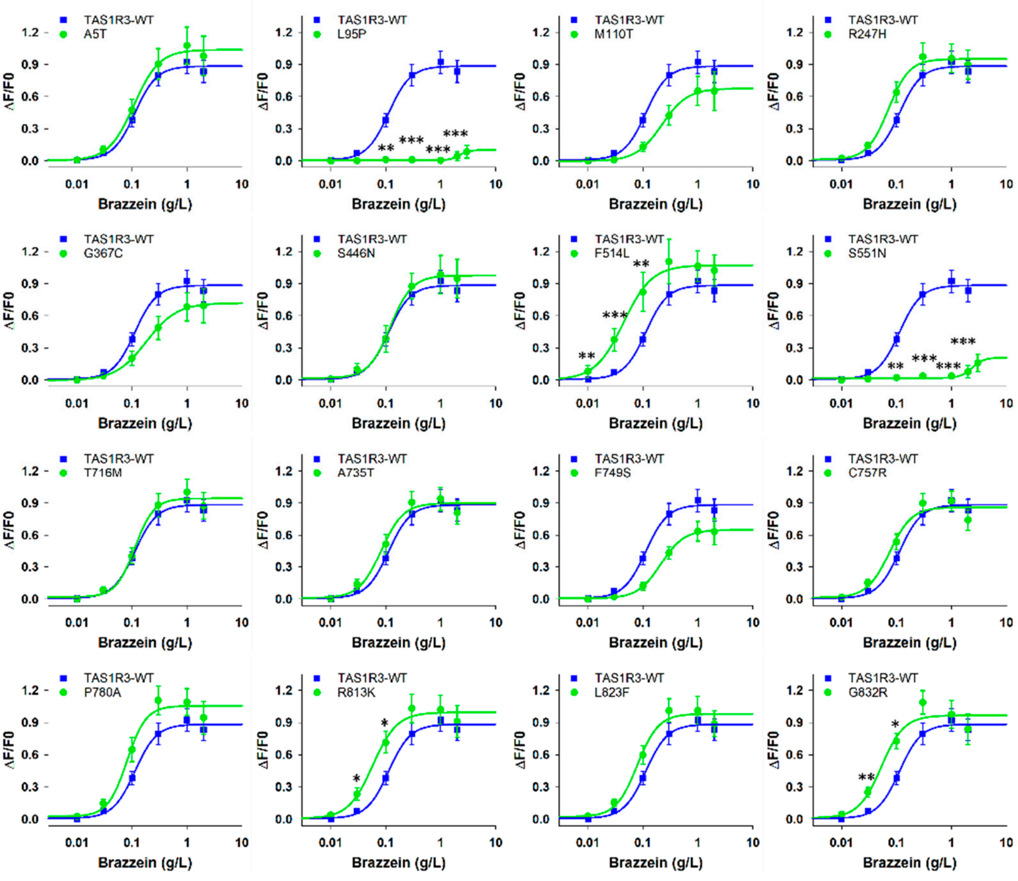

TAS1R3-WT
A5T
L95P
M110T
R247H
G367C
S446N
F514L
S551N
T716M
A735T
F749S
C757R
P780A
R813K
L823F
G832R
ΔF/F0
Brazzein (g/L)

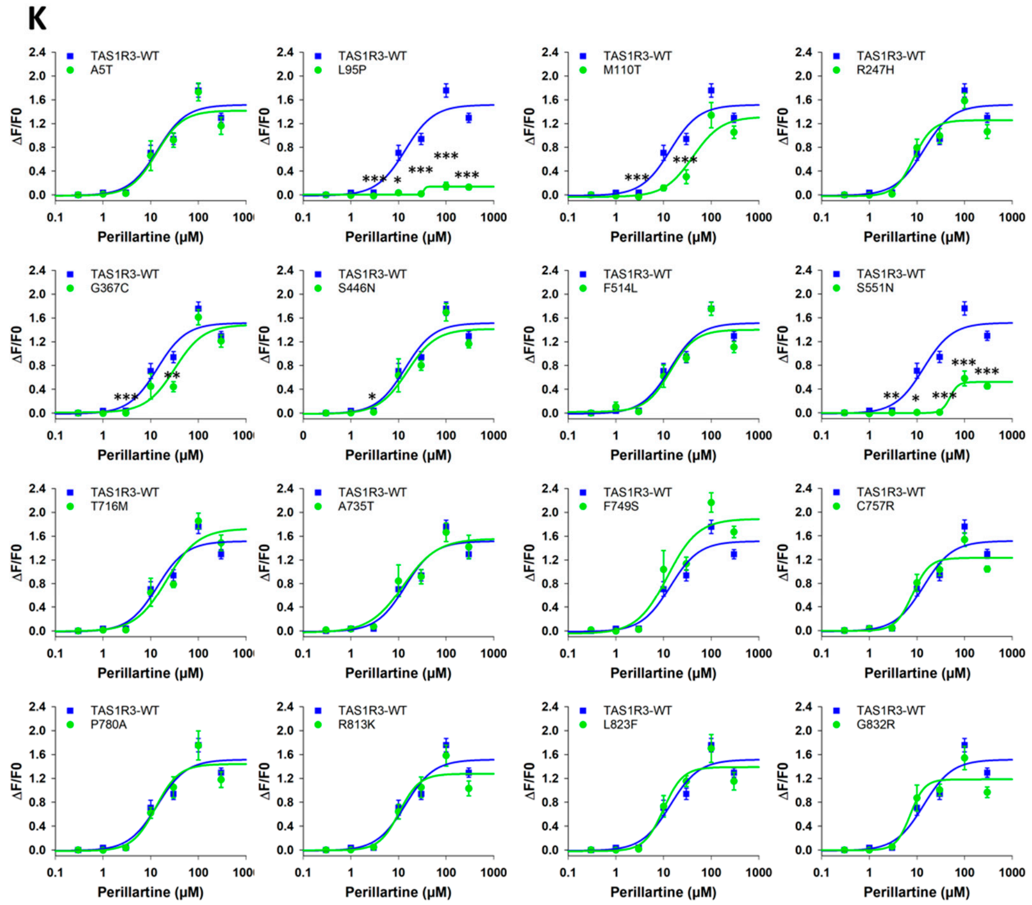

Figure S4. Human TAS1R2/TAS1R3-SNP dose-response curves with various sweeteners. HEK293T-Gα16gust44 cells were transiently transfected with pcDNA6-MAX-TAS1R2-FLAG and pcDNA4-MAX-TAS1R3-WT-FLAG (blue line) or pcDNA4-MAX-TAS1R3-SNP-FLAG (green line). A total of 16 TAS1R3 variants were tested for each of the 11 sweeteners (A-K) (excluding of sucralose). * $p < 0.05$. ** $p < 0.01$. *** $p < 0.001$. calculated using ANOVA followed by Dunnett's test for multiple comparison analysis (with reference to TAS1R2-WT/TAS1R3-WT). The data are presented as the mean ± sem of 8 wells from 4 independent experiments. The *p*-values are presented in Table S3. WT: wild-type; AceK: acesulfame K; NHDC: neohesperidin dihydrochalcone.

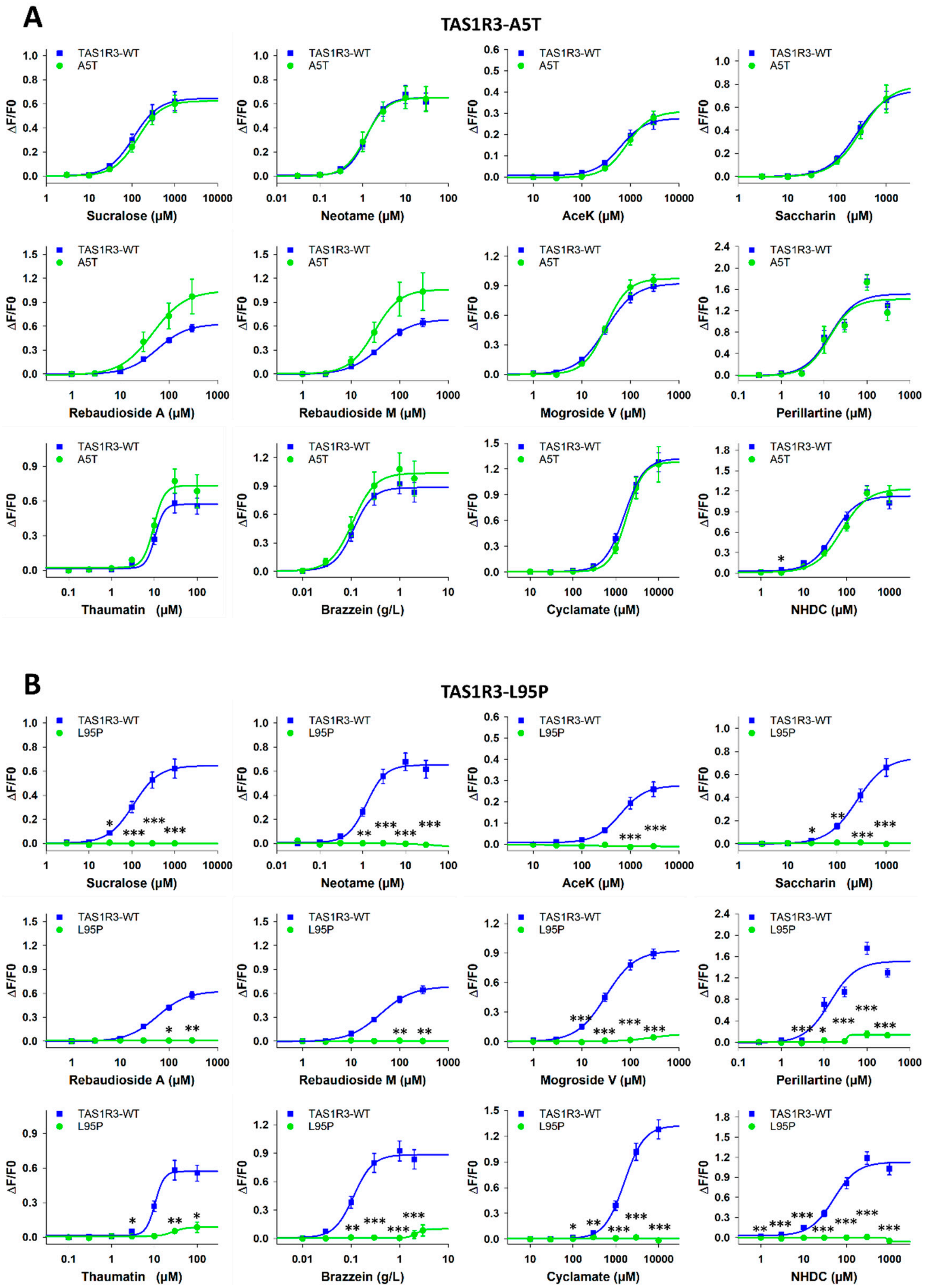
A
TAS1R3-A5T
TAS1R3-WT
A5T
ΔF/F0
Sucralose (μM)
Neotame (μM)
AceK (μM)
Saccharin (μM)
Rebaudioside A (μM)
Rebaudioside M (μM)
Mogroside V (μM)
Perillartine (μM)
Thaumatin (μM)
Brazzein (g/L)
Cyclamate (μM)
NHDC (μM)
B
TAS1R3-L95P
TAS1R3-WT
L95P
ΔF/F0
Sucralose (μM)
Neotame (μM)
AceK (μM)
Saccharin (μM)
Rebaudioside A (μM)
Rebaudioside M (μM)
Mogroside V (μM)
Perillartine (μM)
Thaumatin (μM)
Brazzein (g/L)
Cyclamate (μM)
NHDC (μM)

C

TAS1R3-M110T

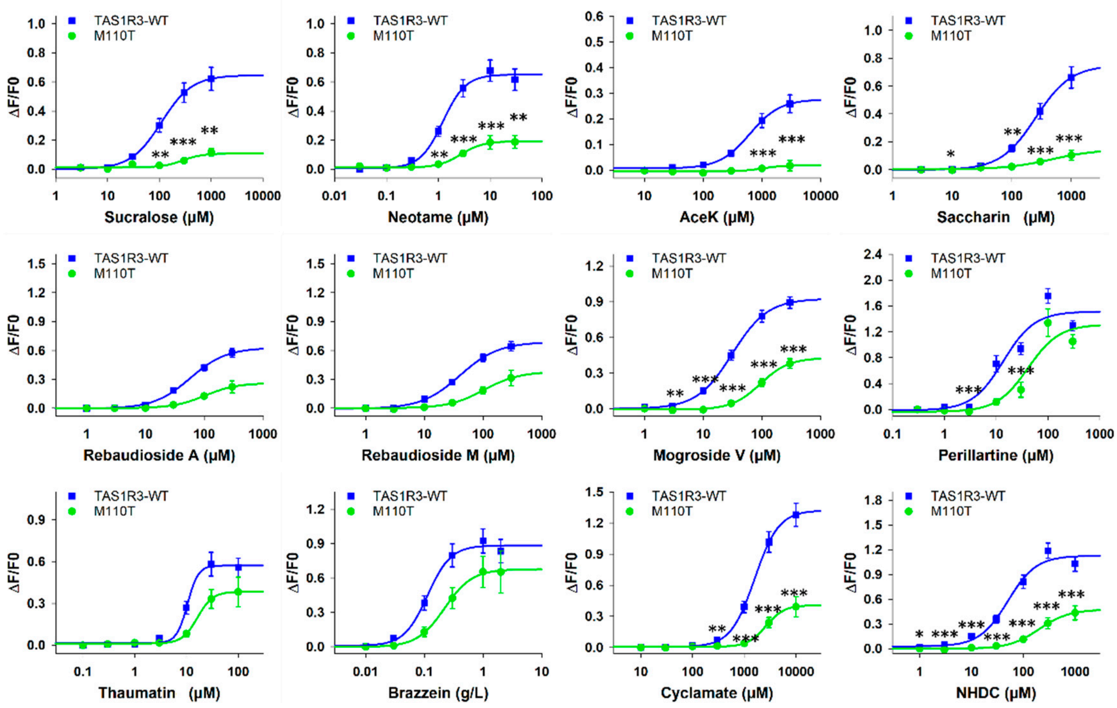

D

TAS1R3-R247H

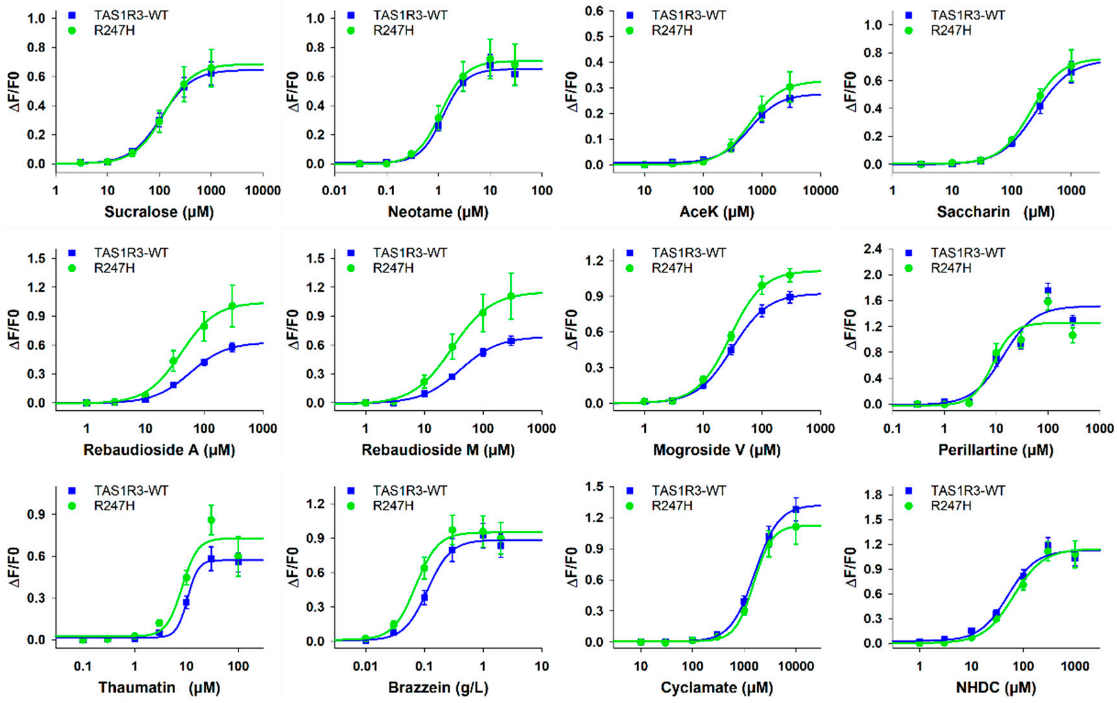

E

TAS1R3-G367C

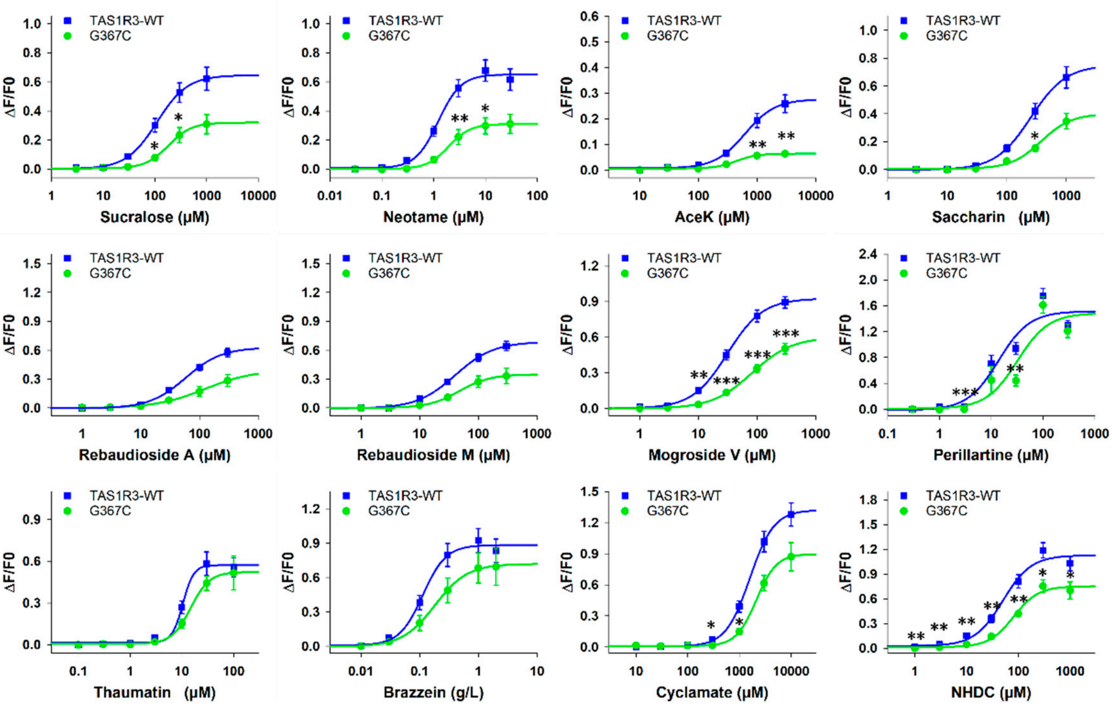

F

TAS1R3-S446N

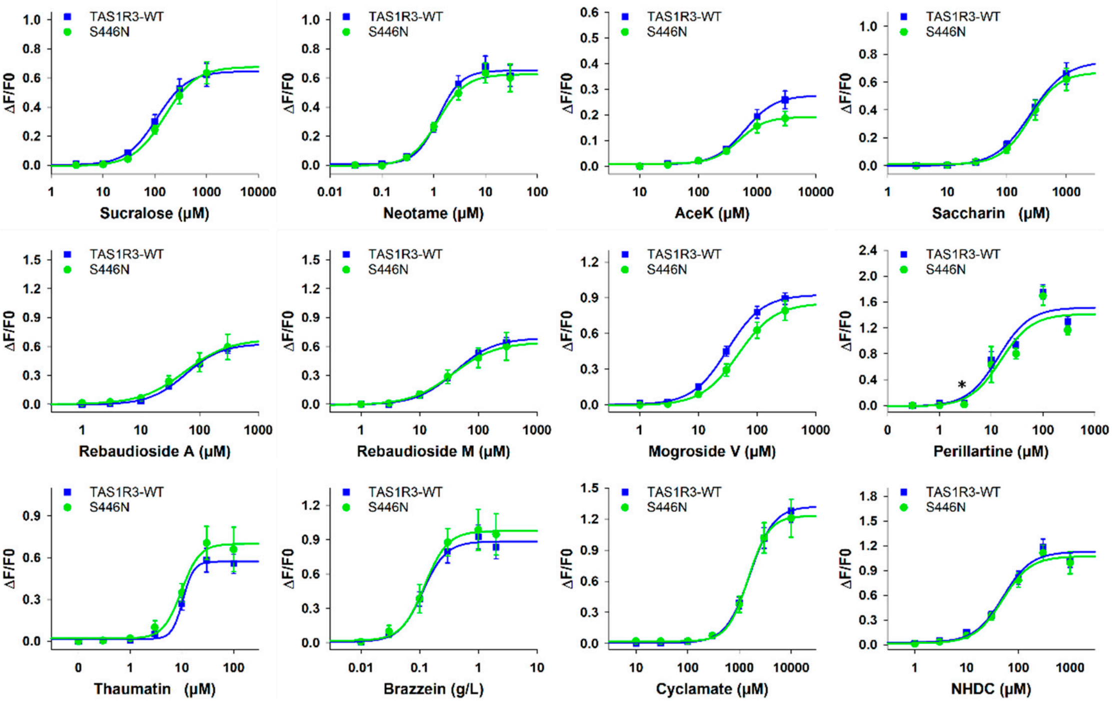

G

TAS1R3-F514L

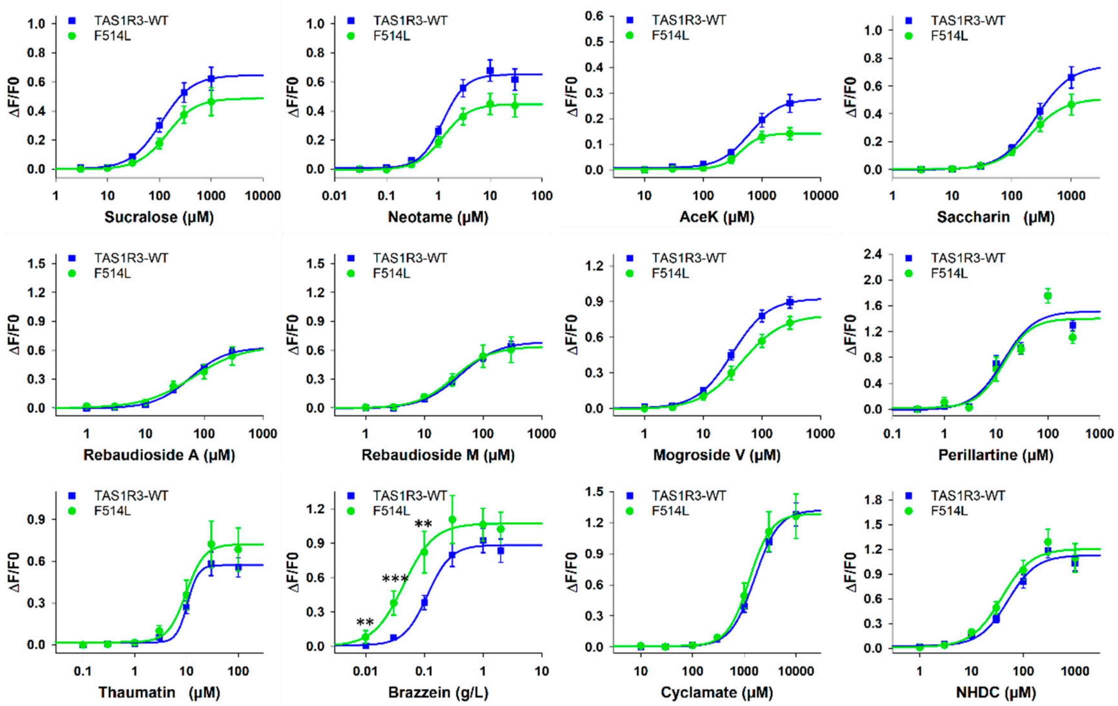

H

TAS1R3-S551N

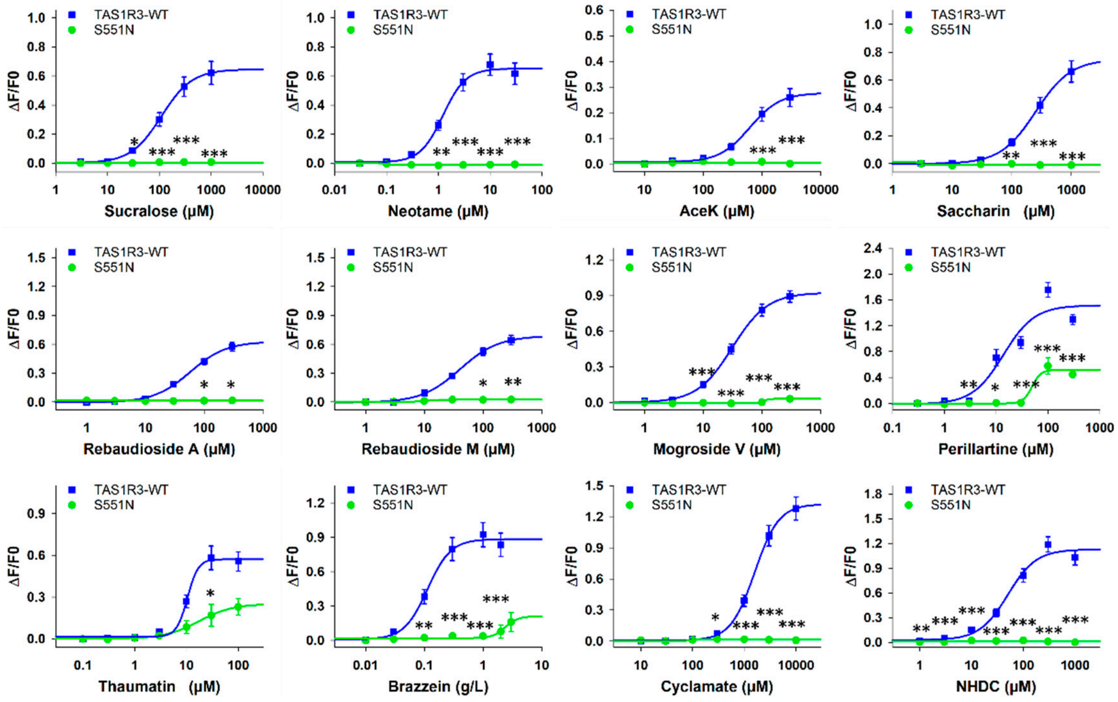

I

TAS1R3-T716M

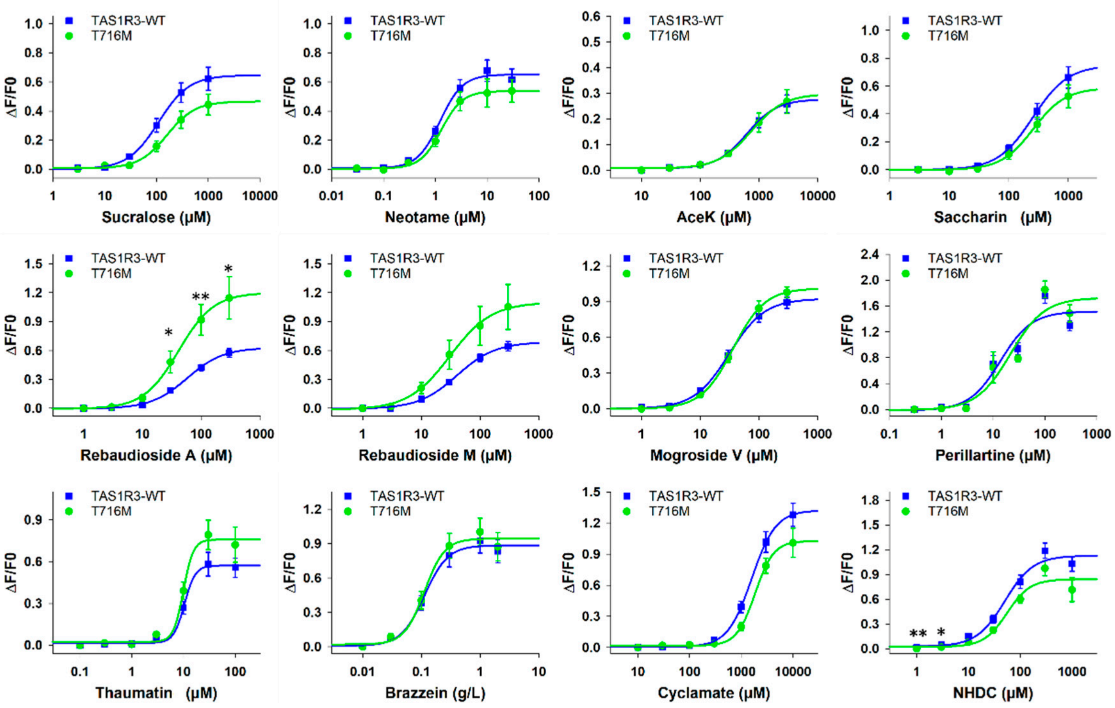

J

TAS1R3-A735T

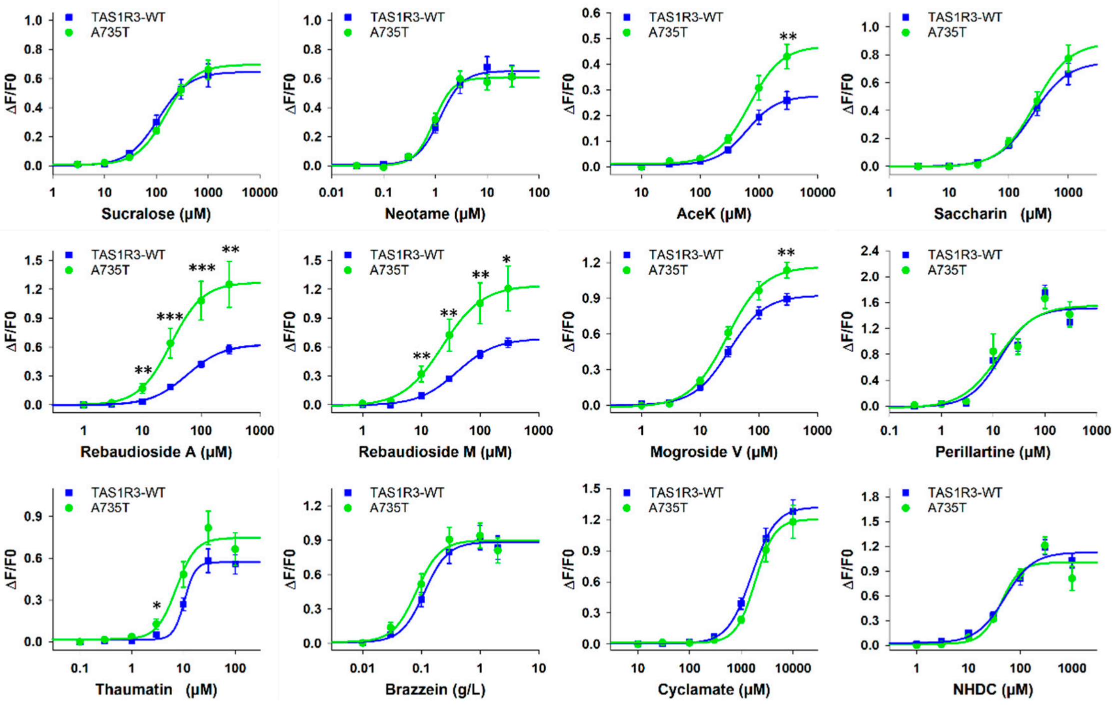

K

TAS1R3-F749S

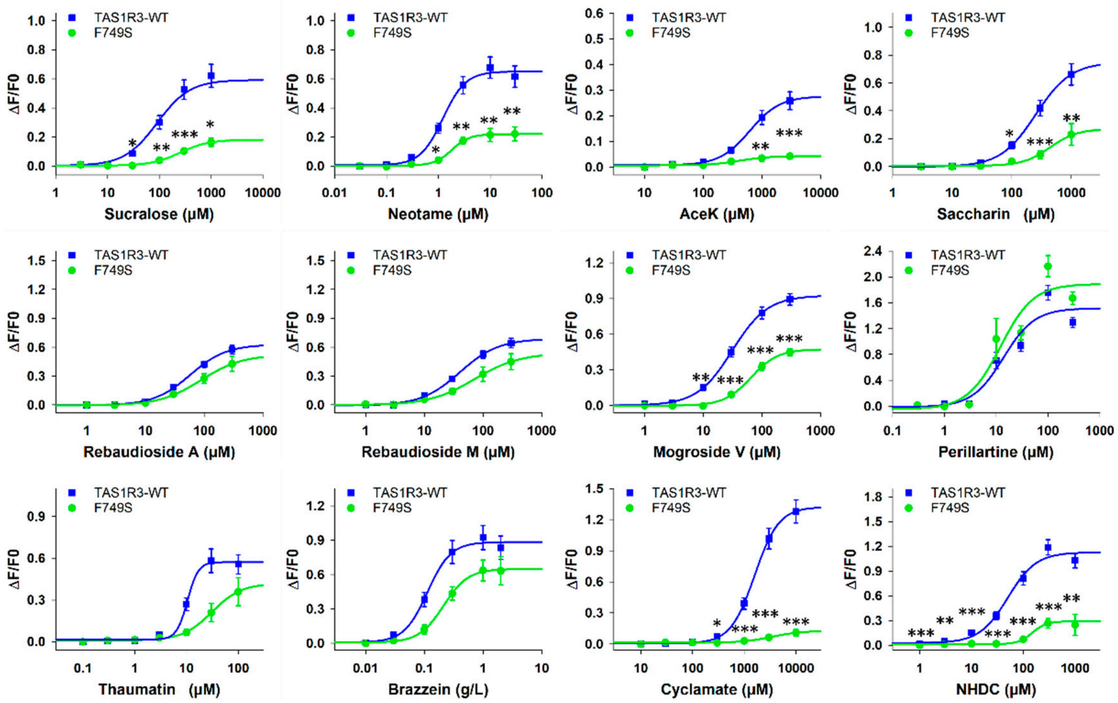

L

TAS1R3-C757R

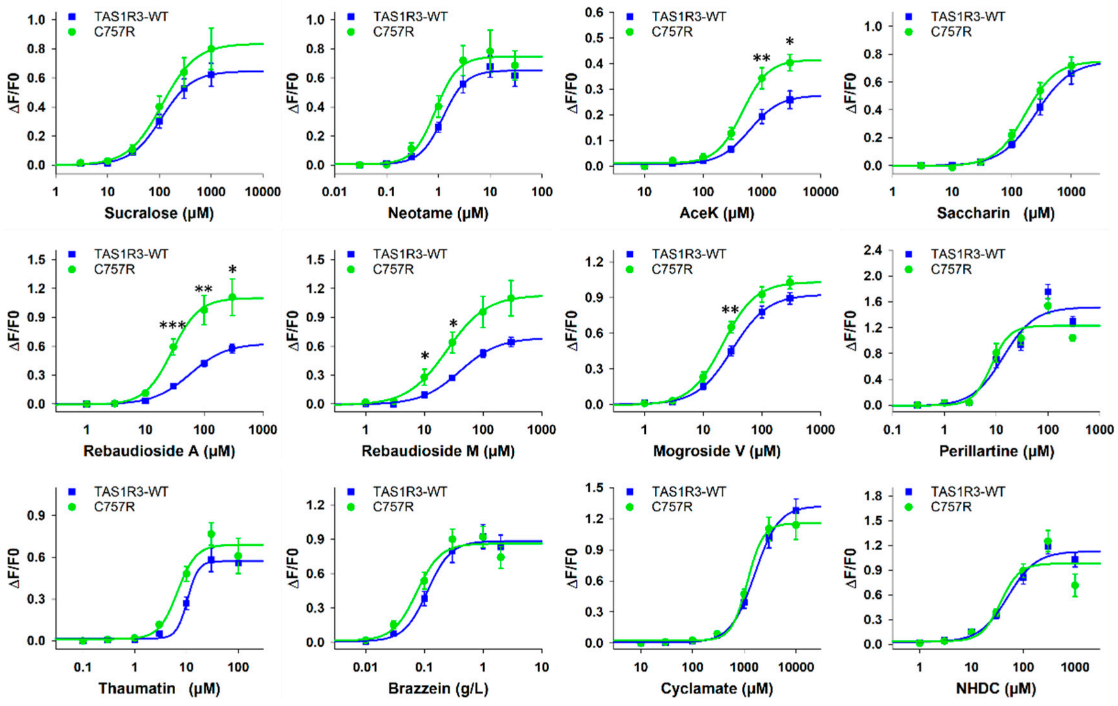

M

TAS1R3-P780A

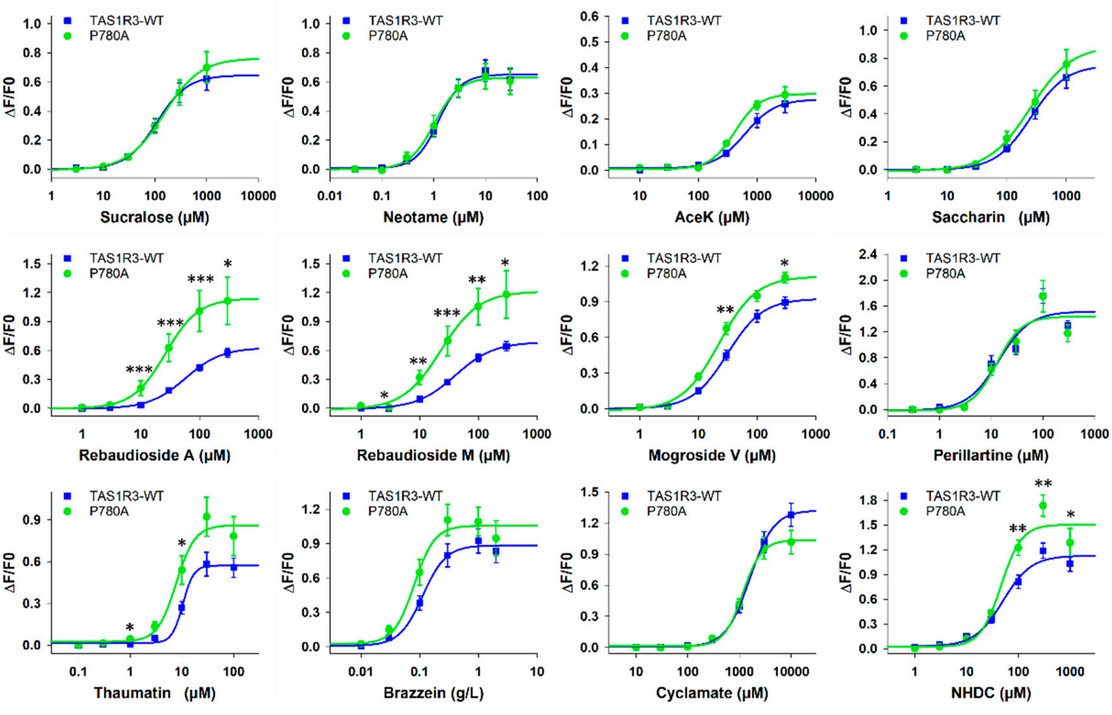

N

TAS1R3-R813K

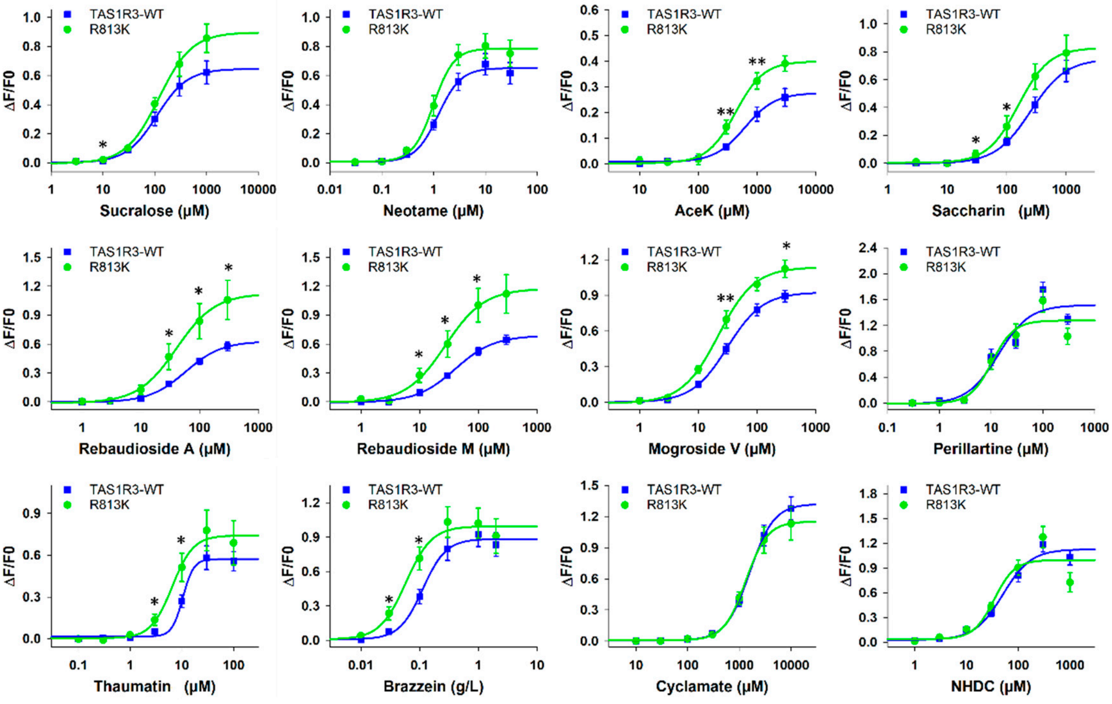

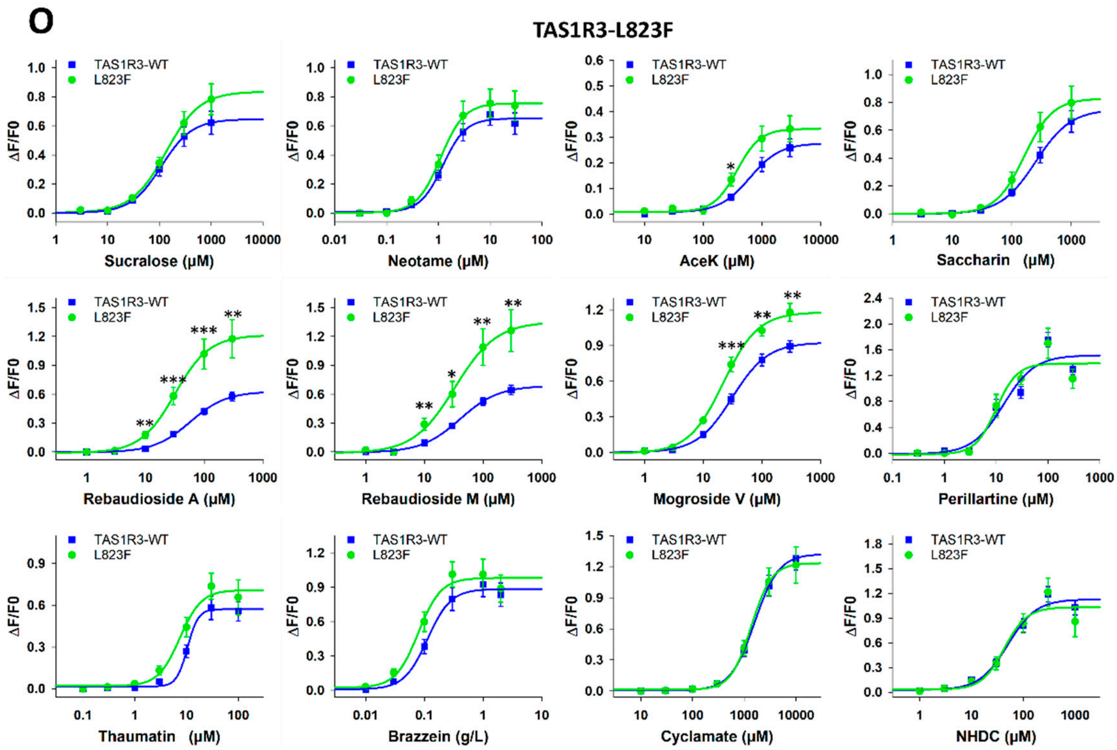

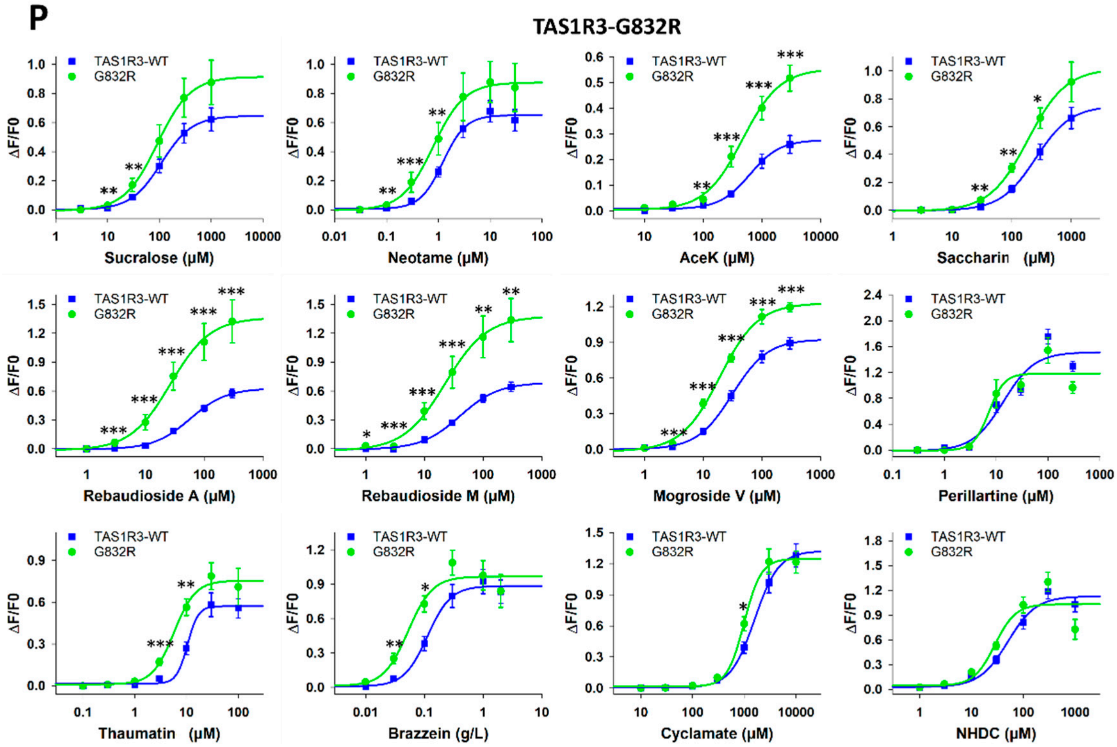

Figure S5. Human TAS1R2/TAS1R3-SNP dose-response curves with various sweeteners. HEK293T-Gα16gust44 cells were transiently transfected with pcDNA6-MAX-TAS1R2-FLAG and pcDNA4-MAX-TAS1R3-WT-FLAG (blue line) or pcDNA4-MAX-TAS1R3-SNP-FLAG (green line). A total of 16 TAS1R3 variants (A-P) and 12 sweeteners were tested. The data are presented as the mean ± sem of 8 wells from 4 independent experiments. * $p < 0.05$. ** $p < 0.01$. *** $p < 0.001$. calculated using ANOVA followed by Dunnett's test for multiple comparison analysis (with reference to TAS1R2-WT/TAS1R3-WT). The p-values are presented in Table S3. WT: wild-type; AceK: acesulfame K; NHDC: neohesperidin dihydrochalcone.

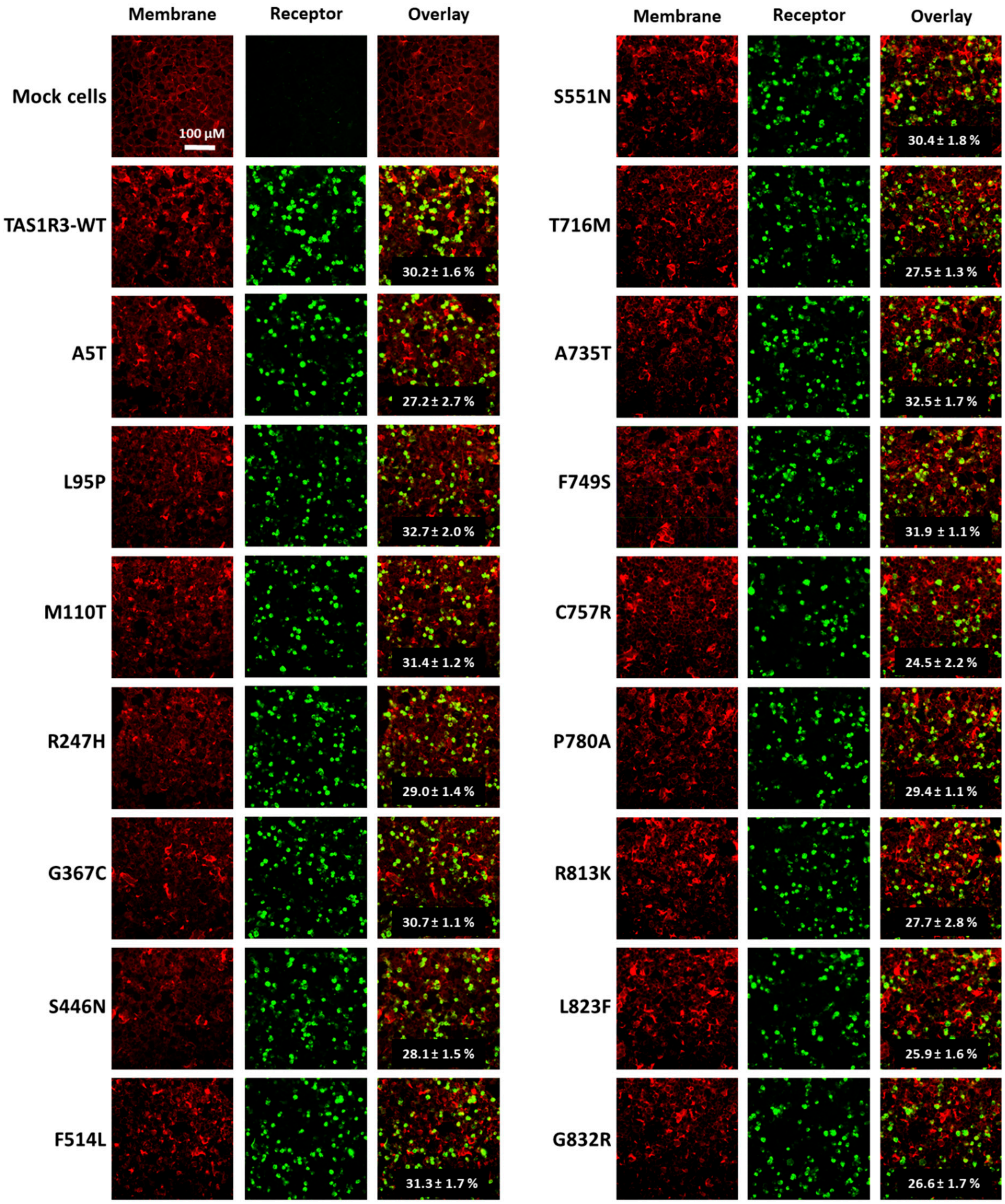

Figure S6. Immunocytochemistry of HEK293T-Gα16gust44 cells expressing FLAG-tagged TAS1R3-SNPs constructs. The TAS1R-expressing cells are shown in green. and the plasma membrane is stained in red. The receptors were detected using a primary anti-FLAG antibody and fluorescently labelled by a secondary Alexa-488–conjugated antibody. All data were obtained from the same transfection experiment. HEK293T-Gα16gust44 cells in the absence of the TAS1R receptor (mock cells) showed no signal. Pictures were taken using an epi-fluorescence inverted microscope (Eclipse TiE. Nikon. Champigny sur Marne. France) equipped with an x20 objective lens and a LucaR EMCCD camera (Andor Technology. Belfast. UK). The average cell fraction expressing the receptor (± sem) is provided in white in the overlay panel. Four to six images were counted. and averaged per receptor construct pictures. WT: wild-type.
